# Lactate‐Modulating Nanoreactors Facilitate Self‐Amplifying Pyroptosis and the cGAS‐STING Cascade for Potentiated Catalytic‐Immunotherapy

**DOI:** 10.1002/advs.202511144

**Published:** 2025-08-28

**Authors:** Linzhu Zhang, Shumin Sun, Duo Wang, Nailin Yang, Di Wang, Jihu Nie, Zifan Pei, Juan Qin, Lei Zhang, Haidong Zhu, Liang Cheng

**Affiliations:** ^1^ Center of Interventional Radiology & Vascular Surgery Department of Radiology Nurturing Center of Jiangsu Province for State Laboratory of AI Imaging & Interventional Radiology (Southeast University) Basic Medicine Research and Innovation Center of Ministry of Education Zhongda Hospital Medical School Southeast University 87 Dingjiaqiao Road Nanjing 210009 China; ^2^ Institute of Functional Nano & Soft Materials (FUNSOM) Jiangsu Key Laboratory for Carbon‐Based Functional Materials & Devices Soochow University Suzhou 215123 China

**Keywords:** cobalt fluoride (CoF_2_) nanoparticles, lactate metabolism, metalloimmunotherapy, pyroptosis, transarterial chemoembolization

## Abstract

The strategic induction of pyroptosis, coupled with the targeted potentiation of cGAS‐STING activation, represents a promising immunostimulatory approach. Herein, an intelligent lactate‐depleting LOCoF_2_ nanoreactor system is developed that orchestrated self‐amplifying pyroptosis induction and cGAS‐STING signaling potentiation for enhanced catalytic immunotherapy. This nanoplatform integrated cobalt fluoride (CoF_2_) nanoparticles with lactate oxidase (LOx), endowing it with dual enzymatic capabilities. Therefore, the LOCoF_2_ nanoreactor initially served as a pyroptosis activator, synergistically inducing pyroptosis in cancer cells by catalyzing the sequential generation of reactive oxygen species (ROS) while simultaneously inhibiting damage repair mechanisms. Subsequently, it functioned as an intelligent STING agonist to selectively detect pyroptosis‐released mitochondrial DNA (mtDNA) and augment the activation of the cGAS‐STING pathway. In vivo evaluations reveal that LOCoF_2_ administration provoked robust antitumor immunity, with synergistic effects observed when combined with immune checkpoint inhibitors, leading to significant regression of both primary and distant lesions. Notably, the LOCoF_2_‐lipiodol emulsion demonstrates exceptional therapeutic performance in a rat orthotopic hepatocellular carcinoma model when integrated with transcatheter arterial embolization (TAE) or transarterial chemoembolization (TACE) treatments. Therefore, the adverse factor lactate is ingeniously utilized to increase the activation of pyroptosis‐STING via a cascade reaction, ultimately achieving superior tumor control.

## Graphical abstract

An intelligent lactate‐depleting LOCoF_2_ (CoF_2_@LOx) nanoreactor system is developed to effectively induce self‐reinforced pyroptosis and activate the cGAS‐STING signaling pathway, thereby inducing a strong immune response and enhancing the therapeutic effectiveness of catalytic immunotherapy. Furthermore, the LOCoF_2_‐lipiodol dispersion exhibits significant therapeutic potential when combined with TAE/TACE in a rat orthotopic liver tumor model.

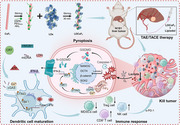

## 1 Introduction

Hepatocellular carcinoma (HCC) represents a significant cause of cancer‐related mortality globally, with its disease burden remaining consistently high.^[^ ^1^ ^]^ Local treatments such as transcatheter arterial embolization (TAE) or transarterial chemoembolization (TACE) are the preferred therapeutic modalities for patients with intermediate to advanced stage HCC.^[^ ^2^ ^]^ TAE/TACE involves percutaneous superselective catheterization of the hepatic arteries supplying hepatocellular carcinoma, followed by the administration of embolic agents and chemotherapeutic drugs.^[^ ^3^ ^]^ This approach disrupts the tumor's blood supply and induces ischemic necrosis. Despite frequent reductions in tumor burden, complete oncologic control remains uncommon, with therapeutic responses demonstrating significant interpatient variability. The development of chemoresistance, increased tumor invasiveness, and subsequent recurrence or metastasis are prevalent, collectively limiting objective response rates and long‐term survival outcomes. These challenges are largely attributable to incomplete ablation of malignant tissue and suboptimal pharmacokinetic profiles of administered agents.^[^ ^4^ ^]^ Traditional lipiodol‐based emulsions are hindered by inadequate physicochemical stability and rapid drug elution, impeding sustained locoregional drug delivery. In contrast, Pickering emulsions, stabilized by solid nanoparticles, markedly improve emulsion robustness and drug encapsulation, representing a promising platform to optimize the efficacy of TAE/TACE interventions.^[^ ^5^ ^]^ In recent years, tumor immunotherapy has undergone rapid advancements, pioneering a novel domain in anti‐tumor treatment by harnessing the immune system to elicit a durable anti‐tumor immune response.^[^ ^6^ ^]^ Therapeutic modalities such as immune checkpoint blockade (ICB) antibody therapy have demonstrated significant clinical benefits across various cancer types.^[^ ^7^ ^]^ Unfortunately, the efficacy of immunotherapy for HCC patients is limited to 20%–30%, primarily due to the intricate immunosuppressive tumor microenvironment (TME).^[^ ^8^ ^]^ Consequently, addressing the immunosuppressive TME and enhancing the effectiveness of immunotherapy represent critical challenges that require immediate attention.

The cGAS‐STING pathway constitutes a cytosolic DNA‐sensing mechanism that facilitates the production of type I interferons (IFNs) and other inflammatory cytokines, which play essential roles in the initiation of antitumor immune responses.^[^ ^9^ ^]^ Metal ions play crucial roles in immune regulation, and metalloimmunotherapy has emerged as a promising approach for the treatment of solid tumors.^[^ ^10^ ^]^ To date, several metal ions, including Mn^2+^, Zn^2+^, and Co^2+^, have been reported to exhibit significant cGAS‐STING activation capabilities.^[^ ^11^ ^]^ Notably, Co^2+^ enhances STING activity in a cGAMP‐dependent manner; in the absence of cGAMP, Co^2+^ fails to activate STING.^[^ ^11a^ ^]^ This highlights the potential of Co‐based STING agonists to precisely modulate the immune response, considering that aberrant activation of the cGAS‐STING pathway has been implicated in the development of inflammatory and degenerative diseases. However, elevated levels of aberrant metabolites, particularly lactate at concentrations ranging from 10 to 30 mm in tumor tissues, can hinder the activation of the cGAS‐STING pathway.^[^ ^12^ ^]^ Specifically, lactate promotes the lactylation of cGAS, thereby inhibiting the production of cGAMP and consequently attenuating innate immune surveillance.^[^ ^13^ ^]^ Moreover, lactate facilitates DNA repair processes in cancer cells, further suppressing the activation of the cGAS‐STING pathway.^[^ ^14^ ^]^ Simultaneously, lactate produced through the Warburg effect functions as a pivotal intrinsic regulator of tumorigenesis, facilitating the enrichment of the cancer stem cell compartment by inhibiting their differentiation and reprogramming differentiated cancer cells toward a proliferative cancer stem cell phenotype.^[^ ^15^ ^]^ Therefore, it is crucial to develop efficacious strategies for the delivery of Co‐based agonists and tumor‐specific exposure of mitochondrial DNA (mtDNA), while simultaneously modulating lactate metabolism within the TME.

Pyroptosis is a novel form of inflammatory programmed cell death mediated by gasdermin (GSDM) family proteins.^[^ ^16^ ^]^ This process is characterized by cytoplasmic swelling, culminating in membrane rupture and the subsequent release of intracellular contents, thereby eliciting an immune response.^[^ ^17^ ^]^ Before plasma membrane disruption, GSDMD‐N translocates and damages mitochondria, leading to the release of mtDNA, which serves as a critical activator of the cGAS‐STING pathway.^[^ ^18^ ^]^ Consequently, inducing high‐efficiency pyroptosis in cancer cells may serve as an upstream event that triggers cGAS‐STING activation. Emerging evidence indicates that specific metal ions (including Zn^2+^ and Co^2+^) and reactive oxygen species (ROS) can effectively induce pyroptosis in malignant cells.^[^ ^19^ ^]^ Tumor catalytic therapy has emerged as a promising strategy that harnesses exogenous triggers or TME responses to transform relatively benign endogenous biomolecules into potent cytotoxic ROS through precisely controlled catalytic reactions, enabling tumor‐specific cytotoxicity with spatiotemporal precision.^[^ ^20^ ^]^ In this strategy, the Co‐based nanocatalyst demonstrates TME‐activated cascade catalytic functionality, effectively orchestrating catalase‐like functions to convert endogenous hydrogen peroxide (H_2_O_2_) into ROS, thereby triggering self‐amplifying oxidative damage cascade for tumor‐specific eradication.^[^ ^21^ ^]^ However, the limited availability of H_2_O_2_ in the TME constrains the sustained efficacy of this therapeutic approach.^[^ ^22^ ^]^ More critically, elevated levels of lactate facilitate the ability of cancer cells to repair damaged DNA, maintain genomic stability, promote tumor cell stemness, and potentially inhibit pyroptosis.^[^ ^14^, ^23^ ^]^ Therefore, the development of efficient strategies to modulate lactate levels and increase H_2_O_2_ concentrations within tumors, thereby increasing ROS generation, is beneficial for inducing pyroptosis in tumor cells.

**Scheme 1:**
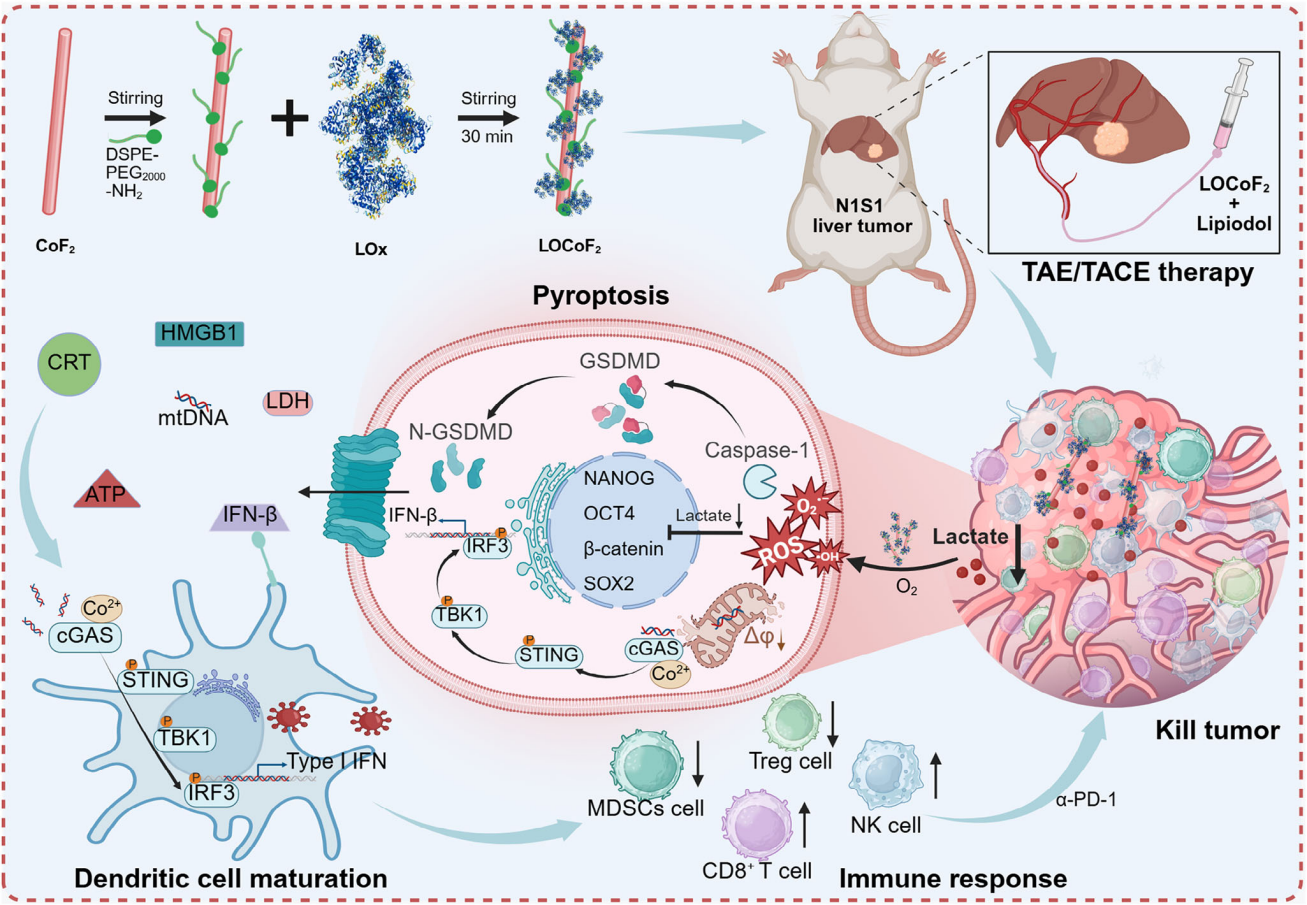
Schematic representation of LOCoF_2_ lactate‐modulating nanoreactors enabling self‐amplifying pyroptosis and cGAS‐STING cascade activation for enhanced catalytic immunotherapy.

While lactate‐deleting nanoreactors have been developed for the treatment of tumors, limited attention has been given to the potential interactions between lactate and key biological pathways, including STING signaling and pyroptosis.^[^ ^12^, ^24^ ^]^ Drawing upon these bottlenecks and challenges, we engineered intelligent lactate‐depleting nanoreactors that effectively mediated the induction of self‐reinforced pyroptosis and cGAS‐STING signaling cascades, thereby increasing the efficacy of catalytic immunotherapy (**Scheme** **1**). Specifically, the LOCoF_2_ nanoreactor, constructed by integrating cobalt fluoride (CoF_2_) nanocatalysts with lactate oxidase (LOx), exhibited both LOx and peroxidase‐like activities. Consequently, the LOCoF_2_ nanoreactor initially functions as a pyroptosis activator. LOx metabolized lactate within the TME, generating H_2_O_2_ that served as a substrate for CoF_2_‐mediated ROS production while simultaneously inhibiting damage repair mechanisms. Subsequently, the LOCoF_2_ nanoreactor was further employed as an intelligent STING agonist to selectively detect pyroptosis‐released mtDNA and augment the activation of the cGAS‐STING pathway. The administration of LOCoF_2_ into tumors elicited a robust immune response, providing substantial support for antitumor therapy. Therefore, the combination of LOCoF_2_ with ICB resulted in significant suppression of both primary and metastatic tumors. Moreover, the LOCoF_2_‐lipiodol dispersion demonstrated remarkable efficacy when used in conjunction with TAE/TACE in a rat orthotopic liver tumor model. This innovative approach leverages the metabolic properties of tumors to transform lactate, a key immunosuppressive metabolite, into a therapeutic asset through a series of catalytic reactions, thereby enhancing pyroptosis‐STING coordination and ultimately achieving superior tumor control, offering new insights into tumor therapy.

## 2 Results and Discussion

### 2.1 Lactate Augmented DNA Repair and Conferred Resistance to DNA‐Damaging Therapy in HCC

**Figure 1:**
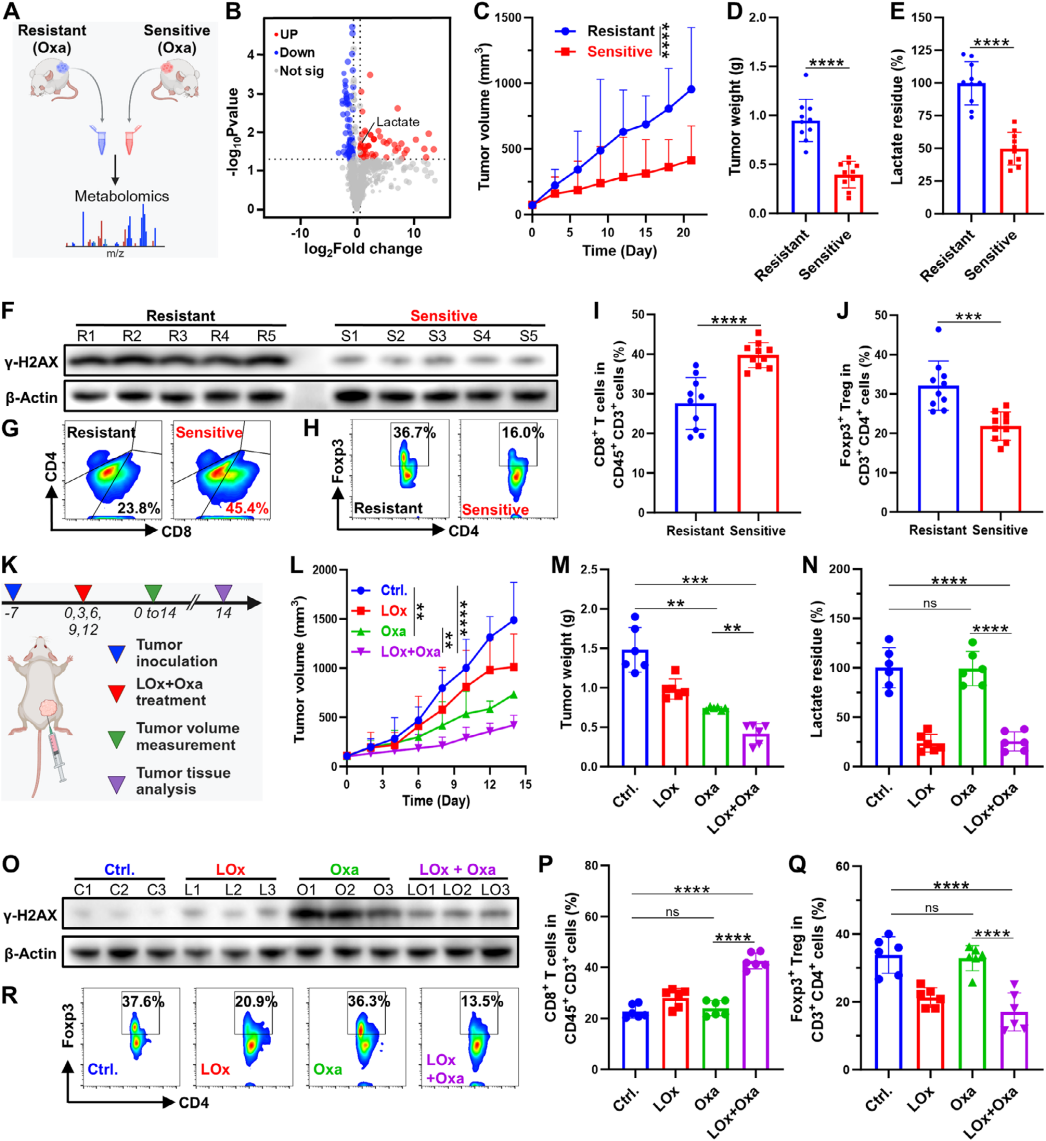
Elevated lactate levels enhanced DNA repair and promoted resistance to DNA‐damaging therapies in HCC. A) Schematic diagram of non‐targeted metabolomics analysis of H22 tumor tissue. B) Volcano plot of metabolomic differences between Oxa‐resistant (*n* = 6) and Oxa‐sensitive (*n* = 4) H22 tumor tissues. C,D) Oxa‐resistant and Oxa‐sensitive H22 tumor volume C) and tumor weight D). E) Lactate content in Oxa‐resistant and Oxa‐sensitive H22 tumors. F) The expression levels of γ‐H2AX in Oxa‐resistant and Oxa‐sensitive tumor tissues were detected via Western blot analysis. G) Flow cytometric analysis of CD45^+^CD3^+^CD8^+^ T cells. H) Flow cytometry analysis of Tregs (CD3^+^CD4^+^Foxp3^+^) in Oxa‐resistant and Oxa‐sensitive tumors. I) Statistical data of (G). J) Statistical data of (H). K) Schematic illustration of the in vivo therapeutic regimen. L) Tumor volume changes after different treatments. M) Tumor weight variation after different treatments. N) Lactate contents in tumor tissues after different treatments. O) Western blot analysis of γ‐H2AX expression in tumors subjected to different treatments. P) Statistical data of CD45^+^CD3^+^CD8^+^ T cells in tumor tissues after different treatments. Q) Statistical data of CD3^+^CD4^+^Foxp3^+^ T cells in tumor tissues after different treatments. R) Flow cytometry assessment of CD3^+^ CD4^+^ Foxp3^+^ cells within tumors after different treatments. The data represent mean  ±  SD, *n* = 10 biologically independent samples in (D, E, I–J), *n* = 6 biologically independent samples in (M–N, P–Q). one‐way analysis of variance (ANOVA) was used for multiple comparisons among three or more groups. ^**^ *p* < 0.01, ^***^ *p* < 0.001, ^****^ *p* < 0.0001.

To investigate the potential relationship between lactate and the efficacy of DNA‐damaging therapies such as chemotherapy, oxaliplatin (Oxa) was administered to H22 tumor‐bearing mice. Among the ten mice treated with Oxa for 21 days, six exhibited relatively rapid tumor growth (designated as the chemotherapy‐resistant group), while the remaining four exhibited slower tumor progression (designated the chemotherapy‐sensitive group) (**Figure** **1**A). Tumor tissues were subsequently extracted for non‐targeted metabolomics analysis, which identified lactate as one of the most abundant metabolites in drug‐resistant tumors (Figure 1B). To further validate the aforementioned findings, we conducted Oxa chemotherapy on a cohort of 20 mice. Following a 21‐day treatment regimen, the tumor volume in 10 mice presented relatively rapid growth, which we designated the chemotherapy‐resistant group, while the remaining 10 mice presented slower tumor growth, which we designated as the chemotherapy‐sensitive group (Figure 1C,D; Figures S1,S2, Supporting Information). The tumor volume and weight in the chemotherapy‐sensitive group were significantly lower than those in the treatment‐resistant group. Lactate levels in tumor tissues were measured via a lactate assay kit, demonstrating significantly lower concentrations in chemotherapy‐sensitive tumors than in resistant tumors (Figure 1E), corroborating the metabolomics results. DNA‐damaging chemotherapy induces double‐strand breaks (DSBs), triggering rapid phosphorylation of histone H2AX at serine 139 (γH2AX) at break sites.^[^ ^14^ ^]^ Compared with sensitive tumors, the chemotherapy‐resistant tumors presented markedly elevated γH2AX expression (Figure 1F; Figures S3,S4, Supporting Information). The results suggested that lactate was involved primarily in homologous recombination (HR)‐mediated DNA repair. Flow cytometry analysis revealed that the proportion of CD8^+^ T cells was significantly greater in the treatment‐sensitive group than in the treatment‐resistant group (Figure 1G,I; Figure S5, Supporting Information). Additionally, the number of regulatory T cells (Tregs), which exert immunosuppressive functions, was lower in the treatment‐sensitive group than in the treatment‐resistant group (Figure 1H,J; Figure S6, Supporting Information). These findings suggested that lactate may contribute to resistance to Oxa chemotherapy by promoting HR‐mediated DNA repair and promoting a tumor immunosuppressive microenvironment.^[^ ^14^ ^]^

To further investigate whether the consumption of excess lactate in tumors can increase the effectiveness of DNA‐damaging therapies such as chemotherapy, we employed a strategy that combined LOx with Oxa. H22 tumor‐bearing mice were randomized into four experimental groups: group I, the control group; group II, the LOx group; group III, the Oxa group; and group IV, the LOx + Oxa group. Every three days, the mice were intraperitoneally injected with Oxa and intratumorally injected with LOx (Figure 1K). The results of the tumor volume and weight analysis demonstrated that LOx or Oxa alone had a moderate inhibitory effect on tumor growth compared with that of the control group, whereas the combined administration of LOx and Oxa resulted in a significantly increased inhibitory effect on tumor growth (Figure 1L,M; Figures S7,S8, Supporting Information). Further analysis revealed that LOx significantly diminished lactate levels in tumor tissues, potentially enhancing the efficacy of therapeutic interventions (Figure 1N). One of the primary mechanisms by which lactate confers resistance to therapeutic effects is through its ability to enhance DNA damage repair. Western blot analysis revealed markedly attenuated DNA damage repair capacity in the LOx + Oxa group compared with the Oxa monotherapy group (Figure 1O; Figures S9,S10, Supporting Information). These findings indicated that the consumption of lactate led to a decrease in DNA repair efficiency, thereby improving the chemotherapeutic effect of Oxa. Flow cytometry analysis revealed a significantly greater proportion of CD8^+^ T cells in the LOx + Oxa group than in the Oxa group (Figure 1P; Figure S11, Supporting Information), while the number of immunosuppressive Tregs was notably lower in the LOx + Oxa group than in the Oxa group (Figure 1Q,R). These findings suggested that the reduction in lactate in tumor tissues not only inhibited DNA damage repair and enhanced the efficacy of chemotherapy but also reversed tumor‐associated immunosuppression.

### 2.2 Preparation and Characterization of LOx@CoF_2_ (LOCoF_2_) Nanoreactors

**Figure 2:**
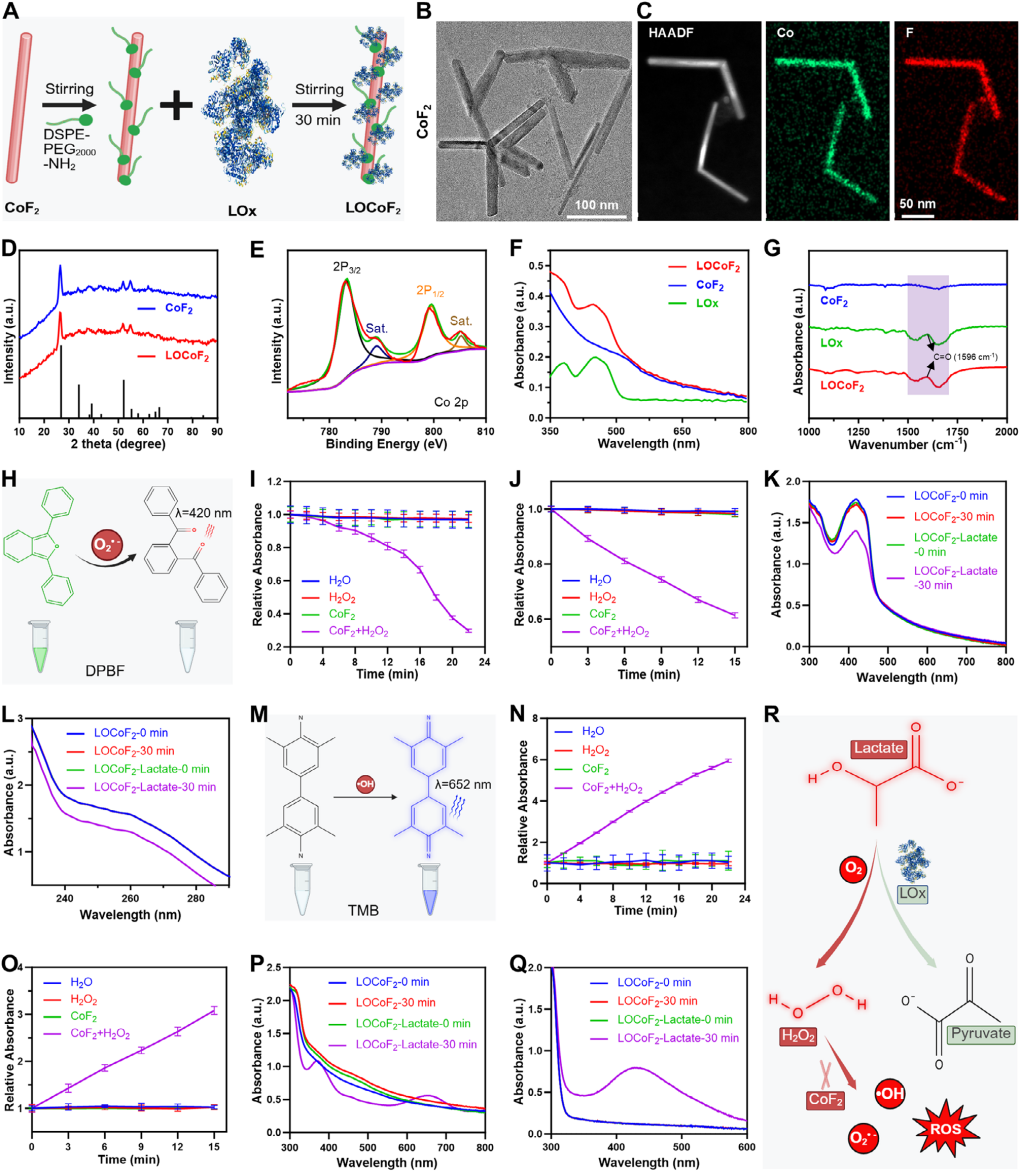
Synthesis and characterization of LOCoF_2_. A) Schematic diagram of the synthesis of LOCoF_2_. B) TEM images of the CoF_2_ NPs. C) HAADF‐STEM image and CoF_2_ element distribution (F, Co). D) XRD patterns of CoF_2_ and LOCoF_2_. E) XPS spectrum of CoF_2_. F) UV–vis spectra of LOx, CoF_2_, and LOCoF_2_. G) FT‐IR spectra of LOx, CoF_2_, and LOCoF_2_. H) Diagram of the reaction of green DPBF with O_2_^•−^ to form a colorless product. I) Time‐dependent oxidation of DPBF with different treatments. J) Time‐dependent oxidation of NBT by the CoF_2_ under H_2_O_2_. K) Time‐dependent oxidation of DPBF by LOCoF_2_ NPs under lactate. L) Time‐dependent oxidation of NBT by the LOCoF_2_ under lactate. M) Diagram of ·OH mediated TMB oxidation to blue oxTMB. N) Time‐dependent oxidation of TMB with different treatments. O) The OPD probe was used to study the time‐dependent changes in the ·OH generation efficiency of CoF_2_ under H_2_O_2_. P) Time‐dependent oxidation of TMB by LOCoF_2_ NPs under lactate. Q) The time‐dependent changes of ·OH generation efficiency of the LOCoF_2_ in the presence of lactate were investigated using a OPD probe. R) Schematic diagram of lactate catalyzed by LOx to generate H_2_O_2_, which was then oxidized into ROS by CoF_2_ NPs.

First, rod‐shaped CoF_2_ was prepared via high‐temperature thermal decomposition using cobalt acetylacetonate and ammonium fluoride (NH_4_F) as the cobalt and fluorine precursors. Subsequently, DSPE‐PEG_2000_‐NH_2_ modification facilitated the electrostatic interaction‐driven self‐assembly of LOx on the CoF_2_ surface, resulting in the formation of LOCoF_2_ nanoreactors (**Figure** **2**A). Transmission electron microscopy (TEM) revealed that the CoF_2_ nanoparticles (NPs) exhibited a uniform size distribution and a rod‐like morphology (Figure 2B). Energy dispersive X‐ray spectroscopy (EDS) elemental mapping analysis indicated that Co and F were homogeneously distributed throughout the nanoparticles (Figure 2C). In addition, the crystal structure diffraction peaks of the CoF_2_ NPs were characterized by X‐ray diffraction (XRD). The obtained diffraction patterns matched those of CoF_2_ (JCDPS 33‐0417), confirming the successful synthesis of CoF_2_ (Figure 2D). X‐ray photoelectron spectroscopy (XPS) was subsequently employed to investigate the surface elemental composition and electronic states of CoF_2_ (Figure 2E; Figure S12, Supporting Information). The observed peaks at 797.88 and 781.48 eV corresponded to Co 2p1/2 and Co 2p3/2, respectively, with accompanying shake‐up satellite peaks at 803.38 and 787.18 eV, which were consistent with previously reported XPS spectra for Co (II). Additionally, the presence of the F 1s peak at ≈684.18 eV indicated the formation of an F‐metal bond. These results indicated that rod‐like CoF_2_ NPs were successfully synthesized through a high‐temperature thermal decomposition method. To further integrate CoF_2_ and LOx, DSPE‐PEG_2000_‐NH_2_ modification was employed to develop LOCoF_2_ nanoreactors. The CoF_2_ modified with DSPE‐PEG_2000_‐NH_2_ acquired a pronounced positive charge, enabling the adsorption of LOx onto its surface through electrostatic interactions, thereby forming LOCoF_2_. Zeta potential and dynamic light scattering (DLS) measurements collectively verified the effective conjugation of LOx with CoF_2_ NPs (Figures S13,S14, Supporting Information). UV–vis spectra revealed a distinct LOx absorption peak for LOCoF_2_, whereas no absorption signature was observed for CoF_2_ (Figure 2F). Furthermore, Fourier transform infrared spectroscopy (FT‐IR) analysis demonstrated that LOCoF_2_ displayed C═O bond peaks, with a distinctive absorption band within the 1500–1700 cm^−1^ region, closely resembling the spectral features observed for LOx (Figure 2G).^[^ ^25^ ^]^ To further investigate the potential impact of CoF_2_ on LOx activity, lactate was separately co‐incubated with both LOx and LOCoF_2_. Both LOx and LOCoF_2_ significantly decreased the concentration of lactate, suggesting that CoF_2_ does not alter the catalytic activity of LOx (Figure S15, Supporting Information). All these results demonstrated the successful synthesis of LOCoF_2_ nanoreactors with enzyme‐like activity, indicating their promising application potential in cancer therapy.

Next, we systematically investigated the enzyme‐like activity of CoF_2_ and LOCoF_2_, considering the elevated levels of lactate and H_2_O_2_ within the TME. We evaluated the catalytic performance of both CoF_2_ and LOCoF_2_ NPs with a 1,3‐diphenylisobenzofuran (DPBF) probe to detect the generation of superoxide anions (O_2_^•−^). Specifically, DPBF, which is green in color, reacts with O_2_^•−^ to produce a colorless product (Figure 2H). As expected, only the DPBF in the H_2_O_2_ + CoF_2_ group presented a significant reduction in UV–vis absorption at ≈420 nm with increasing reaction time, demonstrating efficient generation of O_2_^•−^ (Figure 2I; Figure S16, Supporting Information). Furthermore, nitro blue tetrazolium (NBT) was employed to assess the production of O_2_^•–^. A time‐dependent reduction in NBT absorbance was detected in the H_2_O_2_ + CoF_2_ group after 15 min, demonstrating efficient O_2_^•−^ generation (Figure 2J; Figure S17, Supporting Information). Subsequently, the DPBF probe was utilized to investigate the catalytic conversion of lactate by LOx to H_2_O_2_, which was subsequently decomposed by CoF_2_ to yield O_2_^•−^. Notably, the UV–visible absorbance of DPBF at 420 nm markedly decreased over time exclusively in the lactate + LOCoF_2_ group, confirming the generation of O_2_^•−^ via the reaction between lactate and LOCoF_2_ (Figure 2K; Figure S18, Supporting Information). Similarly, the absorbance of NBT at about 260 nm was significantly reduced (Figure 2L; Figure S19, Supporting Information). Additionally, 3,5,3′,5′‐tetramethylbenzidine (TMB) was used as a chromogenic substrate to quantitatively evaluate the peroxidase‐mimetic activity of the LOCoF_2_ nanoreactor by monitoring its capacity to catalyze the formation of hydroxyl radicals (·OH) from H_2_O_2_ (Figure 2M). The H_2_O_2_ + CoF_2_ group exhibited a pronounced absorption peak at ≈652 nm, substantiating the peroxidase‐like catalytic function of CoF_2_ nanoparticles (Figure 2N; Figure S20, Supporting Information). Similarly, the characteristic absorbance of o‐phenylenediamine (OPD) increased significantly over time in the H_2_O_2_ + CoF_2_ group, further indicating robust ·OH generation (Figure 2O; Figure S21, Supporting Information). To evaluate the cascade catalytic performance of LOCoF_2_, a distinct TMB absorption peak was observed solely in the lactate + LOCoF_2_ group (Figure 2P; Figure S22, Supporting Information), a finding corroborated by additional OPD probe assays (Figure 2Q; Figure S23, Supporting Information). Collectively, these findings indicated that LOCoF_2_ nanoreactors could function as self‐reinforcing catalysts to significantly increase the production of ROS within the TME by increasing the concentration of H_2_O_2_ through the utilization of lactate as a substrate (Figure 2R), indicating excellent biomedical application prospects.

### 2.3 In Vitro Catalytic Therapy via LOCoF_2_ Nanoreactors

**Figure 3:**
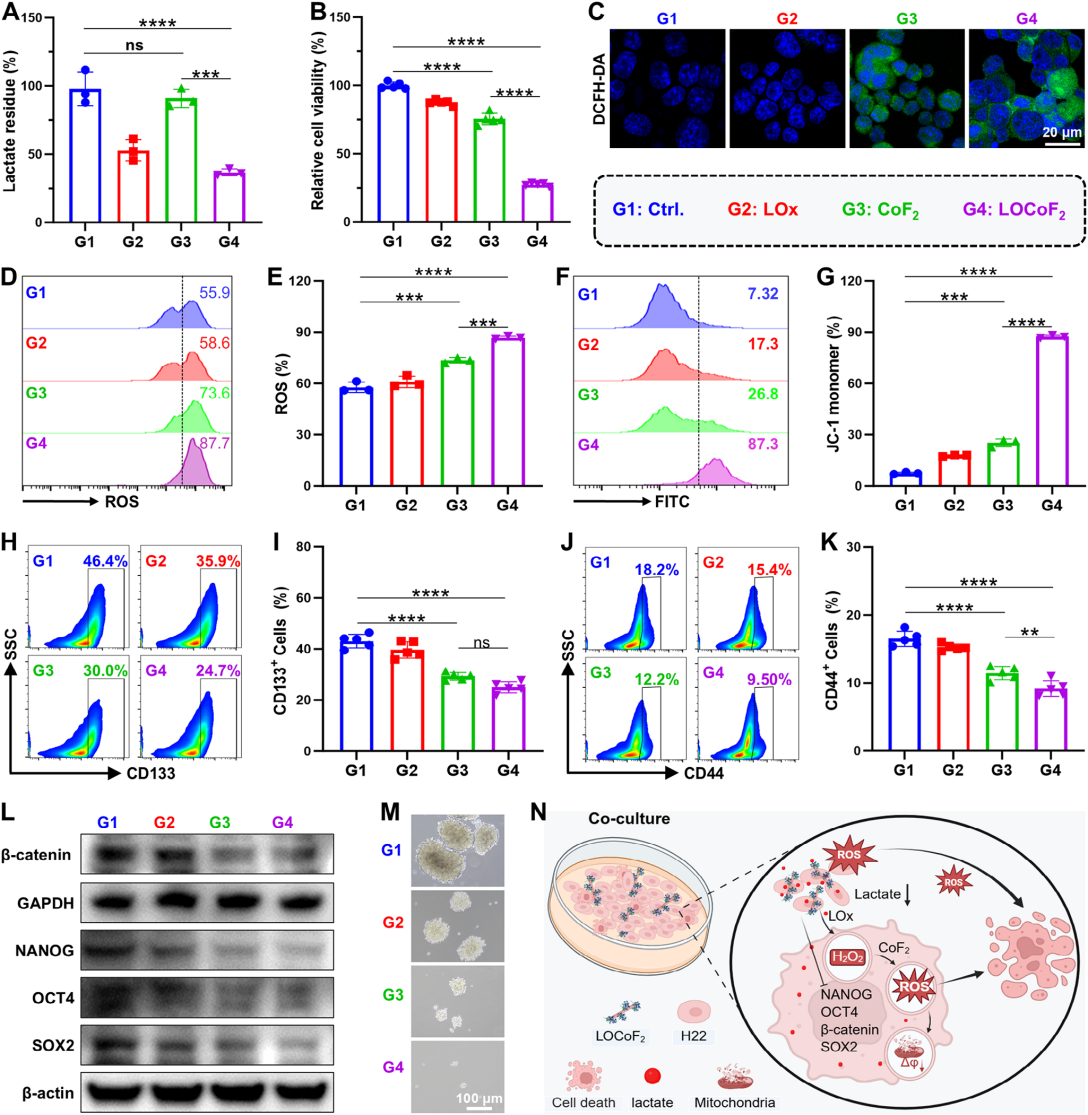
LOCoF_2_ induced cell death by amplifying oxidative stress and simultaneously attenuated cancer stemness. A) Relative concentrations of lactate after different treatments. G1: Ctrl., G2: LOx, G3: CoF_2_, G4: LOCoF_2_. B) Relative viability of H22 cells after various treatments. C) Confocal microscope images of ROS in H22 cells subjected to different treatments. D) Flow cytometric assessment and E) quantitative analysis of ROS levels in H22 cells after various treatments. F) Flow cytometric assessment and G) quantitative analysis of the mitochondrial membrane potential in H22 cells after various treatments. H) Flow cytometry analysis and I) quantification of CD133^+^ cells. J) Flow cytometry analysis and K) quantification of CD44^+^ cells. L) The expression levels of β‐catenin, NANOG, OCT4 and SOX2 in H22 cells were detected by western blotting. M) A sphere formation assay was used to evaluate the in vitro self‐renewal ability. N) The diagram illustrates the mechanism by which LOCoF_2_ kills tumor cells and inhibits stemness by consuming lactate to produce ROS. The data represent mean  ±  SD, *n* = 3 biologically independent samples in (A, D–G), *n* = 5 biologically independent samples in (B, H‐K). ANOVA was used for multiple comparisons among three or more groups. ^**^ *p* < 0.01, ^***^ *p* < 0.001, ^****^ *p* < 0.0001.

The superior lactate consumption and ROS generation capabilities of LOCoF_2_ nanoreactors endow them with significant potential for effectively eliminating cancer cells. First, the alterations in lactate levels in H22 cells were quantified 12 h post‐treatment, as effective cellular lactate consumption is crucial for the efficacy of the treatments. Both LOx and LOCoF_2_ markedly decreased the lactate concentration in H22 cells (**Figure** **3**A). The results of the CCK8 assay demonstrated that LOx had only a minor influence on cell viability, while both CoF_2_ and LOCoF_2_ diminished cell activity, with LOCoF_2_ exhibiting a more pronounced effect (Figure 3B). To validate ROS induction, H22 cells were subjected to 2′,7′ dichlorodihydrofluorescein diacetate (DCFH‐DA) fluorescence assay. H22 cells treated with CoF_2_ displayed robust green fluorescence, while those exposed to LOCoF_2_ presented the most intense green fluorescence, indicating efficient intracellular ROS generation (Figure 3C). Flow cytometry analysis further confirmed these findings, demonstrating markedly elevated intracellular ROS in LOCoF_2_‐treated cells (Figure 3D&E). Excessive intracellular ROS can induce mitochondrial dysfunction, ultimately resulting in extensive cell death. The mitochondrial membrane potential was further assessed via the JC‐1 probe. During the damage process, JC‐1 transitioned from red fluorescent aggregates to green fluorescent monomers. As expected, after incubation with CoF_2_, the monomer ratio in H22 cells increased from ≈7.32% to ≈26.8%, while this ratio significantly increased to ≈87.3% in the LOCoF_2_ group (Figure 3F,G). Furthermore, flow cytometric quantification of annexin V‐FITC/PI double‐stained H22 cells confirmed the substantial induction of cell death following LOCoF_2_ treatment (Figures S24,S25, Supporting Information). Elevated levels of lactate contribute to increased proliferation of cancer stem cells, resulting in immunosuppressive effects and enhanced resistance to therapeutic interventions.^[^ ^15^, ^23^ ^]^ Therefore, we further examined the regulatory effects of the LOCoF_2_ system on the stemness characteristics of H22 cells.^[^ ^26^ ^]^ Quantitative flow cytometric analysis revealed a 21.7% decrease in CD133 expression and a significant 8.7% reduction in CD44 expression in H22 cells following LOCoF_2_ treatment (Figure 3H–K; Figures S26,S27, Supporting Information). To further validate the inhibitory effect of LOCoF_2_ on tumor stemness, H22 cells were co‐incubated with LOCoF_2_ for 24 h. Subsequently, cellular proteins were extracted and subjected to Western blot analysis to assess alterations in the expression levels of proteins associated with cell stemness. The results demonstrated that treatment with LOCoF_2_ significantly diminished the expression of key stemness markers, including β‐catenin, NANOG, OCT4, and SOX2 (Figure 3L; Figures S28,S29, Supporting Information). Additionally, the sphere formation assay revealed that the diameter of H22 cell spheres following LOCoF_2_ treatment was restricted to 6–14 µm, in contrast to the control group, where spheroid diameters reached 117–190 µm (Figure 3M; Figure S30, Supporting Information). The findings suggested that LOCoF_2_ effectively suppressed the in vitro self‐renewal capacity of H22 cells. Collectively, the results demonstrated that LOCoF_2_ was a highly efficient, self‐reinforcing nanoreactor that regulated lactate metabolism and produced ROS through a cascade mechanism (Figure 3N), thereby efficiently inhibiting H22 cell stemness expression and eliminating tumor cells.

### 2.4 LOCoF_2_ Induced Pyroptosis and Cascaded to Activate the cGAS‐STING Pathway

**Figure 4:**
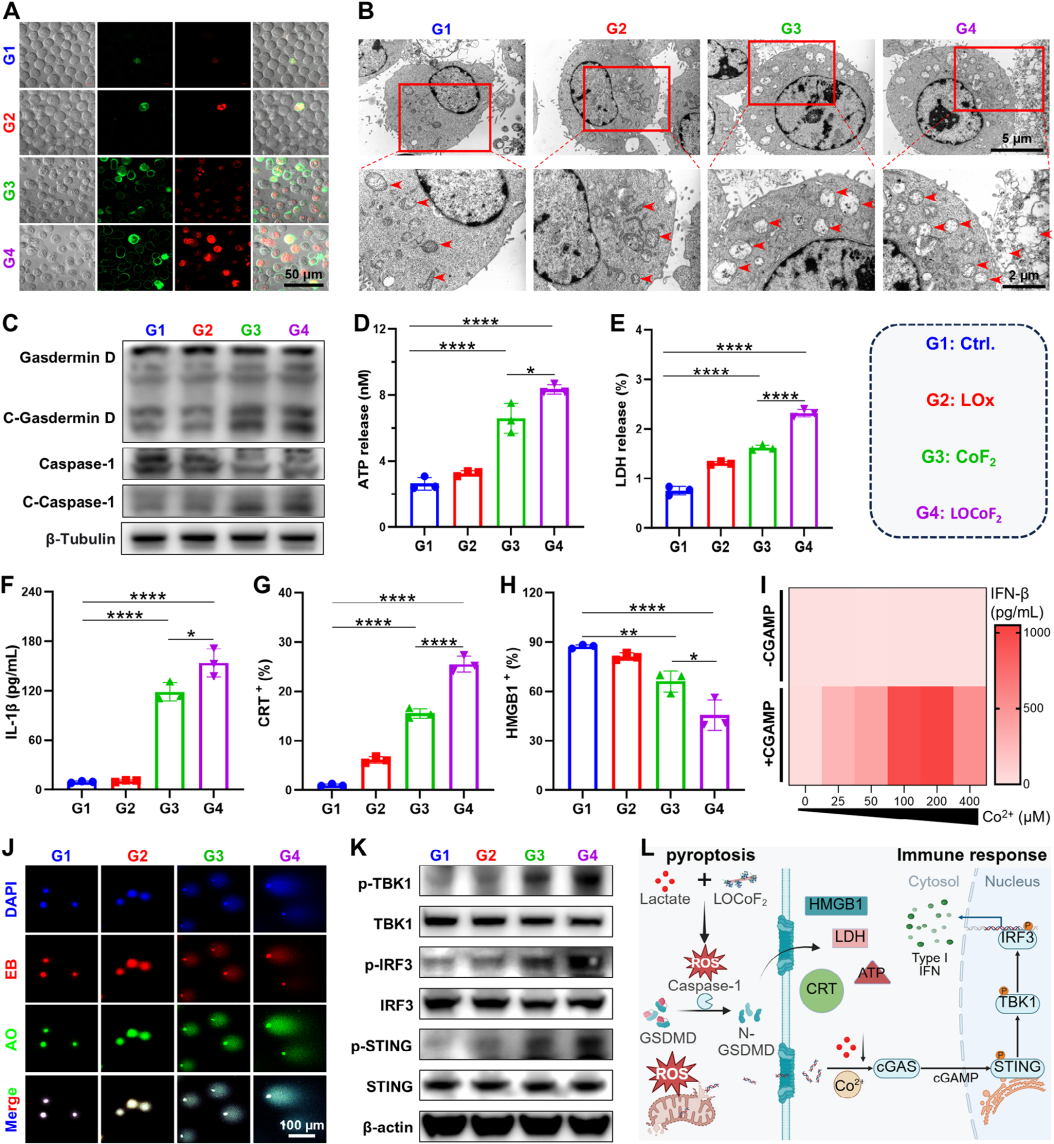
LOCoF_2_ induced pyroptosis and cascaded to activate the cGAS‐STING pathway. A) Confocal fluorescence images showing the pyroptosis in H22 cells. G1: Ctrl., G2: LOx, G3: CoF_2_, G4: LOCoF_2_. B) Biological TEM images showing the mitochondria of H22 cells. C) The expression levels of Gasdermin D, Caspase‐1, C‐Gasdermin D, and C‐Caspase‐1 in H22 cells were detected via western blotting analysis. D) ATP release levels in H22 cells. E) LDH release levels in H22 cells. F) Secretion levels of IL‐1β from H22 cells after different treatments detected by ELISA. G) quantification of CRT expression in H22 cells. H) quantification of HMGB1 release in H22 cells. I) The concentration of IFN‐β in the supernatant of BMDCs following various treatments was quantified using ELISA. J) Comet assay images showing DNA damage in H22 cells after different treatments. EB: Ethidium Bromide, AO: Acridine Orange. K) The expression levels of p‐TBK1, TBK1, p‐IRF3, IRF3, p‐STING, and STING in H22 cells were detected via WB analysis. L) The diagram illustrates that LOCoF_2_ induces tumor cell pyroptosis by consuming lactate to produce ROS and cascade to activate the STING pathway. The data represent mean  ±  SD, *n* = 3 biologically independent samples in (D–I). ANOVA was used for multiple comparisons among three or more groups. ^*^ *p* < 0.05, ^**^ *p* < 0.01, ^****^ *p* < 0.0001.

Given the potential for ROS overproduction in the cellular environment and the ability of LOCoF_2_ to jointly induce acute inflammation and pyroptosis, we conducted a more detailed analysis of the modes of cell death. First, the morphological changes in H22 cells were evaluated via annexin V‐FITC/PI co‐staining to investigate potential membrane disruption and loss of integrity associated with pyroptosis. Phospholipids on the cell membrane can bind to annexin V, while PI penetrates the compromised membrane to facilitate nuclear staining. A significant number of protruding bubbles, which are distinctive morphological features indicative of pyroptosis, were observed on the surface of LOCoF_2_‐treated cells via confocal microscopy (**Figure** **4**A). Biological‐TEM further revealed that LOCoF_2_ treatment induced substantial structural damage, characterized by mitochondrial swelling and cavitation, which was also concordant with the characteristics of pyroptosis (Figure 4B). Moreover, the expression of pyroptosis‐associated proteins in LOCoF_2_‐treated cells was assessed. Western blot analysis demonstrated marked upregulation of the expression of cleaved‐Caspase‐1 (C‐Caspase‐1) and cleaved‐ Gasdermin ‐D (C‐Gasdermin D), key proteins involved in the classical pathway of pyroptosis, following LOCoF_2_ treatment (Figure 4C; Figures S31,S32, Supporting Information). Specifically, C‐caspase‐1 cleaves gasdermin family proteins, generating N‐terminal fragments that oligomerize into plasma membrane pores, thereby facilitating cytoplasmic content release and pyroptosis. Consequently, H22 cells treated with LOCoF_2_ exhibited markedly increased ATP and LDH release compared with CoF_2_‐treated cells (Figure 4D,E). In addition, LOCoF_2_‐triggered pyroptosis significantly enhanced the secretion of the inflammatory cytokine interleukin (IL)‐1β (Figure 4F). These findings confirmed that LOCoF_2_ effectively induced significant pyroptosis through a cascade of lactate depletion and ROS generation. The intracellular contents released during pyroptosis possess immunomodulatory properties and can elicit the activation of the immune system, resulting in an inflammatory response.^[^ ^27^ ^]^ This form of immunogenic cell death (ICD) can potentiate immune responses and has significant implications for tumor regulation. To further substantiate the ICD effect mediated by pyroptosis, we analyzed the effects of exposure to calreticulin (CRT) and the release of high‐mobility group box 1 (HMGB1) following various treatments. Flow cytometric analysis revealed a marked upregulation of CRT expression in H22 cells following LOCoF_2_ treatment, with levels rising from 1.08% to 24.1%. (Figure 4G; Figure S33, Supporting Information). In addition, HMGB1 release in LOCoF_2_‐treated cells was limited to just 39.6%, a value markedly reduced compared to the other experimental groups (Figure 4H; Figure S34, Supporting Information). Therefore, LOCoF_2_ treatment significantly promoted the generation of “eat me” signal, potentially enhancing the immune response through induced pyroptosis.

Pyroptosis‐induced DNA damage results in the upregulation of γ‐H2AX. Compared with those in the control group, both CoF_2_ and LOCoF_2_ resulted in substantial increases in γH2AX levels (Figure S35, Supporting Information). Considering that Co^2+^ enhanced STING activity in a cGAMP‐dependent manner (Figure 4I), LOCoF_2_ can act as a STING agonist, selectively promoting the activation of the cGAS‐STING signaling cascade.^[^ ^11a,d^ ^]^ Furthermore, the comet assay revealed that the tail moment of LOCoF_2_‐treated cells was significantly longer than that of both control and CoF_2_‐treated cells at the 6 h time point, suggesting that LOCoF_2_ treatment induced more severe DNA damage (Figure 4J; Figure S36, Supporting Information). Pyroptosis leads to the extracellular release of substantial amounts of mtDNA, which is a potent activator of the cGAS‐STING pathway. Western blot analysis revealed that treatment with CoF_2_ and LOCoF_2_ led to a significant increase in the phosphorylation levels of TBK1, IRF3, and STING proteins (p‐TBK1, p‐IRF3, and p‐STING). Notably, the LOCoF_2_ group exhibited higher phosphorylation levels than did the CoF_2_ group (Figure 4K; Figures S37,S38, Supporting Information). The evidence presented underscored the critical role of LOCoF_2_ in stimulating the cGAS‐STING pathway. In summary, the specific mechanism by which LOCoF_2_ induced cell pyroptosis and ICD, caused DNA damage, and subsequently synergistically activated the cGAS‐STING signaling pathway was as follows (Figure 4L): LOCoF_2_ initially increased ROS generation through an intracellular lactate cascade, which subsequently activated caspase 1 to cleave GSDMD into its N‐terminal fragments. These fragments were translocated to the cell membrane to form pores that facilitated the release of cellular contents, thereby driving pyroptosis and inducing DNA damage in tumor cells via the ROS pathway. Concurrently, the released cellular contents, particularly mtDNA, were recognized as signaling molecules by cGAS, leading to the activation and amplification of the cGAS‐STING pathway. Therefore, LOCoF_2_ modulated lactate metabolism and activated the pyroptosis‐STING signaling pathway, thereby reversing the immunosuppressive TME and enhancing the immune response, thus improving the efficacy of tumor therapy.

### 2.5 In vivo Antitumor Therapy and Immune Evaluation

**Figure 5:**
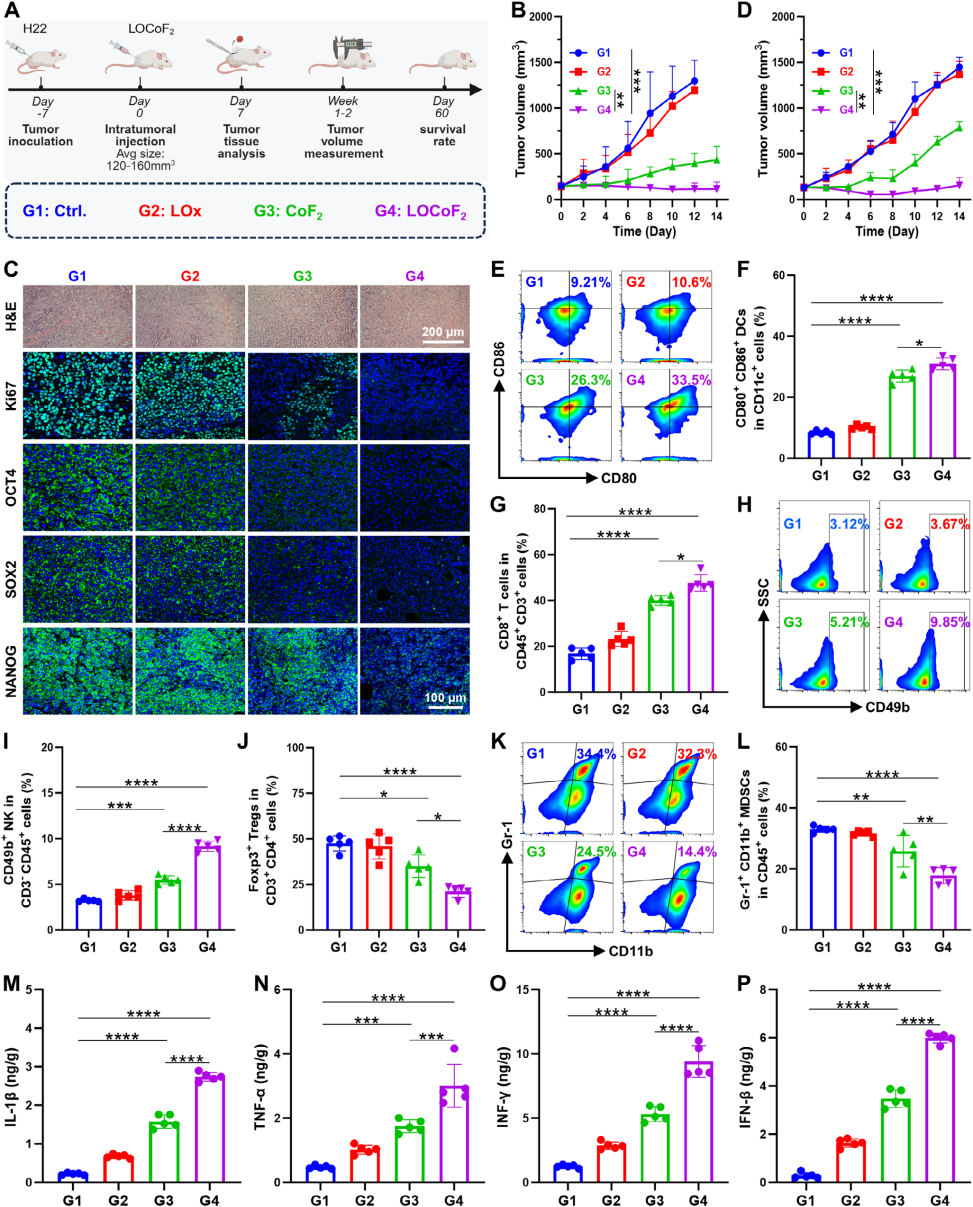
LOCoF_2_‐mediated tumor growth suppression and immune microenvironment remodeling. A) Schematic of the murine treatment protocol. B) Changes in the growth curve of H22 subcutaneous tumor after various treatments. G1: Ctrl., G2: LOx, G3: CoF_2_, G4: LOCoF_2_. C) Images of tumor sections after H&E, Ki67, OCT4, SOX2 and NANOG staining obtained from mice subjected to different treatments. D) Changes in the growth curve of HuH7 subcutaneous tumor after various treatments. E) Flow cytometry analysis of DCs maturation (CD11c^+^CD80^+^CD86^+^) within lymph node populations across treatment groups and F) statistical data. G) Statistical data of CD45^+^CD3^+^CD8^+^ T cells after various treatments. H) Flow cytometry analysis of CD45^+^CD3^−^CD49b^+^ cells after various treatments and I) statistical data. J) Statistical data of Foxp3^+^ CD4^+^ Tregs among CD3^+^ CD45^+^ *T* cells after various treatments. K) Flow cytometry analysis of MDSC (CD45^+^Gr‐1^+^CD11b^+^) cells and L) statistical data. M‐P) Concentrations of IL‐1β M), TNF‐α N), IFN‐γ O), and INF‐β P) in the supernatant after various treatments. The data represent mean  ±  SD, *n* = 6 biologically independent samples in (B, C), *n* = 5 biologically independent samples in (E–P). ANOVA was used for multiple comparisons among three or more groups. ^*^ *p* < 0.05, ^**^ *p* < 0.01, ^***^ *p* < 0.001, ^****^ *p* < 0.0001.

Encouraged by the significant in vitro killing effect of LOCoF_2_ on tumor cells, we further evaluated the in vivo antitumor effects of LOCoF_2_. When the tumor volume reached ≈120 mm^3^, H22 subcutaneous tumor‐bearing mice were randomized into four experimental groups: 1) control, 2) LOx, 3) CoF_2_, and 4) LOCoF_2_. The tumor status was monitored and recorded every other day (**Figure** **5**A). Analysis of tumor volume revealed that LOx alone had a moderate inhibitory effect compared with that in the control group. Both CoF_2_ and LOCoF_2_ demonstrated significant antitumor activity, with LOCoF_2_ showing particularly pronounced efficacy (Figure 5B; Figure S39, Supporting Information). These findings show the potential of LOCoF_2_ nanoreactors in regulating tumor lactate metabolism to improve cancer therapy. Notably, the LOCoF_2_ group exhibited significantly prolonged survival compared with the other treatment groups (Figure S40, Supporting Information). Specifically, five mice exhibited complete tumor regression with sustained remission after 60 days. In addition, no significant deviation in body weight fluctuation was observed among the treatment groups during the treatment period (Figure S41, Supporting Information). Furthermore, histological analysis of major organs in mice treated with LOCoF_2_ via hematoxylin and eosin (H&E) staining at various treatment intervals revealed no significant tissue damage or inflammatory response, demonstrating the outstanding biocompatibility of the LOCoF_2_ based approach (Figure S42, Supporting Information). Consistently, serum biochemical parameters in the LOCoF_2_ treated cohort remained within physiological limits and were comparable to those observed in the control group, suggesting the absence of notable systemic toxicity (Figure S43, Supporting Information). To further elucidate the mechanism of LOCoF_2_ treatment, tumor sections were subjected to H&E staining to assess treatment‐induced cellular damage. Notably, significant shrinkage and a reduced number of nuclei were observed in the LOCoF_2_ group, indicating severe damage. Additionally, Ki‐67 immunofluorescence staining revealed a substantial decrease in cell proliferation in the LOCoF_2_ group, suggesting a pronounced inhibitory effect on tumor growth. Immunofluorescence staining for OCT4, SOX2, and NANOG, which are markers of tumor stemness, revealed significant decreases in the expression levels of these genes in the LOCoF_2_ group, corroborating the above In Vitro findings and indicating the potent suppression of tumor recurrence and proliferation (Figure 5C). Furthermore, it is noteworthy that LOCoF_2_ demonstrated considerable therapeutic efficacy in the humanized HuH7 xenograft nude mouse model (Figure 5D; Figures S44,S45, Supporting Information). Although the therapeutic outcome was not as pronounced as in the H22 tumor model, this may be attributed to the compromised immune system of nude mice. Collectively, these results demonstrated that LOCoF_2_ not only significantly reduced the tumor burden but also markedly extended the survival of the mice, suggesting a promising therapeutic strategy based on LOCoF_2_ for solid tumors.

Ideally, the goal of tumor therapy is not only to eliminate local tumors but also to activate the systemic immune system, thereby reversing the immunosuppressive TME and ultimately inhibiting the metastasis and recurrence of tumor cells.^[^ ^28^ ^]^ Consequently, further investigations into antitumor immunity were undertaken to elucidate the mechanisms underlying the superior therapeutic efficacy of LOCoF_2_. After a 7‐day treatment period, tumor tissues and draining lymph nodes were harvested and homogenized for comprehensive immunological analysis. The maturation status of dendritic cells (DCs) in these lymph nodes was evaluated by flow cytometry. In comparison to alternative interventions, CoF_2_ and LOCoF_2_ treatments demonstrated a markedly enhanced capacity to induce dendritic cell maturation, as evidenced by a substantial elevation in the proportion of mature dendritic cells from 9.21% to 33.5% within tumor tissues of mice administered LOCoF_2_ (Figure 5E,F; Figure S46, Supporting Information). These findings were important for promoting the subsequent antigen presentation process. In addition, comprehensive evaluation of T cell infiltration within the TME revealed that the proportion of CD8^+^ cytotoxic T lymphocytes increased markedly by 25.5% and 40.4% following administration of CoF_2_ and LOCoF_2_, respectively (Figure 5G; Figures S47,S48, Supporting Information). This finding underscored the robust activation of antitumor immune responses. Additionally, LOCoF_2_ markedly enhanced the infiltration of natural killer (NK) cells into the TME (Figure 5H,I; Figure S49, Supporting Information), further highlighting its potent therapeutic efficacy. Notably, we detected a 12.9% reduction in Treg populations and a marked 20% decrease in myeloid‐derived suppressor cell (MDSC) levels following LOCoF_2_ administration (Figure 5J–L; Figures S50–S52, Supporting Information). Given their potent immunosuppressive functions, the reduction in MDSCs and Tregs corroborated the effective activation of antitumor immunity and positively influenced the outcomes of immunotherapy. Furthermore, alterations in cytokine levels were quantified via enzyme‐linked immunosorbent assay (ELISA). Importantly, IL‐1β, a pivotal proinflammatory cytokine involved in the canonical pyroptosis signaling cascade, exhibited a marked elevation in the LOCoF_2_‐treated cohort (Figure 5M). In addition, this therapeutic intervention elicited the release of various cytotoxic cytokines, including tumor necrosis factor‐alpha (TNF‐α), interferon‐gamma (IFN‐γ), and IFN‐β (Figure 5N–P). Overall, LOCoF_2_ promoted immune cell infiltration and upregulated proinflammatory cytokines by consuming the lactate‐mediated pyroptosis cascade in tumors, thereby facilitating an effective antitumor immune response.

### 2.6 RNA Sequencing and Bilateral Tumors Combined With α‐PD‐1 Therapy

**Figure 6:**
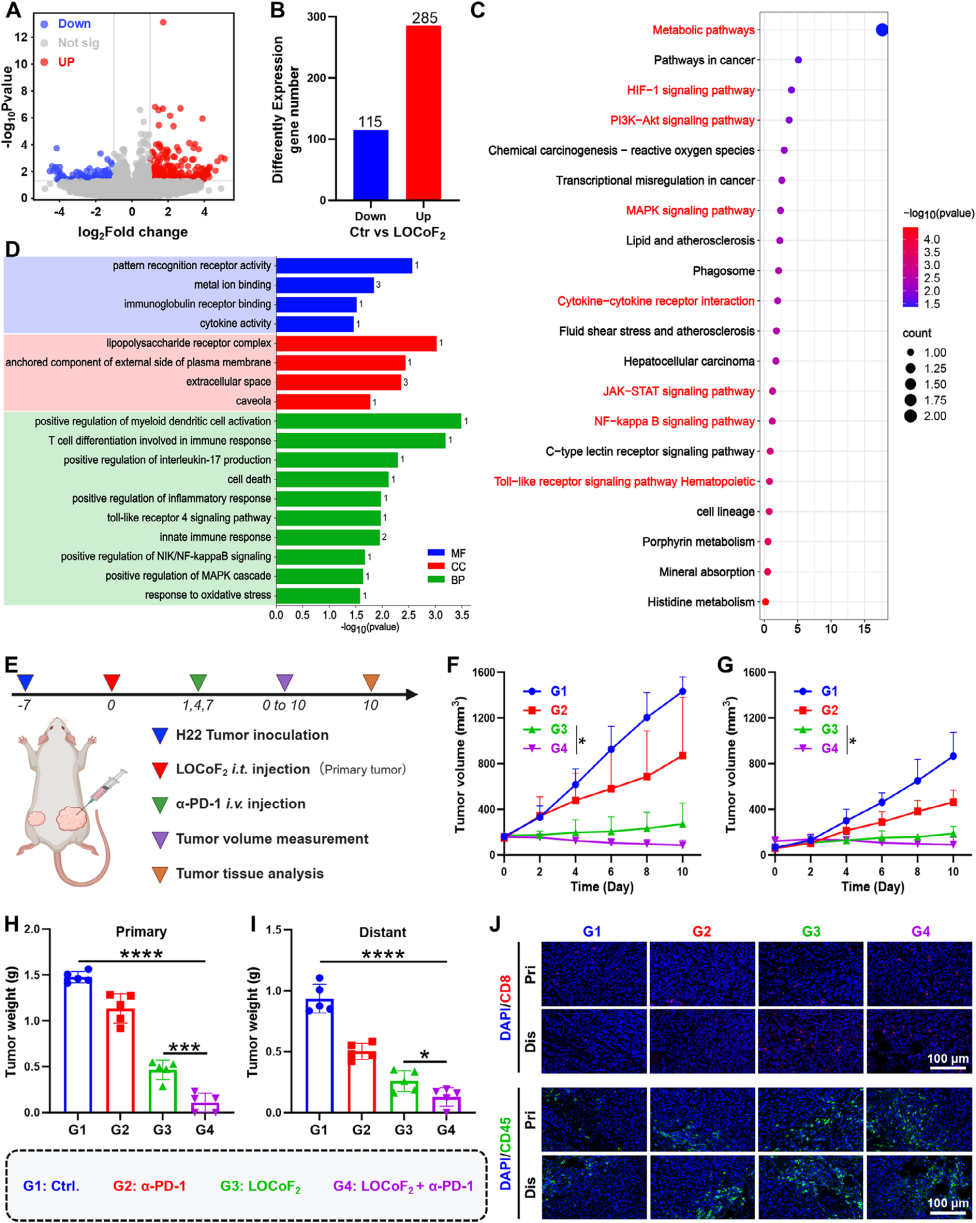
RNA‐sequencing analysis and combined α‐PD‐1 immunotherapy. A) Volcano plots and B) statistical data of the downregulated genes and upregulated genes of LOCoF_2_ compared with those of the control group. C) Results of the KEGG pathway enrichment analysis of differentially expressed genes between cells treated with LOCoF_2_ and those treated with PBS. D) GO and KEGG analyses of the differentially expressed genes in the LOCoF_2_ group and control group (BP, biological process; CC, cellular components; MF, molecular function). E) Schematic illustration of the In vivo treatment procedure in mice. F) Changes in the volume of primary and G) distant H22 tumors after different treatments. G1: Ctrl., G2: α‐PD‐1, G3: LOCoF_2_, G4: LOCoF_2_ + α‐PD‐1. H) Primary and I) distant tumor weights after different treatments. J) Fluorescence staining of H22 liver tumors from various groups for CD45 and CD8. The data represent mean  ±  SD, *n* = 5 biologically independent samples in (F‐I). ANOVA was used for multiple comparisons among three or more groups. ^*^ *p* < 0.05, ^***^ *p* < 0.001, ^****^ *p* < 0.0001.

To further elucidate the potential therapeutic mechanism of LOCoF_2_ nanoreactors in HCC, we conducted a transcriptomic analysis to evaluate mRNA variations in H22 tumor tissues both with and without treatment. Unsupervised hierarchical clustering revealed that samples within the same treatment group were clearly clustered, indicating high reliability of the RNA sequencing data (Figure S53, Supporting Information). A total of 400 differentially expressed genes (DEGs) were identified between the LOCoF_2_‐treated group and the control group, with 115 genes downregulated and 285 genes upregulated (**Figure** **6**A,B; Figure S54, Supporting Information). Following the acquisition of diverse RNA sequencing results, gene ontology (GO) and Kyoto Encyclopedia of Genes and Genomes (KEGG) analyses were conducted to elucidate the alterations in mRNA biological functions and the associated pathways (Figure 6C). The analysis revealed that genes that were differentially expressed following LOCoF_2_ treatment were significantly enriched in inflammatory signaling pathways, including the NF‐κB signaling pathway, cytokine‐cytokine receptor interaction, PI3K‐Akt signaling pathway, JAK‐STAT signaling pathway, and Toll‐like receptor signaling pathway. These findings underscored the intimate association between LOCoF_2_‐induced lactate depletion in tumor tissues and oxidative stress‐mediated pyroptosis‐induced inflammation (Figure 6D). Notably, metabolic pathways and HIF‐1 signaling pathways were also enriched, suggesting potential changes in energy metabolism and oxygen levels within tumor tissues post‐treatment.

Despite the transformative impact of immunotherapy involving ICB therapy for cancer treatment, its clinical efficacy remains constrained by ICB resistance.^[^ ^29^ ^]^ Leveraging pyroptosis as a potential strategy to increase immune system activation is anticipated to improve the effectiveness of immunotherapy. Motivated by the superior performance of LOCoF_2_, the In vivo systemic antitumor effect in combination with α‐PD‐1 therapy was investigated. Mice bearing bilateral H22 tumors were divided into four groups: 1) control, 2) α‐PD‐1, 3) LOCoF_2_, and 4) LOCoF_2_ + α‐PD‐1. In these groups, the right tumor served as the primary tumor (receiving treatment), while the left tumor remained untreated to serve as a distant tumor. LOCoF_2_ was administered intratumorally to the primary tumors of groups 3 and 4 on day 0. For groups 2 and 4, α‐PD‐1 was administered intravenously (i.v.) on days 1, 4, and 7 (Figure 6E). As anticipated, the combination of LOCoF_2_ and α‐PD‐1 antibody was the most effective therapeutic outcome for primary tumors, with ≈40% of tumors being completely eradicated. These findings indicated that the combined treatment strategy had a substantial therapeutic advantage (Figure 6F–H; Figures S55,S56, Supporting Information). Moreover, the administration of LOCoF_2_ to primary tumors not only reduced the growth of distant tumors but also significantly enhanced the inhibitory effect on distant tumors when synergistically combined with α‐PD‐1 therapy (Figure 6G,I; Figures S57,S58, Supporting Information). In addition, no significant deviation in body weight fluctuation was observed throughout the treatment duration (Figure S59, Supporting Information). Moreover, immunofluorescence‐stained images of H22 tumors were obtained to elucidate the underlying mechanisms driving this synergistic antitumor effect (Figure 6J). The LOCoF_2_ + α‐PD‐1 combination therapy group markedly increased infiltration of CD8^+^ T cells and CD45^+^ immune cells, thereby modulating the immunosuppressive TME to enhance cancer cell eradication. These findings suggested that the catalytic metalloimmunotherapy strategy, which utilized the self‐cascaded pyroptosis‐STING initiator LOCoF_2_ in conjunction with ICB, effectively augmented the immune response to inhibit both primary and metastatic tumor growth.

### 2.7 LOCoF_2_‐Mediated TAE/TACE Treatment of Rat Orthotopic N1‐S1 Tumors

**Figure 7:**
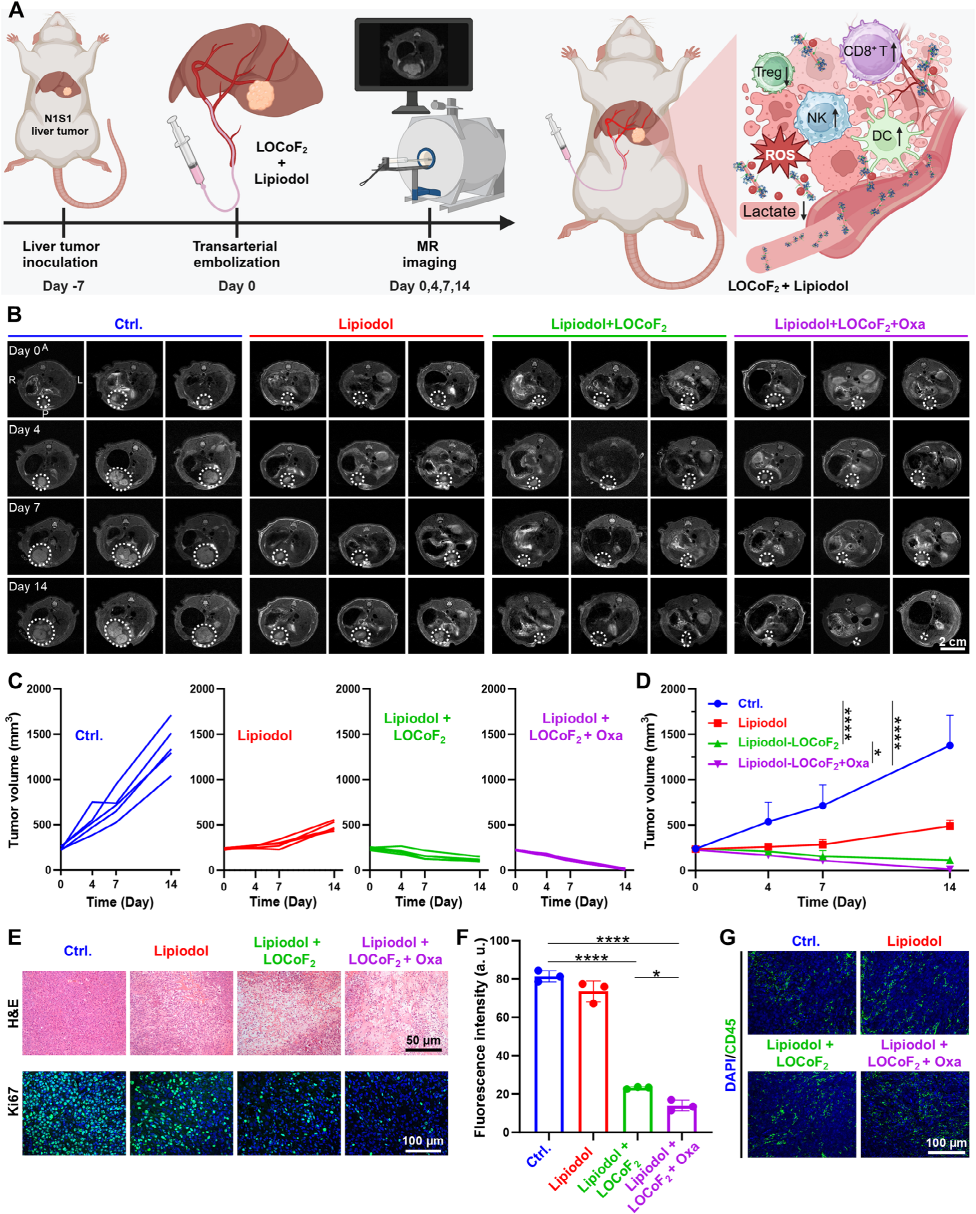
In vivo evaluation of LOCoF_2_‐mediated TACE in an orthotopic N1‐S1 hepatoma model. A) Experimental timeline for TAE/TACE intervention in tumor‐bearing rats. B) T2‐weighted contrast‐enhanced MR image of N1‐S1 tumors post‐treatment. L: Left, R: Right, A: Anterior, P: Posterior. C) Individual growth curves of N1‐S1 tumors. D) Changes in the tumor growth curves. E) Representative images of H&E and Ki67 staining of mouse tumor sections. F) The fluorescence intensity of ki67 after various treatments. The data are presented as the mean values ± SD. G) Fluorescence staining of N1‐S1 liver tumors from various groups for CD45. The data represent mean  ±  SD, *n* = 5 biologically independent samples in (C, D). ANOVA was used for multiple comparisons among three or more groups. ^*^ *p* < 0.05, ^**^ *p* < 0.01, ^****^ *p* < 0.0001.

TAE and TACE are established as first‐line treatments for unresectable HCC.^[^ ^2a^ ^]^ In TACE therapy, embolic agents such as Lipiodol or polymer beads are combined with chemotherapeutic agents and administered directly into liver tumors via the hepatic artery.^[^ ^2^, ^30^ ^]^ Nevertheless, this strategy continues to encounter challenges due to the immunosuppressive nature of the TME, which substantially limits therapeutic efficacy. Given that the developed LOCoF_2_ demonstrated remarkable advantages in modulating the immunosuppressive TME in mice during synergistic immunotherapy, we subsequently dispersed LOCoF_2_ in Lipiodol to achieve synergistic TAE/TACE therapy. The experiment utilized a rat orthotopic N1‐S1 liver cancer model in which N1‐S1 cells were injected directly into the left lobe of the liver. Seven days post‐inoculation, the rats were subjected to the following treatments: Group I: control, Group II: TAE with Lipiodol; Group III: TAE with Lipiodol + LOCoF_2_; and Group IV: TACE with Lipiodol + LOCoF_2_ + Oxa (**Figure** **7**A). Tumor volume images acquired via a 7.0 T small animal magnetic resonance (MR) imaging system on days 0, 4, 7, and 14 post‐treatment demonstrated that the growth of N1‐S1 tumors was partially inhibited following bare Lipiodol TAE compared with that of untreated controls. Notably, the tumor inhibition effect of Lipiodol + LOCoF_2_ treatment was more significant, and the tumor inhibition effect was greatly enhanced after Lipiodol + LOCoF_2_ + Oxa treatment (Figure 7B–D). In addition, orthotopic rat liver tissue sections were subjected to staining on day 7. H&E and Ki67 staining of tumor tissue sections revealed that the treatments involving Lipiodol + LOCoF_2_, with or without Oxa, induced the most substantial tumor cell damage and the lowest proliferation rates (Figure 7E,F). Further immunofluorescence staining analysis revealed significantly increased infiltration of CD45^+^ immune cells in tumors treated with Lipiodol + LOCoF_2_ or Lipiodol + LOCoF_2_ + Oxa, thereby modulating the immunosuppressive TME to enhance cancer cell eradication (Figure 7G). Therefore, these results indicated that LOCoF_2_ could be used for TAE/TACE therapy for liver cancer in addition to direct intratumoral injection, providing a viable and practical option for clinical application.

## 3 Conclusion

In summary, we developed an innovative lactate‐depleting nanoreactor, LOCoF_2_, designed to mediate self‐reinforced pyroptosis and amplify cGAS‐STING signaling cascades, thereby enhancing catalytic immunotherapy. The LOCoF_2_ nanoreactor operates through a dual mechanism: initially, it acts as a pyroptosis activator by catalyzing ROS production, inducing pyroptosis in cancer cells while simultaneously inhibiting damage repair pathways. Subsequently, it functions as an intelligent STING agonist, selectively detecting pyroptosis‐released mtDNA and augmenting cGAS‐STING pathway activation. When administered to tumors, LOCoF_2_ elicited a robust immune response, significantly enhancing antitumor efficacy. Notably, combining LOCoF_2_ with ICB resulted in potent suppression of both primary and metastatic tumors.

Local delivery of the LOCoF_2_ nanoreactor, a potent immune‐stimulating agent, has emerged as the most viable strategy for eliciting a robust antitumor immune response while minimizing the risk of systemic immune activation and associated cytokine storms. Furthermore, we demonstrated that LOCoF_2_ can enhance TAE/TACE therapy, a first‐line treatment for unresectable HCC. By leveraging the metabolic properties of tumors, LOCoF_2_ transforms lactate, a key immunosuppressive metabolite, into a therapeutic asset through a series of catalytic reactions. This approach enhances pyroptosis‐STING coordination, achieving superior tumor control and offering novel insights into tumor therapy. The present study not only underscores the therapeutic potential of metabolic reprogramming in immunotherapy but also provides a blueprint for designing nano‐catalytic systems that exploit tumor‐specific vulnerabilities. The successful application of LOCoF_2_ in TAE/TACE therapy further validates its clinical feasibility, paving the way for next‐generation interventional immunotherapies for advanced malignancies.

## 4 Experimental Section

### Statistical Analysis

The experimental results are expressed as mean ± SD and analyzed by GraphPad Prism 8 software. The *P* values indicated in the figure were determined by employing the two‐tailed student's *t*‐test, and one‐way analysis of variance (ANOVA) was used for multiple comparisons among three or more groups. ^*^ *p* < 0.05, ^**^ *p* < 0.01, ^***^ *p* < 0.001, ^****^ *p* < 0.0001.

### Ethics Approval and Consent to Participate

All animal experiments were carried out following protocols approved by Soochow University Laboratory Animal Center (202409A0029).

## Conflict of Interest

The authors declare no conflict of interest.

## Author Contributions

L.Z., S.S., and D.W. contributed equally to this work. L.C. oversaw all research; N.Y., L.Z., H.Z., and L.C. designed the experiments; L.Z., S.S., D.W., D.W., J.N., Z.P., J.Q., L.Z., and N.Y. performed the experiments; all authors analyzed and interpreted the data; L.Z., N.Y., L.Z., H.Z., and L.C. wrote and revised the manuscript; all authors reviewed and edited the paper.

## Supporting information



Supporting Information

## Data Availability

The data that support the findings of this study are available from the corresponding author upon reasonable request.

## References

[1] R. L. Siegel , A. N. Giaquinto , A. Jemal , Ca‐Cancer J. Clin. 2024, 74, 12. 38230766 10.3322/caac.21820

[2] a) H.‐D. Zhu , H.‐L. Li , M.‐S. Huang , W.‐Z. Yang , G.‐W. Yin , B.‐Y. Zhong , J.‐H. Sun , Z.‐C. Jin , J.‐J. Chen , N.‐J. Ge , Signal Transduct. Target. Ther. 2023, 8, 58; 36750721 10.1038/s41392-022-01235-0 PMC9905571

[3] H.‐D. Zhu , R. Liu , Z.‐Z. Jia , D.‐D. Xia , B.‐Y. Zhong , W.‐Z. Fan , J. Lu , M. Zhao , G.‐J. Teng , Eng. Med. 2024, 42, 1830.

[4] a) J. L. Raoul , A. Forner , L. Bolondi , T. T. Cheung , R. Kloeckner , T. de Baere , Cancer Treat. Rev. 2019, 72, 28; 30447470 10.1016/j.ctrv.2018.11.002

[5] a) F. Deschamps , T. Isoardo , S. Denis , N. Tsapis , L. Tselikas , V. Nicolas , A. Paci , E. Fattal , T. de Baere , N. Huang , L. Moine , Acta Biomater. 2019, 87, 177; 30708065 10.1016/j.actbio.2019.01.054

[6] a) L. H. Butterfield , Y. G. Najjar , Nat. Rev. Immunol. 2024, 24, 399; 38057451 10.1038/s41577-023-00973-8 PMC11460566

[7] F. G. Dall'Olio , A. Marabelle , C. Caramella , C. Garcia , M. Aldea , N. Chaput , C. Robert , B. Besse , Nat. Rev. Clin. Oncol. 2022, 19, 75. 34642484 10.1038/s41571-021-00564-3

[8] a) H. Zhu , C. Yang , A. Yan , W. Qiang , R. Ruan , K. Ma , Y. Guan , J. Li , Q. Yu , H. Zheng , View 2023, 4, 20220067;

[9] a) N. Samson , A. Ablasser , Nat. Cancer 2022, 3, 1452; 36510011 10.1038/s43018-022-00468-w

[10] a) X. Sun , X. Zhou , X. Shi , O. A. Abed , X. An , Y. L. Lei , J. J. Moon , Nat. Biomed. Eng. 2024, 8, 1073; 38914800 10.1038/s41551-024-01221-7 PMC11410547

[11] a) Q. Yu , S. Sun , N. Yang , Z. Pei , Y. Chen , J. Nie , H. Lei , L. Wang , F. Gong , L. Cheng , J. Am. Chem. Soc. 2025, 147, 3161; 39818788 10.1021/jacs.4c12552

[12] a) P. Apostolova , E. L. Pearce , Trends Immunol. 2022, 43, 969; 36319537 10.1016/j.it.2022.10.005 PMC10905416

[13] H. Li , C. Liu , R. Li , L. Zhou , Y. Ran , Q. Yang , H. Huang , H. Lu , H. Song , B. Yang , Nature 2024, 634, 1229. 39322678 10.1038/s41586-024-07992-y

[14] H. Chen , Y. Li , H. Li , X. Chen , H. Fu , D. Mao , W. Chen , L. Lan , C. Wang , K. Hu , Nature 2024, 631, 663. 38961290 10.1038/s41586-024-07620-9 PMC11254748

[15] a) N. T. Nguyen , S. Gevers , R. N. Kok , L. M. Burgering , H. Neikes , N. Akkerman , M. A. Betjes , M. C. Ludikhuize , C. Gulersonmez , E. C. Stigter , Cell Metab. 2025, 37, 903; 39933514 10.1016/j.cmet.2025.01.002

[16] a) Y. Liu , R. Pan , Y. Ouyang , W. Gu , T. Xiao , H. Yang , L. Tang , H. Wang , B. Xiang , P. Chen , Signal Transduct. Target. Ther. 2024, 9, 245; 39300122 10.1038/s41392-024-01958-2 PMC11413206

[17] a) P. Zheng , G. Wang , B. Liu , H. Ding , B. Ding , J. Lin , J. Am. Chem. Soc. 2025, 145, 6007.; 10.1021/jacs.4c09566 PMC11744746 39743855

[18] a) R. Miao , C. Jiang , W. Y. Chang , H. Zhang , J. An , F. Ho , P. Chen , H. Zhang , C. Junqueira , D. Amgalan , Immunity 2023, 56, 2523; 37924812 10.1016/j.immuni.2023.10.004 PMC10872579

[19] a) J. Nie , N. Yang , S. Sun , L. Wang , Z. Pei , J. Wu , Q. Yu , Z. Han , Y. Chen , L. Cheng , Angew. Chem., Int. Ed. 2025, 64, 202416426; 10.1002/anie.202416426 39305135

[20] a) X. Ma , B. Ding , Z. Yang , S. Liu , Z. Liu , Q. Meng , H. Chen , J. Li , Z. Li , P. a. Ma , J. Am. Chem. Soc. 2024, 146, 21496; 39073804 10.1021/jacs.4c04385

[21] a) S. Cai , J. Liu , J. Ding , Z. Fu , H. Li , Y. Xiong , Z. Lian , R. Yang , C. Chen , Angew. Chem., Int. Ed. 2022, 134, 202204502; 10.1002/anie.202204502 35972794

[22] a) D. Jana , Y. Zhao , Exploration 2022, 2, 20210238; 37323881 10.1002/EXP.20210238 PMC10191001

[23] a) S. Sun , N. Yang , Z. Pei , F. Gong , L. Cheng , Coord. Chem. Rev. 2025, 530, 216494;

[24] S. Zhao , H. Li , R. Liu , N. Tao , L. Deng , Q. Xu , J. Hou , J. Sheng , J. Zheng , L. Wang , J. Am. Chem. Soc. 2023, 145, 10322. 37097216 10.1021/jacs.3c02005

[25] H. Liu , S. Jiang , M. Li , S. Lei , J. Wu , T. He , D. Wang , J. Lin , P. Huang , ACS Nano 2024, 18, 30345. 39432819 10.1021/acsnano.4c07374

[26] a) H. Yang , Q. Wang , Z. Li , F. Li , D. Wu , M. Fan , A. Zheng , B. Huang , L. Gan , Y. Zhao , Nano Lett. 2018, 18, 7909; 30450914 10.1021/acs.nanolett.8b03828

[27] a) G. Zhu , Y. Xie , J. Wang , M. Wang , Y. Qian , Q. Sun , Y. Dai , C. Li , Adv. Mater. 2024, 36, 2409066; 10.1002/adma.202409066 39285820

[28] N. K. Wolf , D. U. Kissiov , D. H. Raulet , Nat. Rev. Immunol. 2023, 23, 90. 35637393 10.1038/s41577-022-00732-1

[29] a) A. J. Korman , S. C. Garrett‐Thomson , N. Lonberg , Nat. Rev. Drug Discovery 2022, 21, 509; 34937915 10.1038/s41573-021-00345-8

[30] D. Wang , L. Zhang , W. H. Yang , L. Z. Zhang , C. Yu , J. Qin , L. Z. Feng , Z. Liu , G. J. Teng , Adv. Sci. 2025, 12, 2410484. 10.1002/advs.202410484 PMC11809372 39680010

