# Supplementary material for: Lactate‐Modulating Nanoreactors Facilitate Self‐Amplifying Pyroptosis and the cGAS‐STING Cascade for Potentiated Catalytic‐Immunotherapy
Source: Adv Sci (Weinh). 2025 Aug 28;12(43):e11144. doi: 10.1002/advs.202511144 (PMC12631825; doi:10.1002/advs.202511144)
Supplement: Supplementary file 1 — Supporting Information [file ADVS-12-e11144-s001.docx]

**Supporting** **Information**

**Lactate-Modulating Nanoreactors Facilitate Self-Amplifying Pyroptosis and the cGAS-STING Cascade for Potentiated** **Catalytic-Immunotherapy**

*Linzhu Zhang^[a,b]#^, Shumin Sun^[b]#^, Duo Wang^[a]#^, Nailin Yang^[b]^*, Di Wang^[b]^, Jihu Nie^[b]^, Zifan Pei^[b]^, Juan Qin^[a]^, Lei Zhang^[a]^*, Haidong Zhu^[a]^*, and Liang Cheng^[b]^**

[a] Mr. L. Zhang, Dr. D. Wang, Miss J. Qin, Dr. L. Zhang, Prof. H. Zhu

Center of Interventional Radiology & Vascular Surgery, Department of Radiology,

Nurturing Center of Jiangsu Province for State Laboratory of AI Imaging

& Interventional Radiology (Southeast University),

Basic Medicine Research and Innovation Center of Ministry of Education,

Zhongda Hospital, Medical School, Southeast University,

87 Dingjiaqiao Road, Nanjing, 210009, China

Emails: [zhang_lei@seu.edu.cn](mailto:zhang_lei@seu.edu.cn); zhuhaidong@seu.edu.cn;

[b] Mr. L. Zhang, Miss S. Sun, Dr. N. Yang, Mr. D. Wang, Mr. J, Nie, Mr. Z. Pei, Prof. L. Cheng

Institute of Functional Nano & Soft Materials (FUNSOM),

Jiangsu Key Laboratory for Carbon-Based Functional Materials & Devices,

Soochow University, Suzhou, 215123, China

Emails: [lcheng2@suda.edu.cn](mailto:lcheng2@suda.edu.cn); [nlyang@suda.edu.cn](mailto:nlyang@suda.edu.cn)

^#^These authors contributed equally to this work.

# Experimental section

## Materials

Cobalt acetylacetonate (Co(C_5_H_7_O_2_)_2_) was purchased from Shanghai Debao Biotechnology Co., Ltd. Ammonium fluoride (NH_4_F) and dibenzyl ether (95%) were purchased from Alfa Aesar (China) Chemical Co., Ltd. Dodecanol (C_12_H_26_O_2_, 93%), oleic acid (90%), and oleylamine (95%) were purchased from Beijing Anheuser-Busch InBev Science Co., Ltd. 1,3-Diphenylisobenzofuran (DPBF) was purchased from Macklin Biochemical Technology Co., Ltd. 3,3',5,5'-Tetramethylphenyldiamine (TMB) was purchased from Beijing J&K Science Co., Ltd. Hydrogen peroxide (H_2_O_2_, 30%) was purchased from Shanghai Lingfeng Chemical Reagent Co., Ltd. All the chemicals were of analytical grade and used without further purification.

An adenosine triphosphate (ATP) content determination kit (BC0300) was purchased from Beyotime Biotechnology Co., Ltd. Lactate Dehydrogenase (LDH) content assay kit (C0016) and mitochondrial membrane potential test kit (JC-1, J8030) were purchased from Beijing Solarbio Technology Co., Ltd. Lactate oxidase (LOx) was purchased from ShangHai Ponsure Biotech, Inc. DSPE-PEG_2000_-NH_2_ (F08010) was purchased from AVT (Shanghai) Pharmaceutical Tech Co., Ltd. Anti-HMGB1 rabbit mAb (ab79823), anti-CRT rabbit mAb (ab2907), Anti CD3-FITC (Biolegend, clone 17A2, Catalog: 100204), anti-CD4-APC (Biolegend, clone GK1.5, Catalog: 100412), anti-CD8-PE (Biolegend, clone 53-6.7, Catalog: 100708), anti-CD11c-FITC (Biolegend, clone N418, Catalog: 117306), anti-CD80-APC (Biolegend, clone 16-10A1, Catalog: 104714), anti-CD86-PE (Biolegend, clone GL-1, Catalog: 105008), anti-F4/80-FITC (Biolegend, 2 clone QA17A29, Catalog: 157310), anti-CD206-APC (Biolegend, clone C068C2, Catalog: 141708), anti-CD11b-PE (Biolegend, clone M1/70, Catalog: 101280), anti-CD44-PE (Biolegend, clone IM7, Catalog: 103010), anti-CD8-PerCP (Biolegend, clone 53-6.7, Catalog: 100732), anti-Foxp3-PE (Biolegend, clone MF-14, Catalog: 126404),anti-CD62L-APC (Biolegend, clone MEL-14, Catalog: 104450) were obtained from Biolegend and diluted at 1:300 for cell staining. Mouse TNF-α (LA128801S) and IFN-γ (LA128805S) ELISA kit were purchased from Nanjing Lapuda Biotechnology Co.,Ltd. (Naniing, china). Mouse IFN-β ELISA kit (Cat No. HJ169; Epizyme, Shanghai, China) was purchased from Shanghai Epizyme. ELISA kits for IL-1β (ED-20174) was purchased from Lun Changshuo Biological Technology Co., Ltd. GenScript YoungPAGE™ Precast Gels (M01002) was obtained from GenScript Biotech Corporation. anti-Phospho-Histone H2A.X (Ser139) (CST, 2577), anti-β-Catenin (Proteintech, Catalog: 51067-2-AP), NANOG (Proteintech, Catalog: 14295-1-AP), OCT4 (Proteintech, Catalog: 11263-1-AP), SOX2 (Proteintech, Catalog: 11064-1-AP),Cleaved Caspase-1 rabbit mAb (CST, 89332), anti-Caspase-1 antibody (Abcam, AB138483), cleaved gasdermin D rabbit mAb (CST, 10137), anti-GSDMD (Abcam, AB219800) and anti-TBK1/NAK (E8I3G) (CST, Catalog: 38066), anti-phospho-TBK1/NAK (Ser172) (D52C2) XP® (CST, Catalog: 5483), anti-IRF-3 (D6I4C) XP® (CST, Catalog: 11904), anti-phospho-IRF-3 (Ser396) (D6O1M) (CST, Catalog: 29047), anti-STING (D2P2F) (CST, Catalog: 13647), anti-phospho-STING (Ser366) (E9A9K) (CST, Catalog: 50907), anti-beta-Tubulin (Proteintech, Catalog: 10094-1-AP), anti-GAPDH (Proteintech, Catalog: 10494-1-AP), and FDR007 goat anti-rabbit lgG (H + R) HRP secondary antibody (Fudebio, Hangzhou, China) was obtained and FD0040 primary antibody dilution buffer at 1:1000 for Western blotting (WB) analysis. The signals were developed using FD8030 FDbio-Dura ECL (Fudebio, Hangzhou, China). RPMI-1640 medium and fetal bovine serum (FBS) were purchased from Nanjing BioChannel Biotechnology Co., Ltd., Nanjing, China. Cell culture dishes, microplates, and pipette tips were purchased from Suzhou CellPro Biotechnology Co., Ltd. The aqueous solution used in the experiment was prepared from deionized water from a Milli-Q water purification system.

## Synthesis of CoF_2_ nanoparticles (NPs)

CoF_2_ NPs were synthesized by thermal decomposition of the high-temperature oil phase. Briefly, 1 mmol of cobalt acetylacetonate, 2 mmol of NH_4_F and 2 g of 1,2-dodecanediol, 20 mL of dibenzyl ether, 2 mL of oleylamine (OE) and 2 mL of oleic acid (OA) were added into a three-necked flask in sequence and then dispersed under magnetic stirring. The mixture was heated to 120 ^o^C and stirred for 30 min under N_2_ atmosphere. Then, the mixture was heated to 280 ^o^C and stirred for 1 h, and after naturally cooling to room temperature, the CoF_2_ was washed three times with cyclohexane and ethanol.

## Synthesis of LOCoF_2_ NPs

The synthesized CoF_2_ were modified via a one-step method. 30 mg of DSPE-PEG_2000_-NH_2_ and 10 mg of CoF_2_ were dissolved in 15 mL of dichloromethane, and treated with ultrasonic waves in a water bath for 30 min, after which the dichloromethane was removed by rotary evaporation. Then, the PEGylated CoF_2_ was ultrasonically cleaned three times with deionized (DI) water and finally fixed to 10 mL. Finally, 3 mg of LOx was added, and the mixture was vortexed at room temperature for 30 min, washed three times with deionized water, and stored at 4 ^o^C for use.

## Characterization

The morphology of the samples was characterized by transmission electron microscopy (TEM, FEITF20). Elemental mapping was performed by TEM (TALOS 200X). The chemical composition and crystallography of the samples were characterized by X-ray diffraction (XRD, Panalytical Empyrean) with Cu Kα radiation (λ = 1.54056 Å). The phase analysis was examined by X-ray photoelectron spectroscopy (XPS). UV-vis-NIR absorption spectra were recorded by UV-vis-NIR spectrophotometer (PerkinElmer Lambda 750). The concentration of Co element was determined by inductively coupled plasma optical emission spectrometer (ICP-OES). Fourier transform infrared spectrometer (FT‒IR) imaging was carried out by FT-IR Spectrometer (V70 & Hyperion 1000, Bruker, Billerica, USA). Dynamic light scattering (DLS) and zeta potential of the nanoparticles were recorded using MAL VERN ZEN3690.

## Determination of LOCoF_2_ activity

In order to detect the ability of the CoF_2_ NPs to catalyze H_2_O_2_. Specifically, to detect superoxide anion (O_2_^•−^), first, 10 μL of the DPBF (1 mg/mL) probe and 20 μL of H_2_O_2_ (1 mmol/L) were dissolved in 920 μL of deionized water, and finally, 50 μL of CoF_2_ (5 mg/mL) was added to immediately record the absorption peak at 420 nm. Subsequently, the ability of CoF_2_ to produce O_2_^•−^ and was assessed via the use of NBT probe, 15 μL of NBT in dimethyl sulfoxide (DMSO, 1 mg/mL), 50 μL of H_2_O_2_ (1 mmol/L) were dissolved in 885 μL of deionized water, and finally, 50 μL of CoF_2_ (5 mg/mL) was added to immediately record the absorption peak at 260 nm.

To detect •OH, first, 5 μL of TMB probe and 20 μL of H_2_O_2_ (1 mmol/L) were dissolved in 925 μL of deionized water, and finally, 50 μL of CoF_2_ (5 mg/mL) was added to immediately record the absorption peak at 652 nm. In addition, OPD is also used to detect •OH, 20 μL of the OPD in deionized water (H_2_O, 10.8 mg/mL), 50 μL of H_2_O_2_ (1 mmol/L) were dissolved in 880 μL of deionized water, and finally, 50 μL of CoF_2_ (5 mg/mL) was added to immediately record the absorption peak at 430 nm.

To detect the ability of the LOCoF_2_ NPs to catalyze lactate, in short, to detect O_2_^•−^, first, 10 μL of DPBF (1mg/mL) probe and 10 μL of lactate (1 mmol/L) were dissolved in 930 μL of deionized water, and finally, 50 μL of CoF_2_ (5 mg/mL) was added to immediately record the absorption peak at 420 nm. Subsequently, 15 μL of NBT in dimethyl sulfoxide (DMSO, 1 mg/mL), 100 μL of lactate (1 mol/L) were dissolved in 885 μL of deionized water, and finally, 50 μL of LOCoF_2_ (5 mg/mL) was added to immediately record the absorption peak at 260 nm. To detect •OH, 5 μL of TMB probe and 10 μL lactate (1 mmol/L) were first dissolved in 935 μL of deionized water, and finally, 50 μL CoF_2_ (5 mg/mL) was added, and the absorption peak at 652 nm was immediately recorded. In addition, OPD is also used to detect •OH, 20 μL of the OPD in deionized water (H_2_O, 10.8 mg/mL), 100 μL of lactate (1 mol/L) were dissolved in 830 μL of deionized water, and finally, 50 μL of LOCoF_2_ (5 mg/mL) was added to immediately record the absorption peak at 430 nm.

## Measurement of lactate

To measure lactate levels in cells or tumor tissues, cells were lysed or tumor tissues were homogenized with lysis buffer on ice and the supernatant was obtained by centrifugation at 12,000 rpm for 10 min at 4 ^o^C. The supernatant was collected and lactate levels were measured using a L- lactate (LA) colorimetric assay kit (Elabscience: E-BC-K044-S) according to the manufacturer's instructions.

## Cellular experiments

The H22 (RRID: CVCL_H613) (MeisenCTCC-0506-Luc1) hepatoma cell line was cultured in RPMI 1640 medium supplemented with 10% FBS (JYK-FBS-301, Jin Yuan Kang Biotechnology) and 1% penicillin-streptomycin in a standard cell culture environment (37 °C, 5% CO_2_).

To study cytotoxicity and relative cell viability, a standard CCK8 (TargetMol, USA, C0005ED) assay was used for measurement. H22 cells were evenly plated in 12-well plates (10^5^ cells per well) and cultured overnight at 37 °C. After different treatments (control, LOx, CoF_2_, and LOCoF_2_), the cells were incubated at 37 °C for 12 h. Then, CCK8 solution was added, and the mixture was incubated for 1.5 h. The cell suspension was transferred to a 96-well plate. The absorbance of each well at 450 nm was measured.

To detect cellular ROS, H22 cells were incubated with 100 μM LOCoF_2_ for 6 h and then stained with DCFH-DA (10 μM) for 30 min according to the kit recommended protocol. Then, the cells were washed with PBS and detected by flow cytometry. Afterwards, the cells were stained with DAPI for 15 min. Images were acquired by confocal microscopy.

To determine the mitochondrial membrane potential, H22 cells (1 × 10^5^/well) were seeded in 6-well plates, and the cells in different treatment groups (control, LOx, CoF_2_, and LOCoF_2_) were incubated at 37 °C for 8 h. The mitochondrial membrane potential of the cells was detected via a JC-1 kit and flow cytometer (BD Accuri C6 flow cytometer).

To assess the stemness properties of H22 cells, 1 × 10^5^ H22 cells per well were plated in 6-well plates. Cells from various experimental groups (Ctrl., LOx, CoF_2_, and LOCoF_2_) were incubated at 37 °C for 24 hours, followed by staining with antibodies specific for stemness markers. Changes in stemness marker expression were subsequently analyzed using a BD Accuri C6 flow cytometer.

To assess H22 cell spheroid formation, 2 × 10^3^ cells were plated in 6-well ultra-low attachment plates (Corning) and maintained in a specialized medium comprising DMEM/F12 (Invitrogen) supplemented with 4 μg/mL insulin (Sigma-Aldrich), B27 supplement (Invitrogen), 20 ng/mL EGF (Sigma-Aldrich), and 20 ng/mL basic FGF (Invitrogen). Following a 14-day incubation period, spheroid numbers were quantified by microscopic imaging.

To determine ATP and LDH release, H22 cells (1 × 10^5^/well) were seeded in 12-well plates, and cells in different treatment groups were incubated at 37 °C for 8 h. The supernatant was collected and treated with ATP and LDH according to the instructions. The samples were analyzed by a microplate reader.

To detect CRT expression and HMGB1 release, H22 cells (1 × 10^5^/well) were seeded in 12-well plates, and the cells in different treatment groups were incubated at 37 °C for 12 h. For HMGB1, the cells were washed with PBS, fixed with 4% paraformaldehyde, and transparentized with 0.1% Triton X-100 for 10 min. After incubation with 5% FBS for 0.5 h, the cells were incubated with an anti-HMGB1 antibody for 1 h, and then with an Alexa 488-conjugated secondary antibody for 45 min. Then, the cells were washed with PBS and detected by flow cytometry. For CRT, the cells were washed with PBS and fixed with 4% paraformaldehyde. After incubation with 5% FBS for 0.5 h, the cells were incubated with an anti-CRT antibody for 1 h, and then with an Alexa 488-conjugated secondary antibody for 45 mins. Then, the cells were washed with PBS and detected by flow cytometry.

A comet assay was used to detect DNA damage using a comet assay kit (Beyotime, CC2041S) according to the manufacturer's protocol. H22 cells were evenly dropped onto agarose electrophoresis slides and then immersed in 4 °C lysis buffer for 1 h. The slides were placed in a horizontal electrophoresis tank and electrophoresed at 25 V in a 4 °C refrigerator in the dark for 30 min. The slides were removed and dried at 37 °C for 10 mins, and stained with ethidium bromide (EB) solution, acridine orange (AO) solution, or DAPI solution, respectively, in the dark for 20 mins. Electrophoresis images were captured via a confocal microscope (Chengdu Lilai Biotechnology Co., Ltd).

To evaluate whether Co^2+^ can modulate the IFN-β response elicited by STING agonists, mouse bone marrow-derived dendritic cells (BMDCs) were obtained from MeisenCTCC and cultured following the supplier’s protocol. BMDCs were seeded at a density of 1 × 10^5^ cells per well in 96-well plates and treated with CoF_2_ at concentrations ranging from 0 to 400 μM, either in the presence or absence of 5 μM cGAMP (Invivogen). Following a 24-hour incubation at 37 °C with 5% CO_2_, culture supernatants were harvested for IFN-β quantification via ELISA.

Annexin V-FITC/PI was used to detect cell apoptosis and pyroptosis. H22 cells (1 × 10^5^/well) were seeded in 6-well plates and treated with 100 μM LOCoF_2_ for 12 hours. Single cell suspensions were further obtained, stained with Annexin-FITC and PI, and analyzed by flow cytometry. Moreover, 2 × 10^4^ H22 cells were evenly seeded in a confocal dish, and the cell morphology was determined via confocal microscopy.

For mitochondrial morphology detection, biological transmission electron microscopy (TEM) was used. Briefly, H22 cells (1 × 10^5^/well) were seeded in a dish and treated with 100 μM LOCoF_2_ for 12 h. Fixation: the sample was pre-fixed with 2.5% glutaraldehyde and then re-fixed with 1% osmium tetroxide. Dehydration: Dehydration with acetone were performed step by step, and the concentration gradient of the dehydrating agent was 30%→50%→70%→80%→90%→95%→100% (100% concentration was changed 3 times). Infiltration: Dehydrating agent and Epon-812 embedding agent were infiltrated in the ratio of 3:1, 1:1, and 1:3 respectively. Embedding: Embedding with pure Epon-812 embedding agent. Ultrathin sectioning: An ultrathin sectioning machine was used to make 60~90 nm ultrathin sections, which were scraped t onto a copper grid. Staining: first stain with uranyl acetate for 10~15 min, then stain with lead citrate for 1~2 min, and stain at room temperature. Finally, images were obtained via TEM.

To verify the expression of proteins related to pyroptosis, stemness, and STING, H22 cells (1 × 10^7^ per well) were seeded in 6-well plates, and the cells in different treatment groups were incubated at 37 °C for 24 h. Subsequently, the cells were washed twice with PBS, and the proteins were collected with cell lysis buffer for standard western blot analysis. Briefly, proteins were transferred to polyvinylidene fluoride (PVDF) membranes and then blocked with 5% bovine serum albumin (BSA) for 1 h. Primary antibodies were incubated with the corresponding proteins overnight at 4 °C, washed with PBST for half an hour, incubated with secondary antibodies for 2 h at room temperature, and washed with PBST for half an hour. The signals were visualized via ECL using a ChemiDoc Touch imaging system (General Electric, AI600UV, USA).

## Animal experiments

Female Balb/c mice (6-8 weeks old), female Nude mice (6-8 weeks old) and male Sprague-Dawley (SD) rats (200-250 g) were purchased from Changzhou cavens Laboratory Animal Co., Ltd. All the animals were used in accordance with protocols approved by the Experimental Animal Center of Soochow University. To establish the subcutaneous H22 tumor model, H22 cells (2 × 10^6^) suspended in 25 μL of PBS and 25 μL of Matrigel Matrix (MeisenCTCC-MS0101ZY) were injected subcutaneously into the right side of each Balb/c mouse. To establish the subcutaneous HuH7 tumor model, HuH7 (RRID: CVCL_0336) cells (3 × 10^6^) suspended in 25 μL of PBS and 25 μL of Matrigel Matrix were injected subcutaneously into the right side of each Nude mice. To establish the orthotopic N1-S1 tumor model, N1-S1 (RRID: CVCL_3551) cells (6 × 10^6^) suspended in 75 μL of PBS containing 30% Matrigel matrix were injected into the left lobe of the liver of each SD rat under anesthesia using a 25-gauge syringe needle. By conducting a detailed comparison and verification on the ICLAC (International Cell Line Authentication Committee), it was confirmed that these cell lines were not among the previously disclosed contaminated cell lines, and they were not contaminated during the entire cultivation and experimental process. The LOCoF_2_ dosage for all animal experiments was 5 mg/kg.

To evaluate the metabolomics of tumors after oxaliplatin treatment, mice bearing subcutaneous H22 tumors (~120 mm^3^) were treated with oxaliplatin (MedChemExpress, HY-17371, 5 mg/kg/every 3 days, intraperitoneally). Tumor volume and mouse weight were recorded every 2 days. After 21 days, the mice were killed, and tumor tissues were obtained for non-targeted metabolomics analysis and lactate content determination.

To evaluate the effect of LOx on oxaliplatin treatment, mice bearing subcutaneous H22 tumors (~120 mm^3^) were randomly divided into 4 groups (n = 6) and received the following intratumoral injections: Group I, PBS. Group II: LOx (50 μL, 5 mg kg^-1^, 3 times a week, i.t. injection). Group III: Oxaliplatin (5 mg kg^−1^, twice a week, i.p. injection). and Group IV: a combination of both agents at the aforementioned doses. Tumor volumes and mouse body weights were recorded every 2 days.

To assess the immunological profiles of oxaliplatin-sensitive and oxaliplatin-resistant murine models, mice bearing subcutaneous H22 tumors (approximately 120 mm^3^) were randomly allocated into two cohorts (n = 10) and administered oxaliplatin (5 mg kg^−1^, intraperitoneally, twice weekly). On day 8 post-treatment, the mice were euthanized, and tumor tissues were excised and mechanically dissociated. Each tumor specimen was bisected; one portion was utilized for protein extraction and subsequent Western blot analysis of γ-H2AX, while the remaining portion was processed into a single-cell suspension. For detailed immunophenotypic characterization, cells were stained with monoclonal antibodies targeting CD8^+^ T lymphocytes and Tregs, followed by flow cytometric analysis to quantify relevant immunological parameters.

To assess the immunological profile following LOx in combination with oxaliplatin therapy, mice bearing subcutaneous H22 tumors (approximately 120 mm^3^) were randomly assigned to four groups (n = 6 per group) and subjected to the following interventions: Group I received PBS, Group II was administered LOx (5 mg/kg, intratumorally), Group III received oxaliplatin (5 mg/kg, intraperitoneally, twice weekly), and Group IV was treated with both LOx and oxaliplatin. Eight days post-treatment, the mice were sacrificed, and tumor tissues were excised and mechanically dissociated. Each tumor was bisected; one half was processed for protein extraction and subsequent Western blot analysis of γ-H2AX, while the other half was used to generate a single-cell suspension. For detailed immunophenotyping, cells were stained with antibodies targeting CD8^+^ T lymphocytes and Tregs, followed by flow cytometric analysis to quantify immune cell populations.

To evaluate the therapeutic effect of LOCoF_2_, mice bearing subcutaneous H22 tumors (~120 mm^3^) were randomly divided into 4 groups (n = 6) and received the following intratumoral injections: Group I, PBS. Group II: LOx. Group III: CoF_2_, and Group IV: LOCoF_2_. The intratumoral injection dose of LOx and CoF_2_ was 5 mg kg^-1^. Thereafter, the length and width of each tumor and the body weight of each mouse were recorded every other day using a vernier caliper and an electronic scale, respectively. The tumor volume was calculated according to the formula tumor volume = (width*width*length)/2. A mouse was considered dead when the tumor volume reached 1500 mm^3^. In addition, two days after intratumoral injection, one mouse in each group was randomly selected and sacrificed, and the tumors were collected for H&E and immunohistochemical staining.

To assess the biosafety profile of LOCoF_2_, subcutaneous injections of LOCoF_2_ were administered to 15 Balb/c mice. At predetermined intervals (days 0, 7, 15, and 30), three mice were randomly selected and euthanized. Serum samples were collected for biochemical analysis of ALT, AST, TBIL, and CREA levels. Major organs, including the heart, liver, spleen, lungs, and kidneys, were harvested for histopathological examination via hematoxylin and eosin (H&E) staining.

To evaluate the immune status after LOCoF_2_ treatment, mice bearing subcutaneous H22 tumors (~120 mm^3^) were randomized into 4 groups (n = 5) and received the following intratumoral injections: Group I: PBS. Group II: LOx. Group III: CoF_2_, Group IV: LOCoF_2_. H22 mice receiving different treatments were euthanized on day 7, and tumor tissues and proximal inguinal lymph nodes were carefully excised, suspended and cut into single cell suspensions. To further analyze the immune effects, cells were stained with the corresponding immune cell flow cytometry antibodies. Immunological parameters were evaluated by flow cytometry. Additionally, supernatants from tumor tissues and lymph nodes were collected to measure the levels of cytokines, including IL-1β, TNF-α, IFN-γ and IFN-β via ELISA kits.

For transcriptome analysis, the mice bearing subcutaneous H22 tumors (~150 mm^3^) were randomly divided into two groups, the control group (n = 3) and the LOCoF_2_ (5 mg/kg, i.t. injection, n = 4) group. Tumor tissues were extracted for mRNA sequencing analysis after 7 days of treatment. Total cell mRNA was extracted with TRIzol reagent, and after verification of RNA integrity and purity, high-throughput sequencing of mRNA was performed at Suzhou Jinweizhi Technology Co., Ltd. Statistical analysis of the significant differential expression of genes was performed by log2|FC| ≥ 1.5 and q value (FDR, p adj) ≤ 0.05. p value ≤ 0.05 was considered significant for GO enrichment, and q value ≤ 0.05 was considered significant for KEGG enrichment.

To evaluate whether LOCoF_2_ enhances the therapeutic effect of α-PD-1, mice bearing subcutaneous H22 tumors (~120 mm^3^) were randomly divided into four groups (n = 5) and received the following treatments: Group I, PBS, Group II: α-PD-1 1 (0.1 mg/mL, 200 µL, i.v. injection), Group III: LOCoF_2_, and Group IV: LOCoF_2_ (5 mg/kg, i.t. injection) + α-PD-1 (0.1 mg/mL, 200 µL, i.v. injection). Tumor size was measured every 2 days, and tumor volume was calculated according to the formula tumor volume = (width × width × length)/2. The mice were considered dead when the tumor volume reached 1500 mm^3^. In addition, two days after intratumoral injection, one mouse in each group was randomly selected and sacrificed, and the tumors were collected for H&E and immunohistochemical staining (AiFang biological).

To evaluate the TACE effect of Lipiodol + LOCoF_2_ on orthotopic N1-S1 hepatocellular carcinoma tumors (~250 mm^3^), 20 rats bearing orthotopic N1-S1 hepatocellular carcinoma tumors (~250 mm^3^) were randomly divided into four groups (n = 5) and treated as follows: Group I, control group; Group II, transarterial embolization (Lipiodol); Group III, transarterial embolization (Lipiodol + LOCoF_2_); and Group IV, transarterial embolization (Lipiodol + LOCoF_2_ + Oxa). On day 0, the rats in Group II, Group III, and Group IV were treated with transarterial embolization with Lipiodol, Lipiodol + LOCoF_2_, and Lipiodol + LOCoF_2_ + Oxa, respectively. The injection doses of LOx, LOCoF_2_, and Oxa were 200 uL, and 5 mg kg^-1^, respectively. The tumor volumes of the rats in each group were recorded via a 7.0-Tesla small animal MR scanner (Bruker Pharmascan, Ettlingen, Germany) on days 0, 4, 7, and 14 after treatment. The tumor volume (V) was calculated on the basis of the tumor echogenicity in the superior-inferior (SI), lateral-medial (LM), and anterior-posterior (AP) planes as follows: V (mm^3^) = 3π/4 × 1SI/2 × 1LM/2 × 1AP/2 to further evaluate the therapeutic effect. One rat in each group was killed on the 8^th^ day, and the tumor tissues were collected for H&E and immunohistochemical staining.

# Supporting Figures


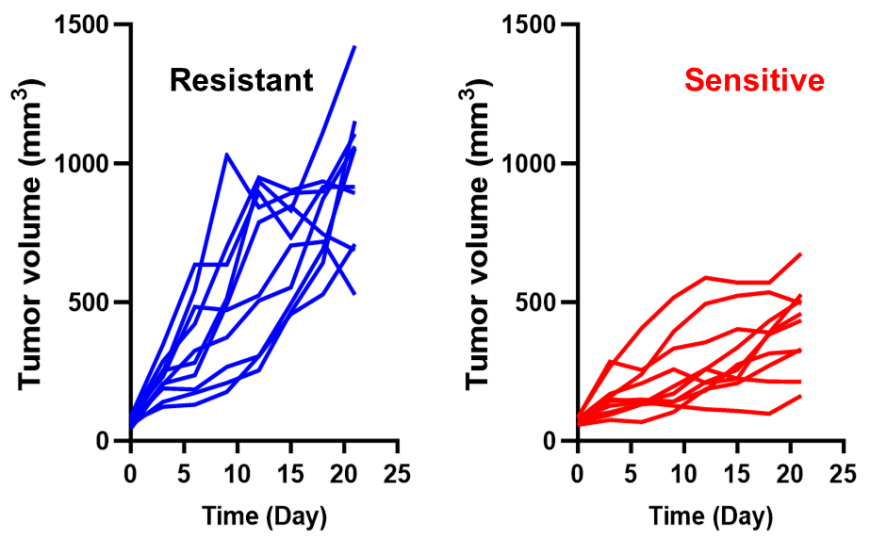


**Figure S1.** Tumor growth curves of Oxa-resistant and Oxa-sensitive individuals.


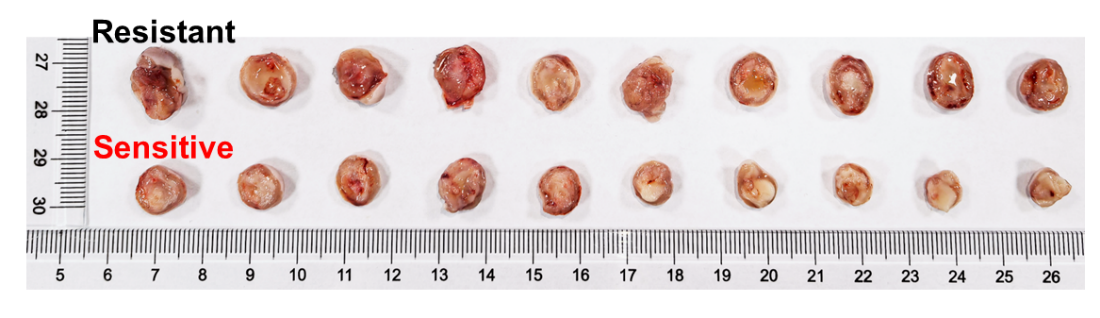


**Figure S2.** Oxa-resistant and Oxa-sensitive tumor images.


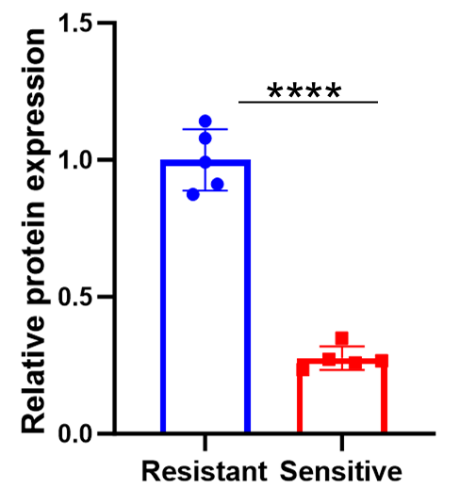


**Figure S3.** Quantitative analysis of relative protein expression levels depicted in **Figure 1F**.

**
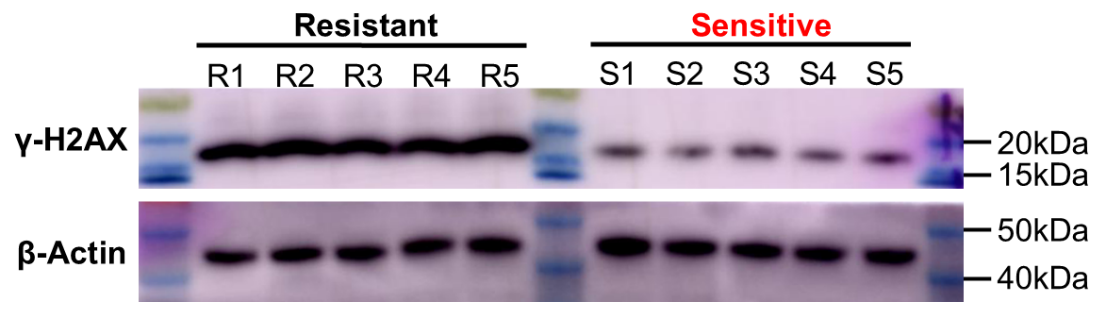
**

**Figure S4.** Complete initial data of western blotting analysis for **Figure 1F**.

**
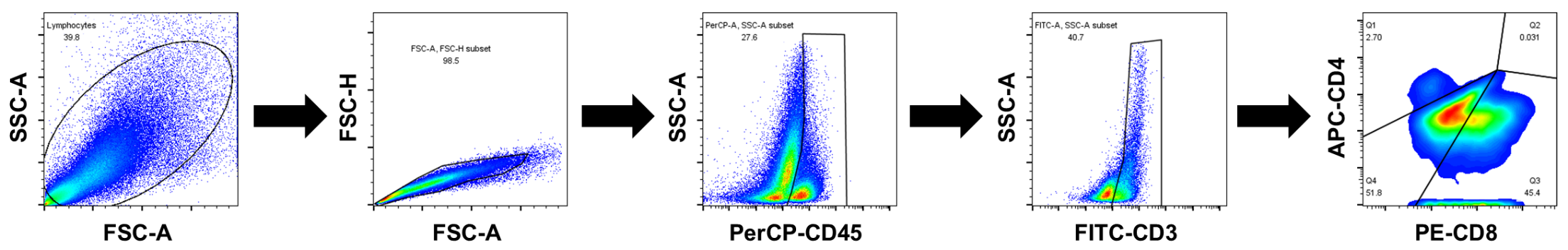
**

**Figure S5.** The gating strategy used to determine the percentages of T cells (CD45^+^CD3^+^CD8^+^) in **Figure 1G&R.**

**
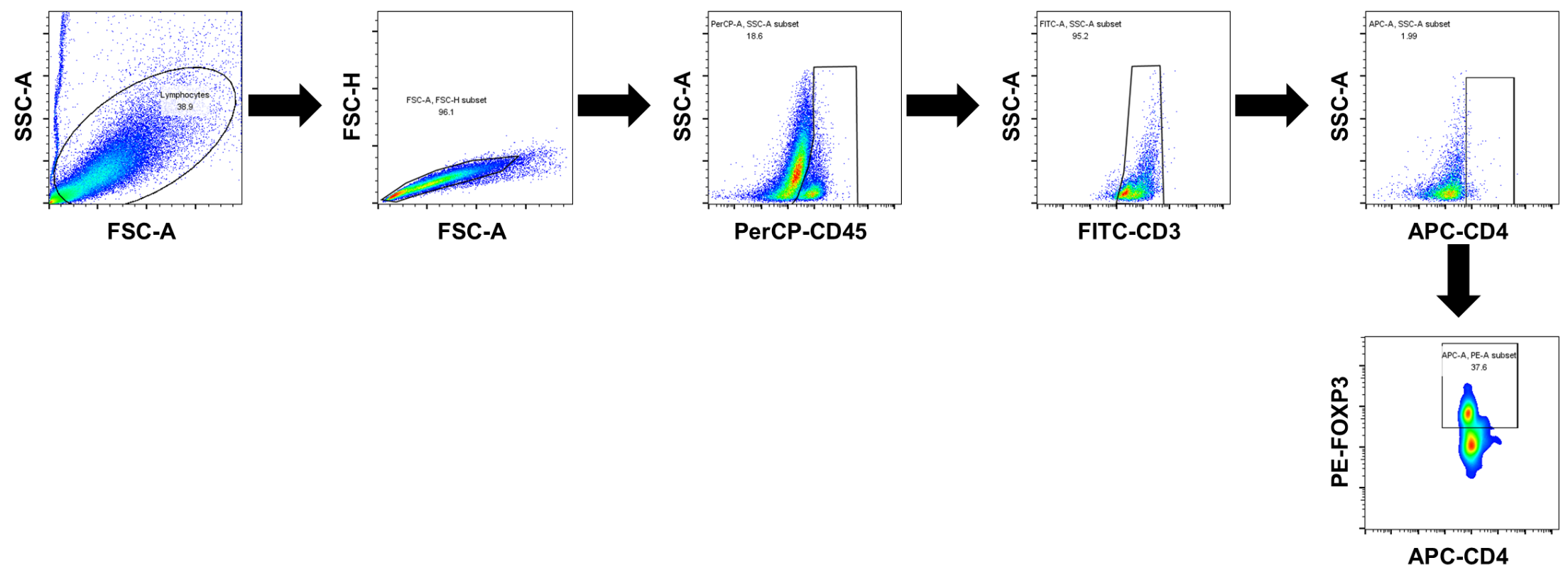
**

**Figure S6.** The gating strategy used to determine the percentages of Tregs (CD45^+^CD3^+^CD4^+^Foxp3^+^) in **Figure 1H, Figure S11**.

**
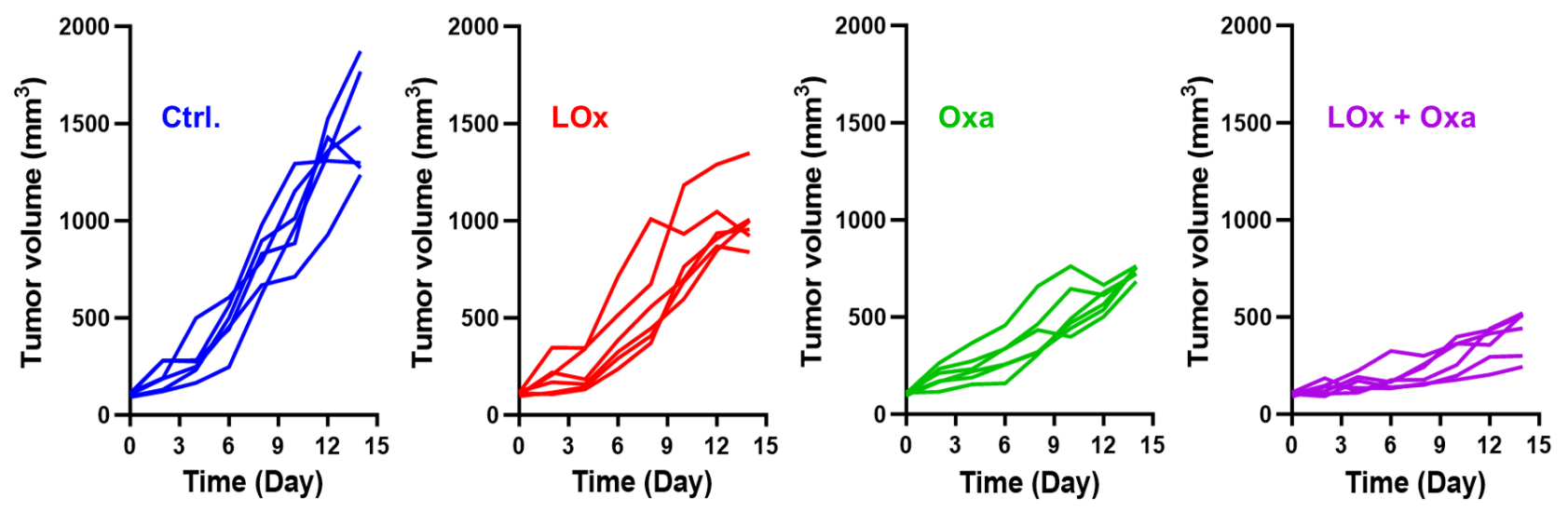
**

**Figure S7.** Individual growth curves of H22 tumors after various treatments.

**
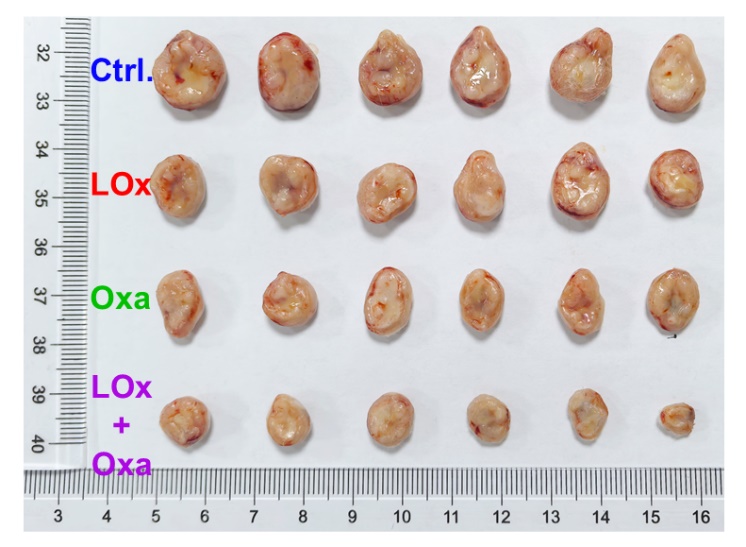
**

**Figure S8.** Tumor images after different treatments.

**
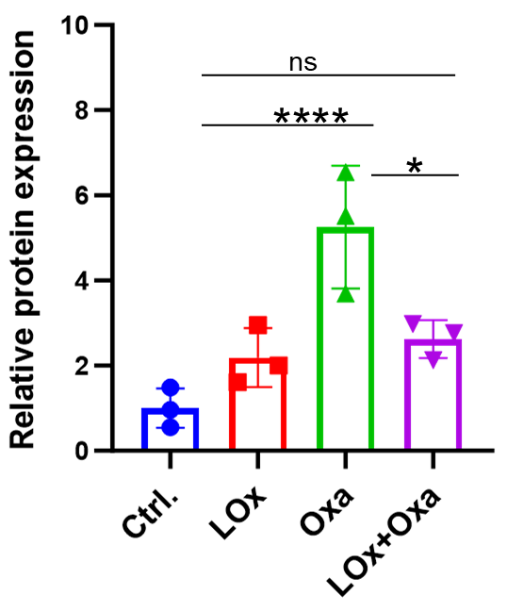
**

**Figure S9.** Quantitative analysis of relative protein expression levels depicted in **Figure 1O**.

**
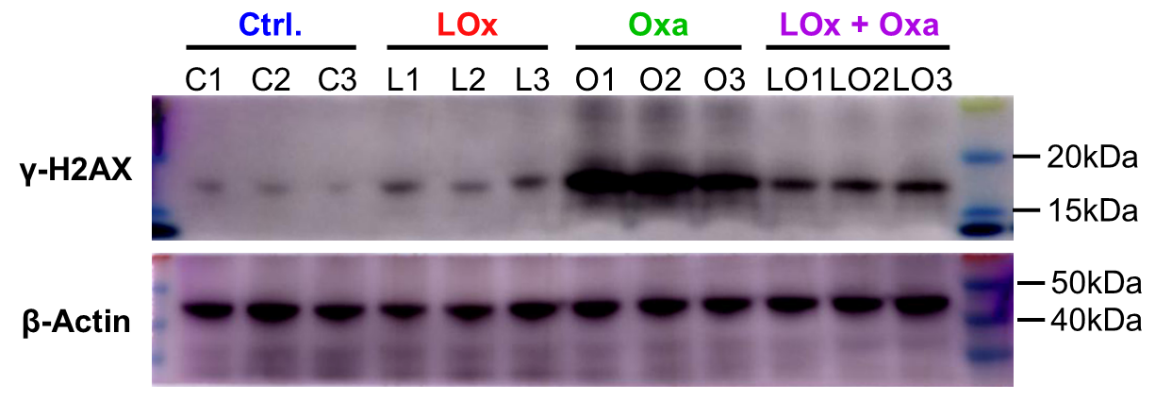
**

**Figure S10.** Complete initial data of western blotting analysis for **Figure 1O**.

**
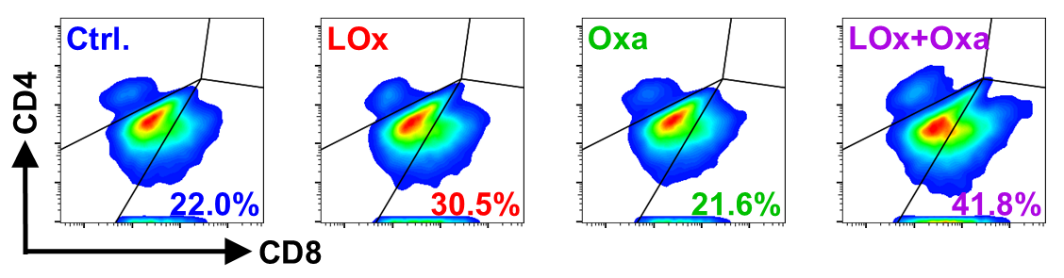
**

**Figure S11.** Flow cytometric analysis was performed on CD45^+^CD3^+^CD8^+^ T cells in tumor tissues after different treatments.

**
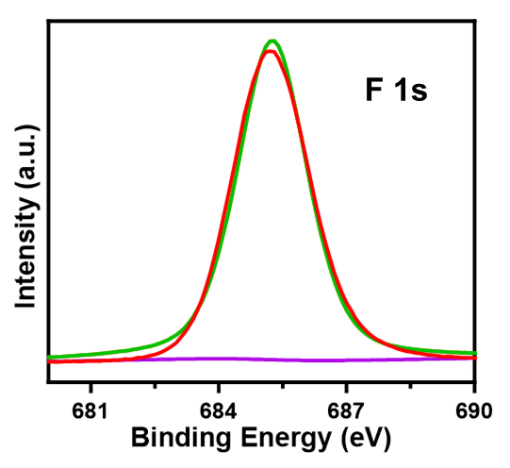
**

**Figure S12.** XPS spectra of F 1s for CoF_2_.

**
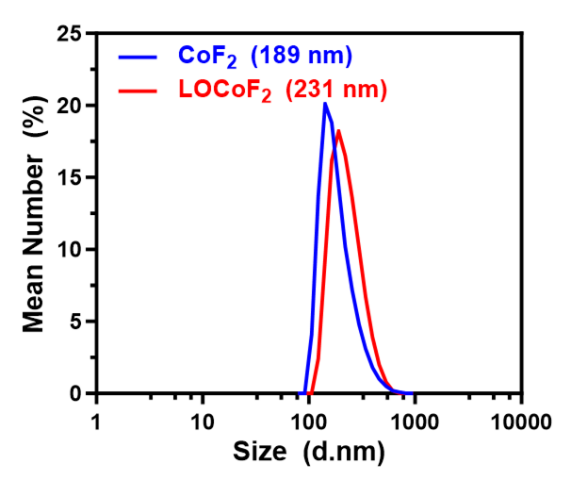
**

**Figure S13.** DLS of CoF_2_ and LOCoF_2_.

**
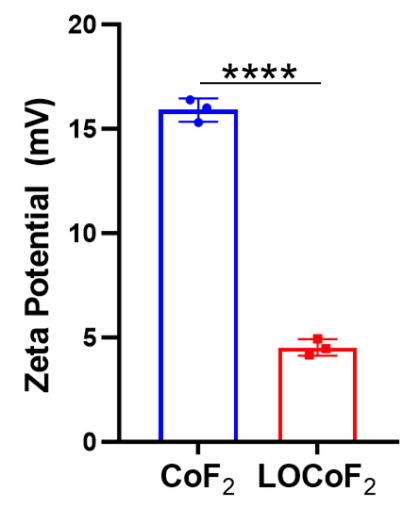
**

**Figure S14.** Zeta potentials of CoF_2_ and LOCoF_2_.

**
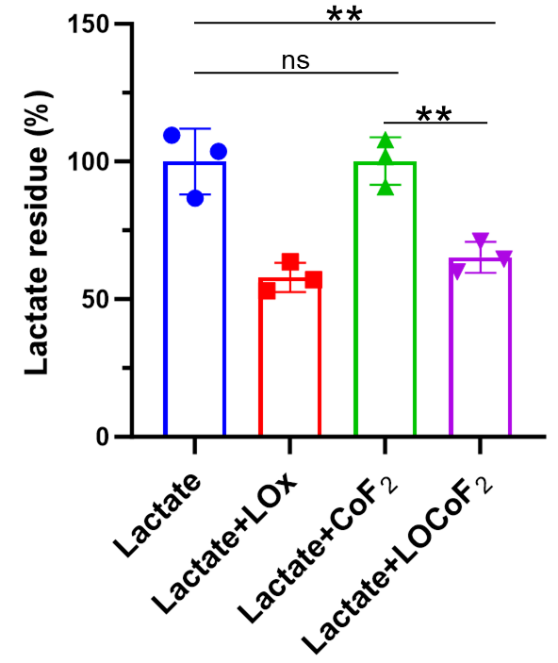
**

**Figure S15.** A lactate kit was used to measure the ability of LOx and LOCoF_2_ to consume lactate.

**
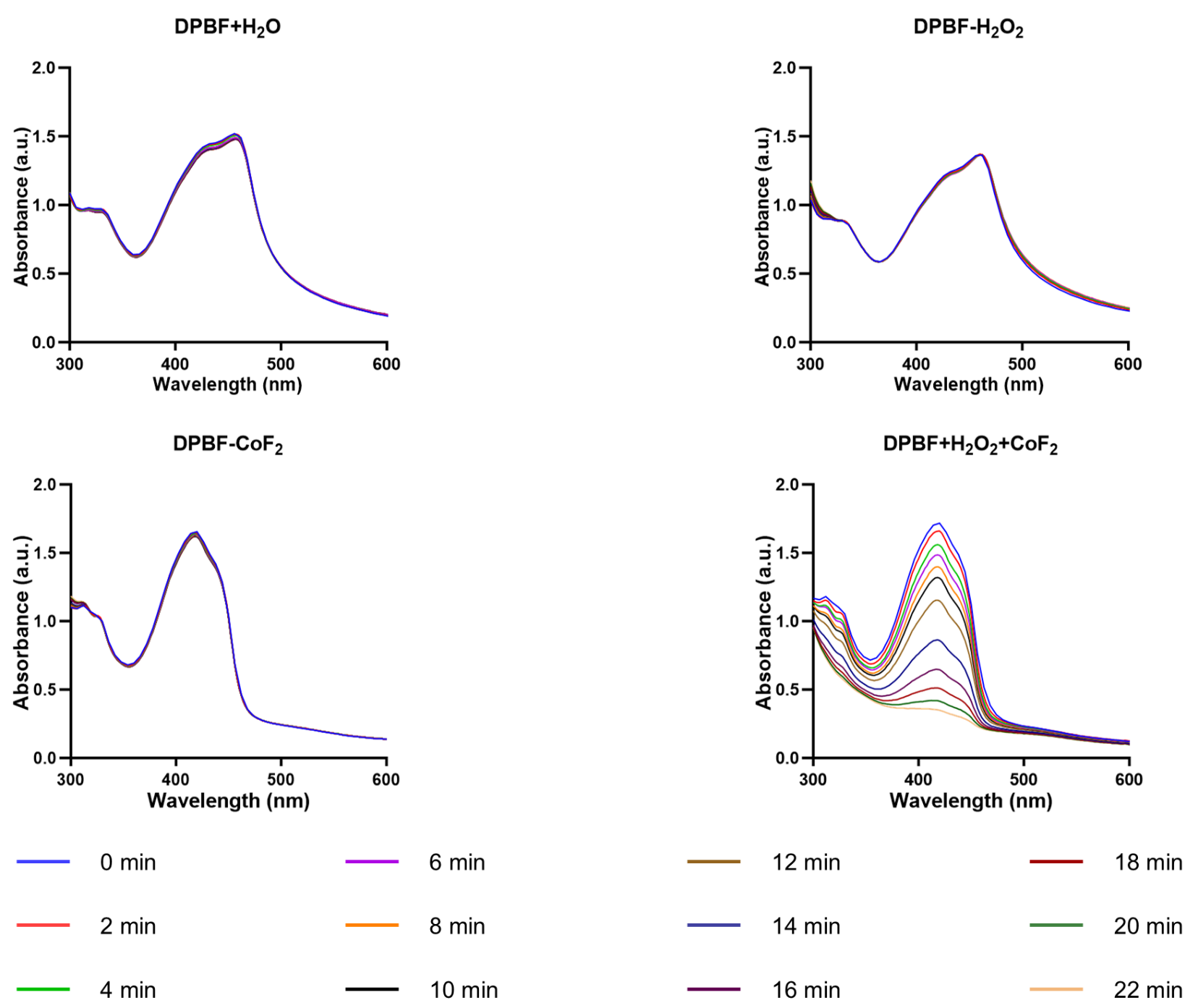
**

**Figure S16.** Individual time-dependent oxidation curves of DPBF by CoF_2_ NPs under H_2_O_2_.

**
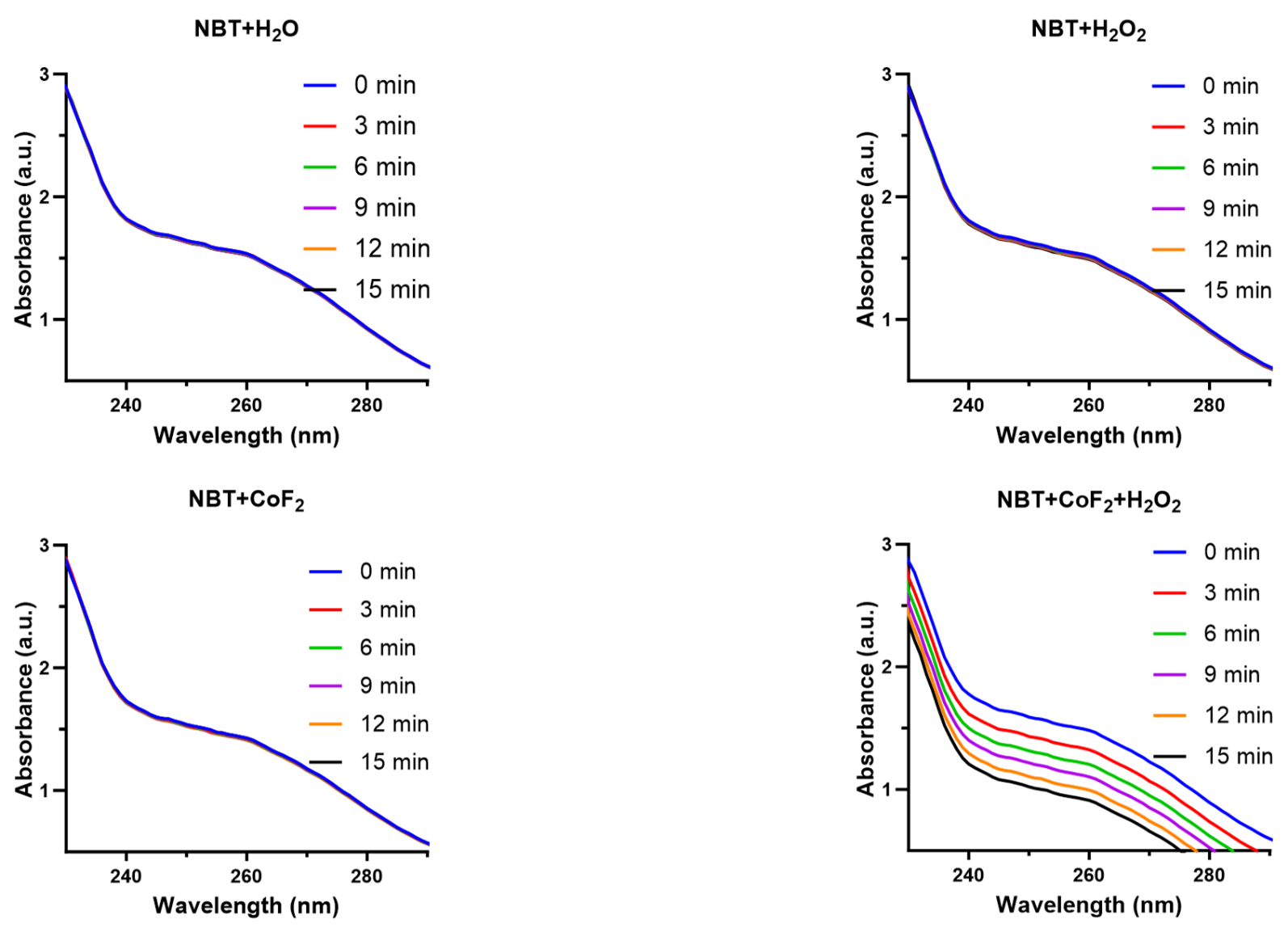
**

**Figure S17.** Individual time-dependent oxidation curves of NBT by CoF_2_ NPs under H_2_O_2_.

**
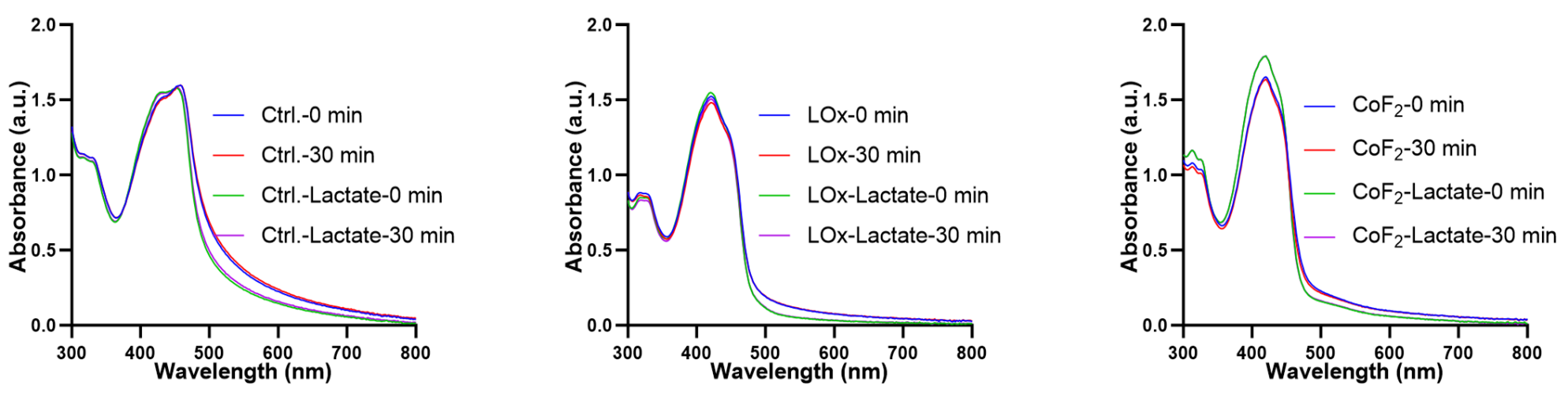
**

**Figure S18.** Individual time-dependent oxidation curves of DPBF by CoF_2_ NPs under lactate.

**
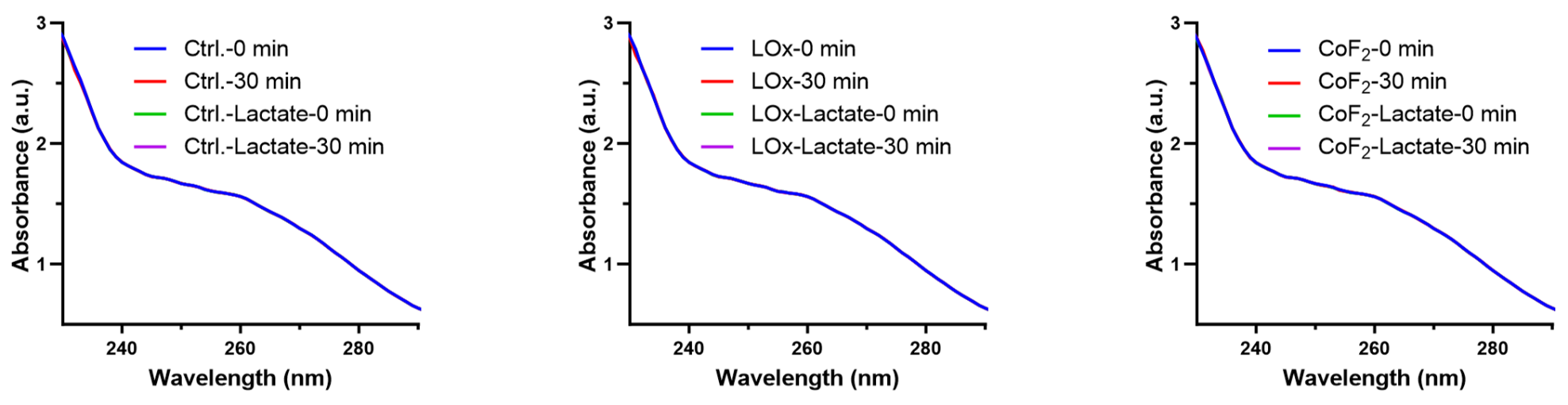
**

**Figure S19.** Individual time-dependent oxidation curves of NBT by CoF_2_ NPs under lactate.

**
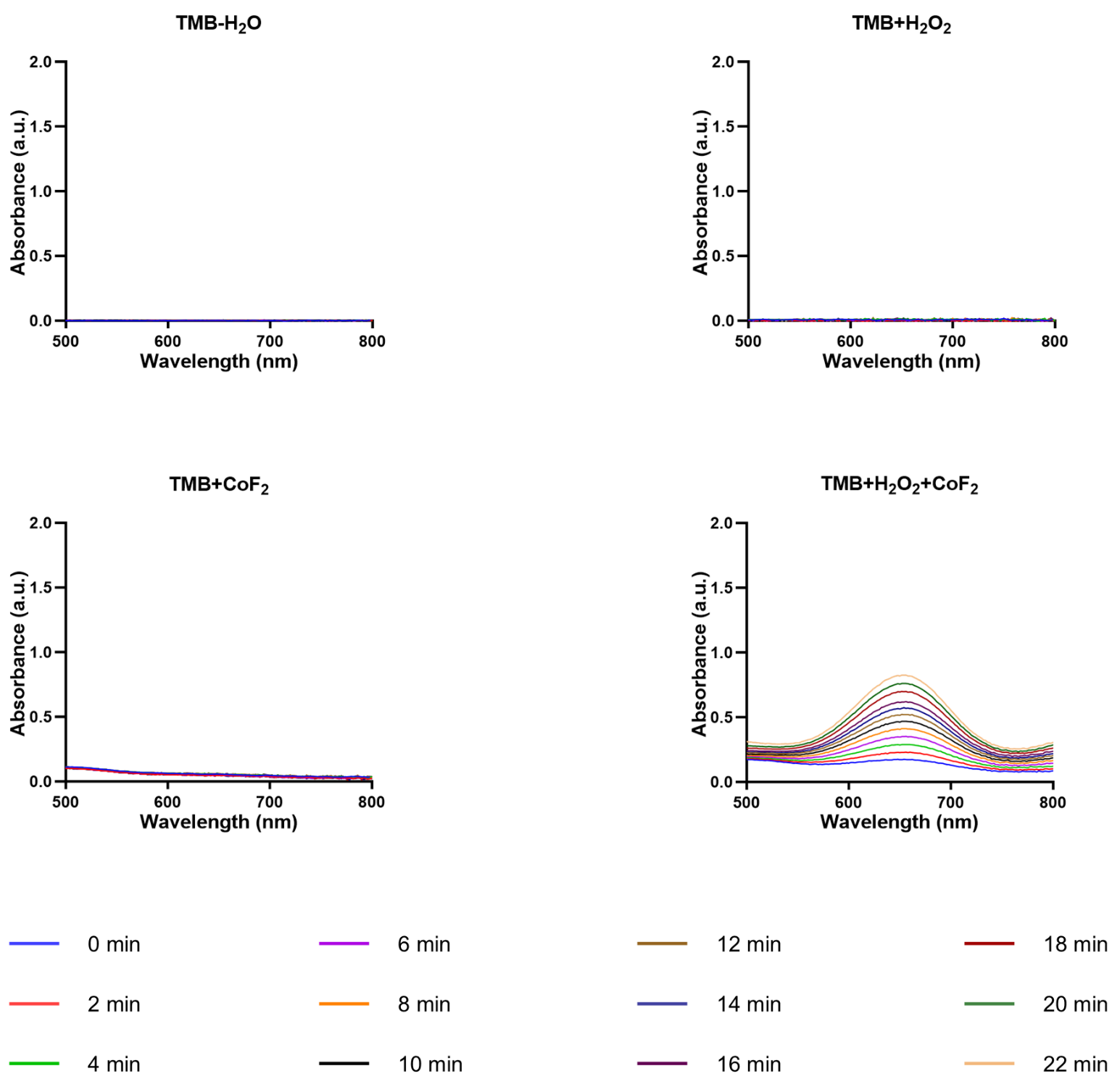
**

**Figure S20.** CoF_2_ individual time-dependent oxidation curves of the TMB probe by CoF_2_ NPs under H_2_O_2_.

**
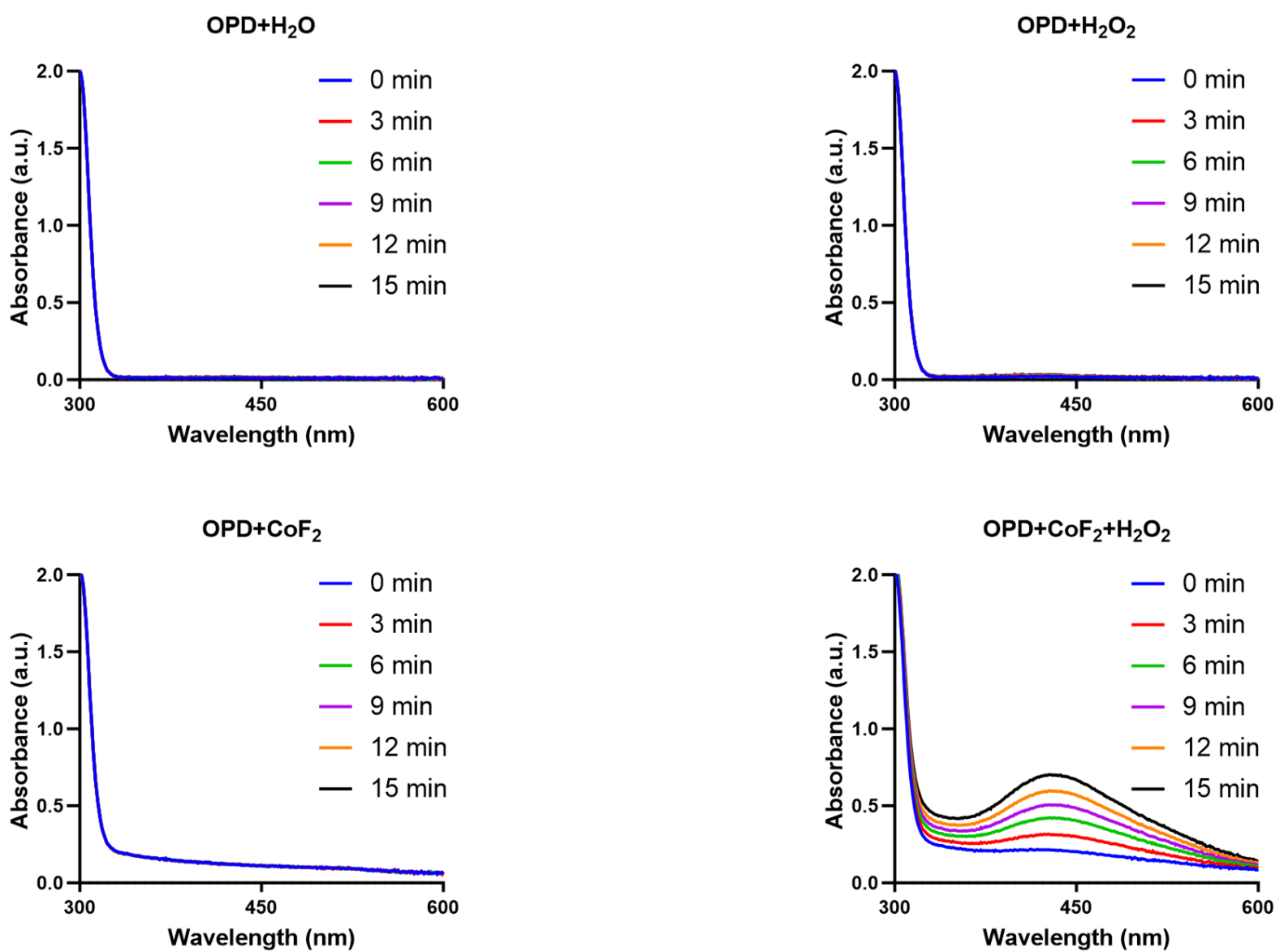
**

**Figure S21.** CoF_2_ individual time-dependent oxidation curves of the OPD probe by CoF_2_ NPs under H_2_O_2_.

**
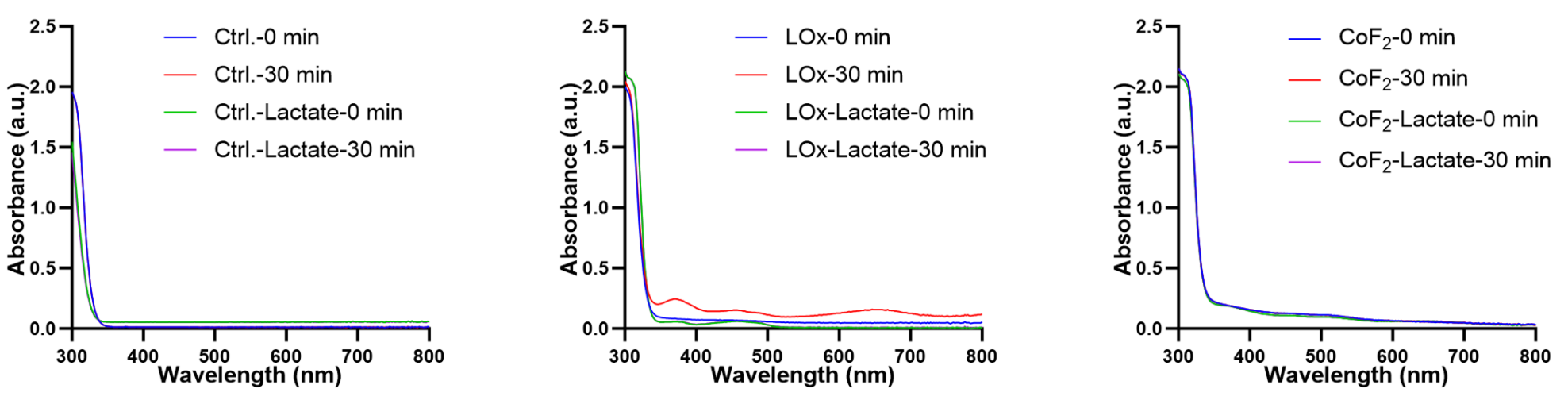
**

**Figure S22.** Individual time-dependent curves of the •OH generation efficiency of LOCoF_2_ NPs under lactate using the TMB probe.

**
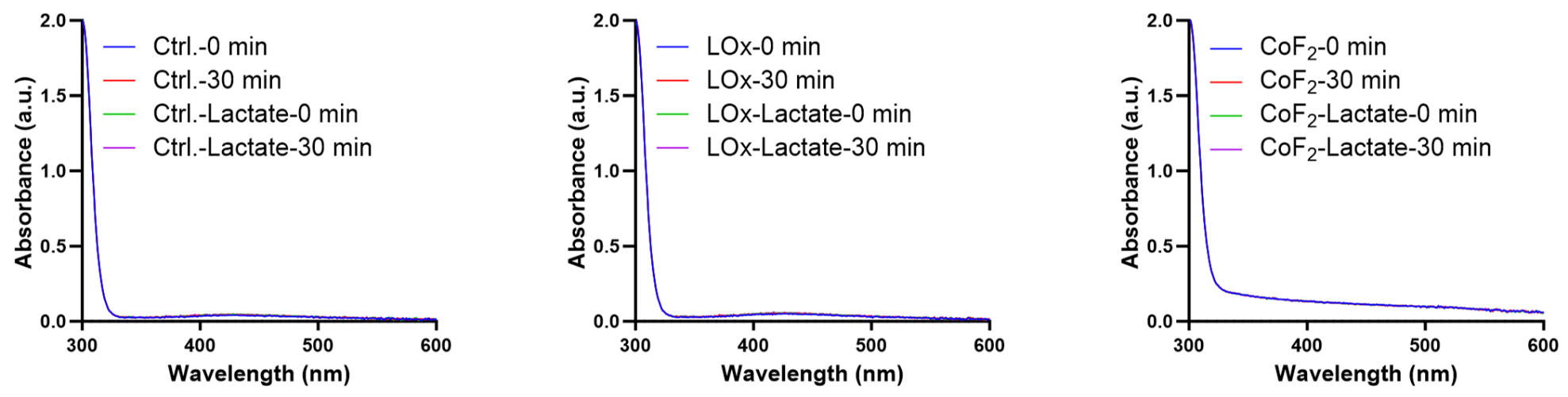
**

**Figure S23.** Individual time-dependent curves of the •OH generation efficiency of LOCoF_2_ NPs under lactate using the OPD probe.

**
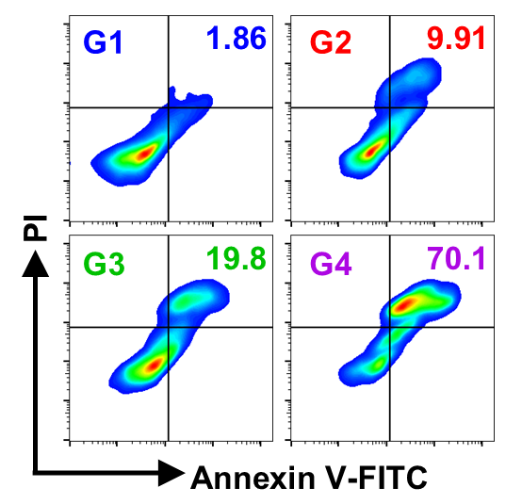
**

**Figure S24.** Flow cytometry analysis of H22 cell apoptosis after various treatments. G1: Ctrl., G2: LOx, G3: CoF_2_, G4: LOCoF_2_.

**
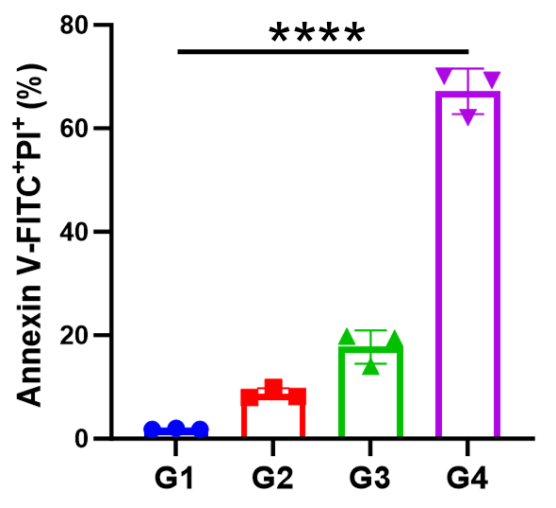
**

**Figure S25.** Flow cytometry quantification of H22 cell apoptosis after various treatments. G1: Ctrl., G2: LOx, G3: CoF_2_, G4: LOCoF_2_.

**
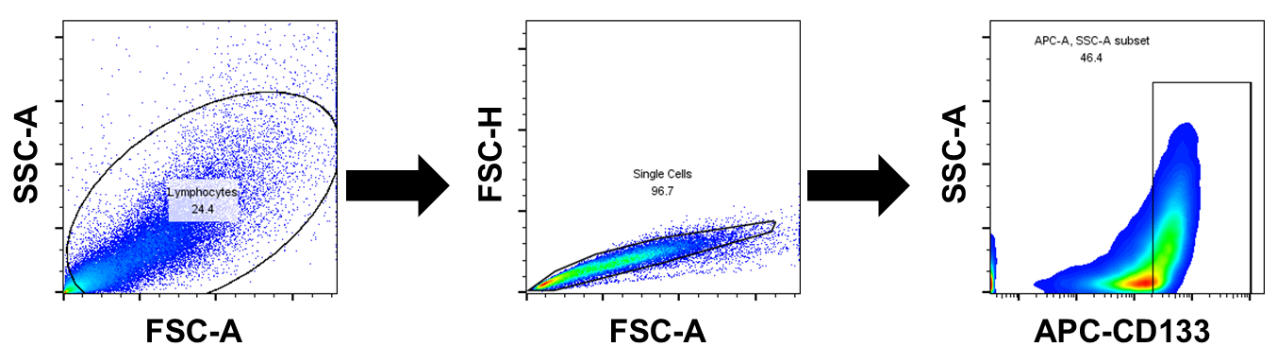
**

**Figure S26.** The gating strategy used to determine the percentages of CSCs (CD133^+^) in **Figure 3H.**

**
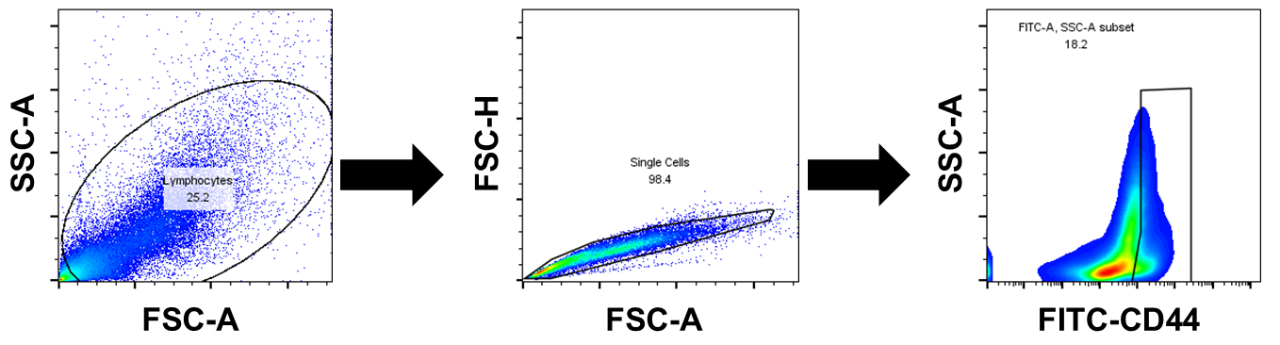
**

**Figure S27.** The gating strategy used to determine the percentages of CSCs (CD44^+^) in **Figure 3J.**

**
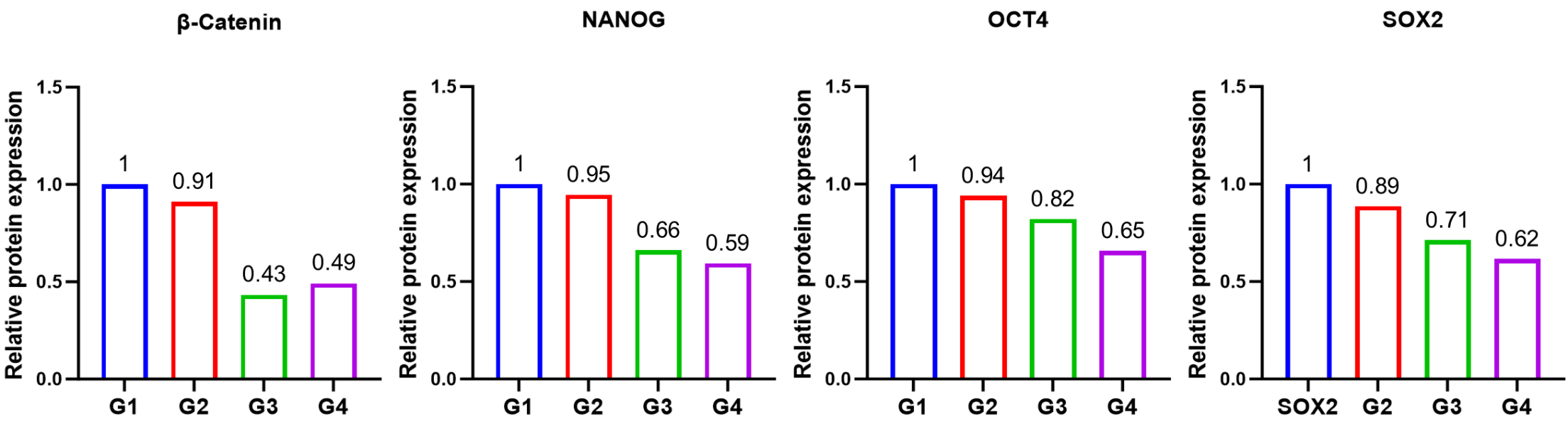
**

**Figure S28.** Quantitative analysis of relative protein expression levels depicted in **Figure 3L**. G1: Ctrl., G2: LOx, G3: CoF_2_, G4: LOCoF_2_.

**
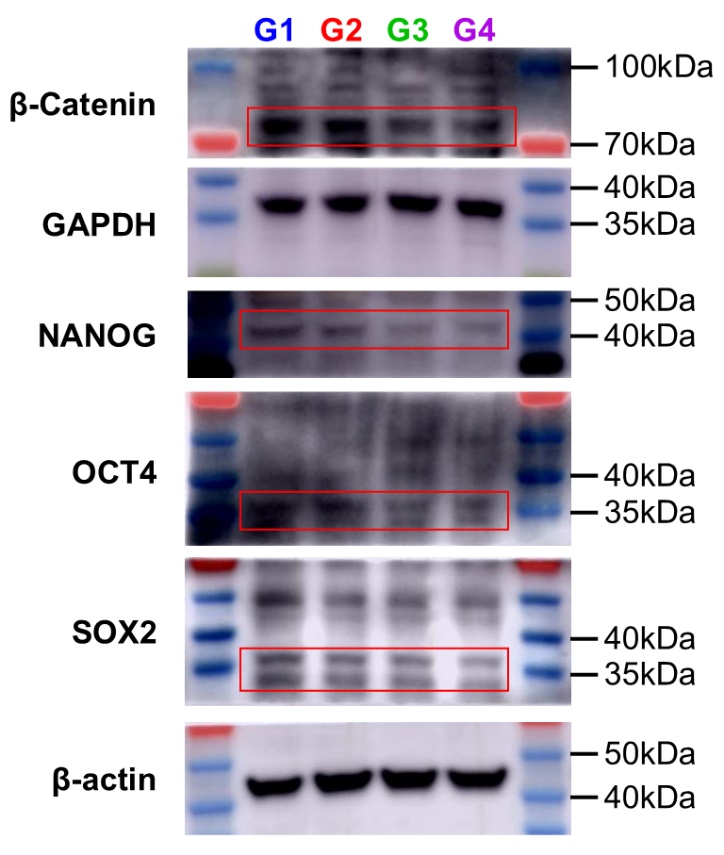
**

**Figure S29.** Complete initial data of western blotting analysis for **Figure 3L**. G1: Ctrl., G2: LOx, G3: CoF_2_, G4: LOCoF_2_.

**
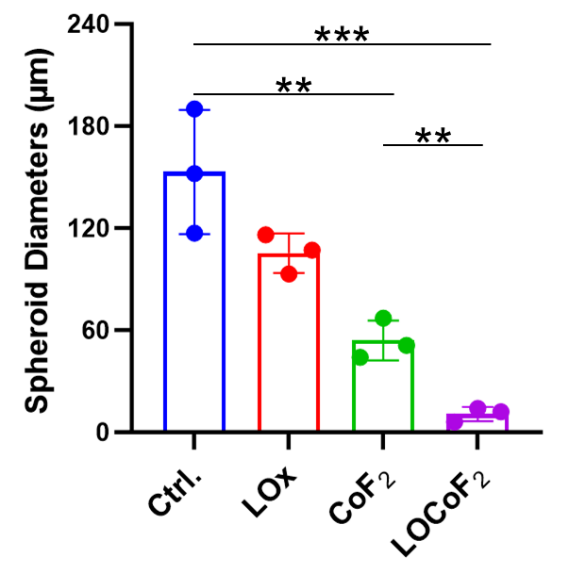
**

**Figure S30.** Statistics of the maximum diameter of H22 cell spheres after various treatments in **Figure 3M**.

**
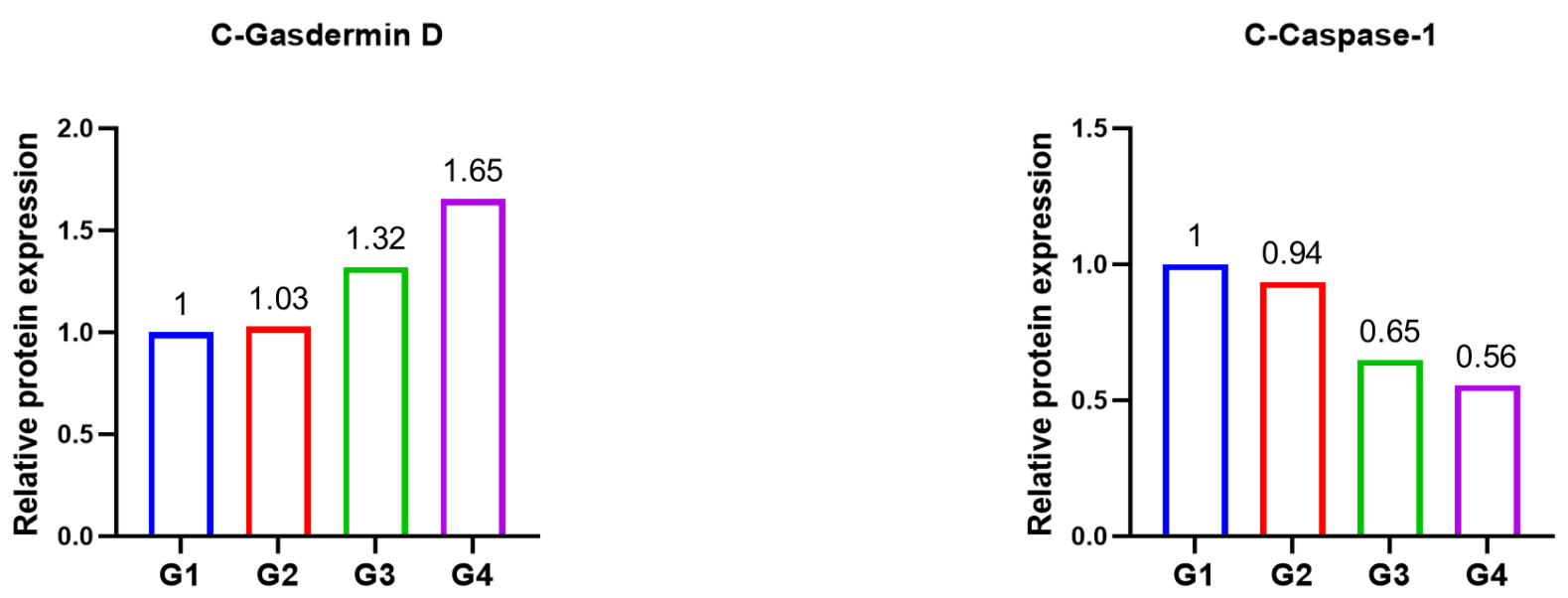
**

**Figure S31.** Quantitative analysis of relative protein expression levels depicted in **Figure 4C**. G1: Ctrl., G2: LOx, G3: CoF_2_, G4: LOCoF_2_.

**
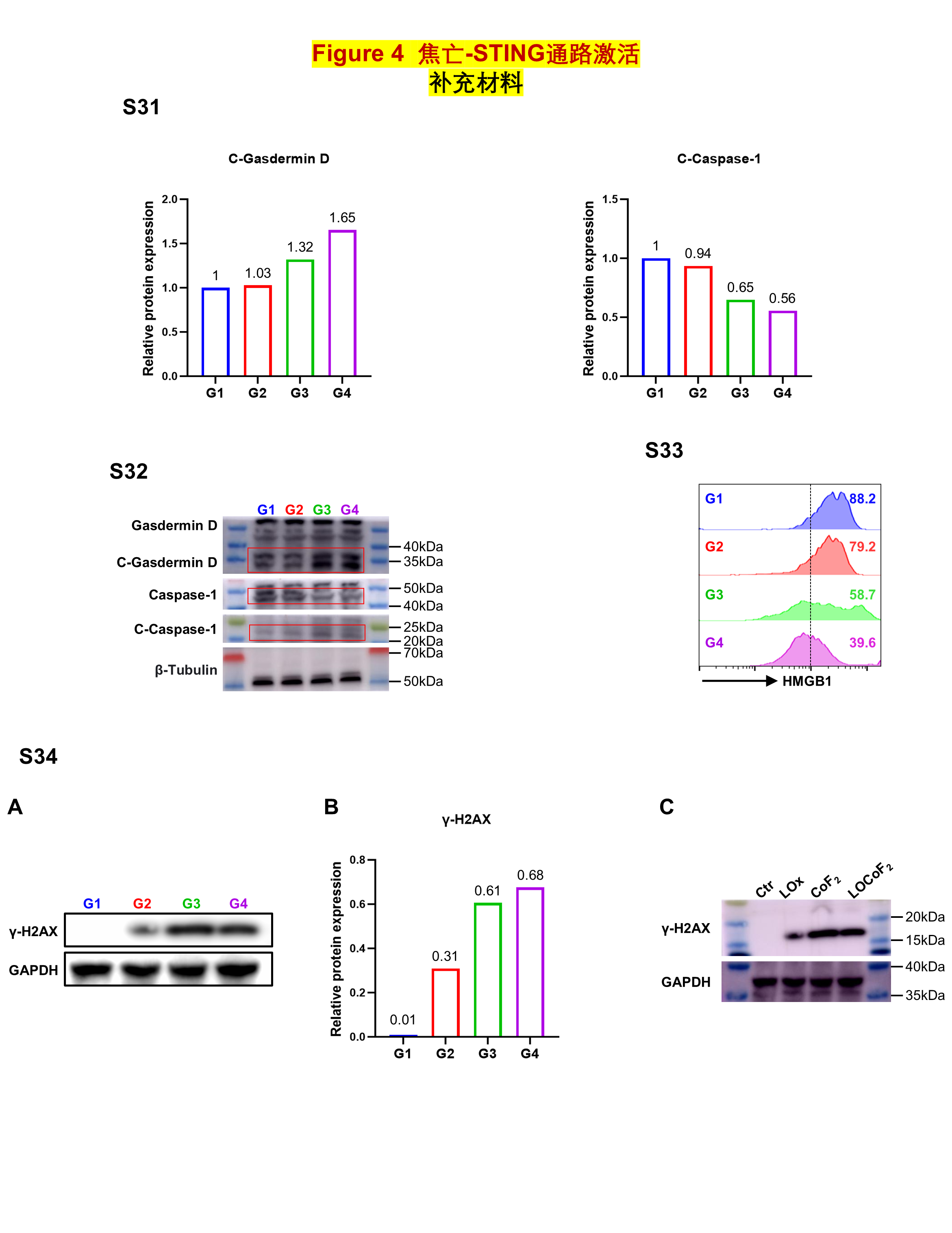
**

**Figure S32.** Complete initial data of western blotting analysis for **Figure 4C**. G1: Ctrl., G2: LOx, G3: CoF_2_, G4: LOCoF_2_.


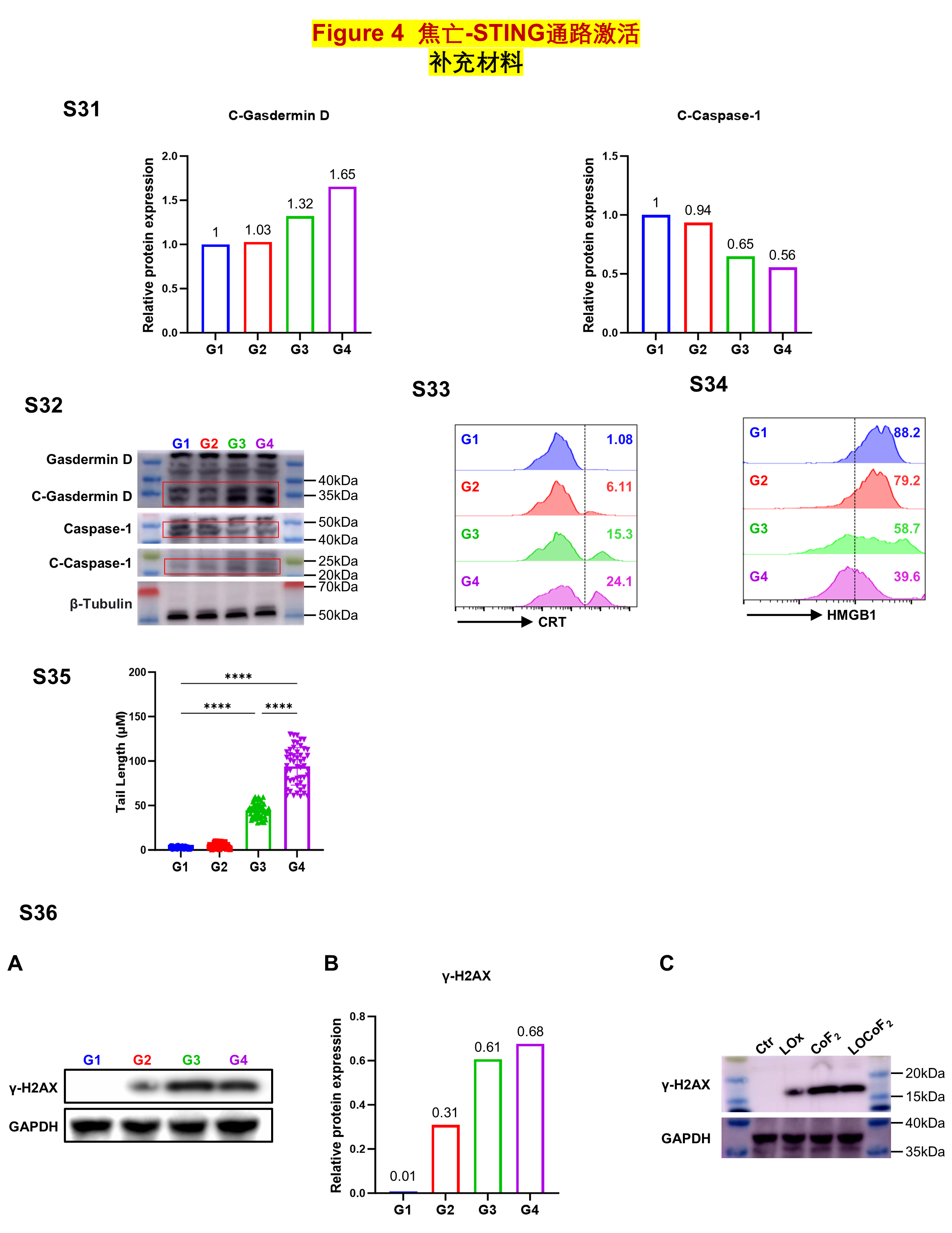


**Figure S33.** Flow cytometry analysis of CRT expression in H22 cells. G1: Ctrl., G2: LOx, G3: CoF_2_, G4: LOCoF_2_.

**
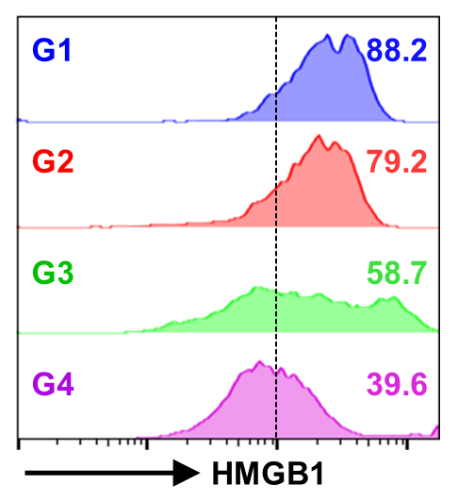
**

**Figure S34.** Flow cytometry analysis of HMGB1 release in H22 cells. G1: Ctrl., G2: LOx, G3: CoF_2_, G4: LOCoF_2_.

**
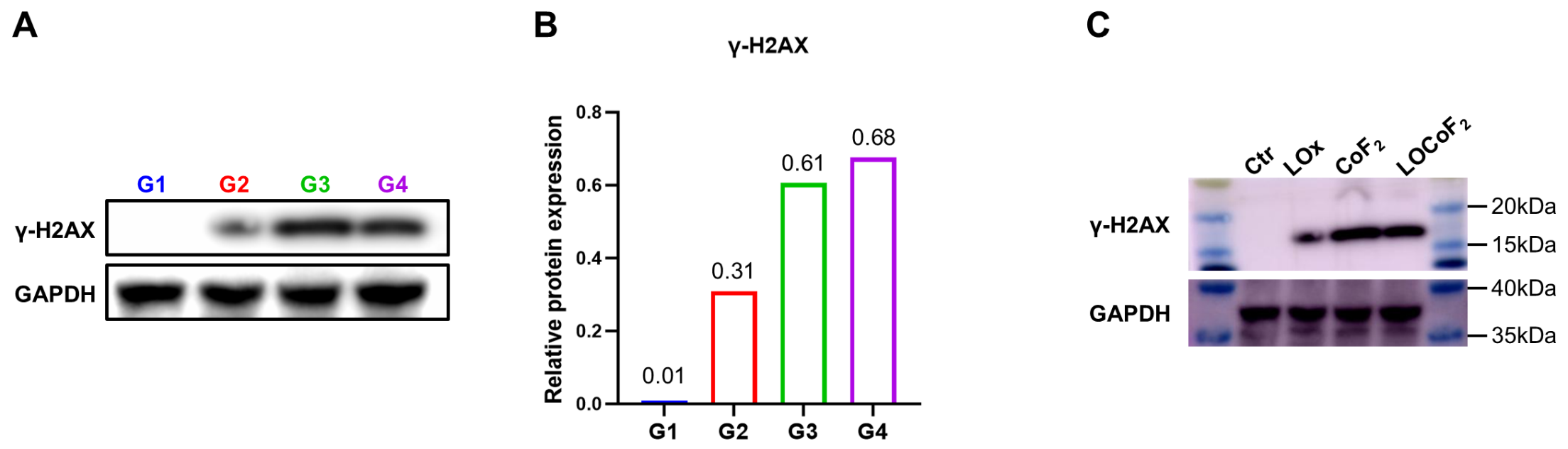
**

**Figure S35. A)** The expression levels of γ-H2AX in tumor tissues after different treatments were detected by Western blot analysis and **B)** quantitative analysis of relative protein expression levels. **C)** Complete initial data of western blotting analysis for **A).** G1: Ctrl., G2: LOx, G3: CoF_2_, G4: LOCoF_2_.

**
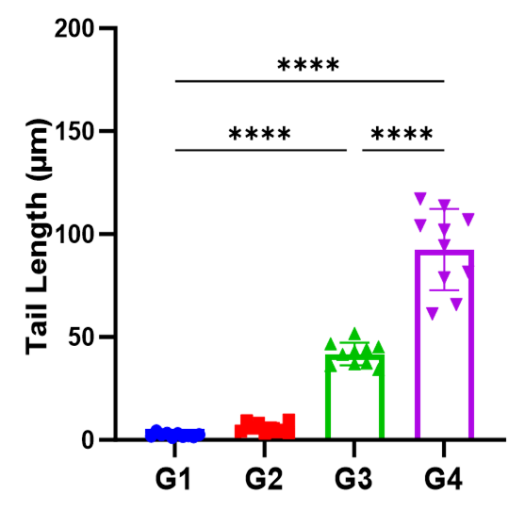
**

**Figure S36.** Quantitative assessment of tail length in H22 cells via comet assay in **Figure 4J.**

**
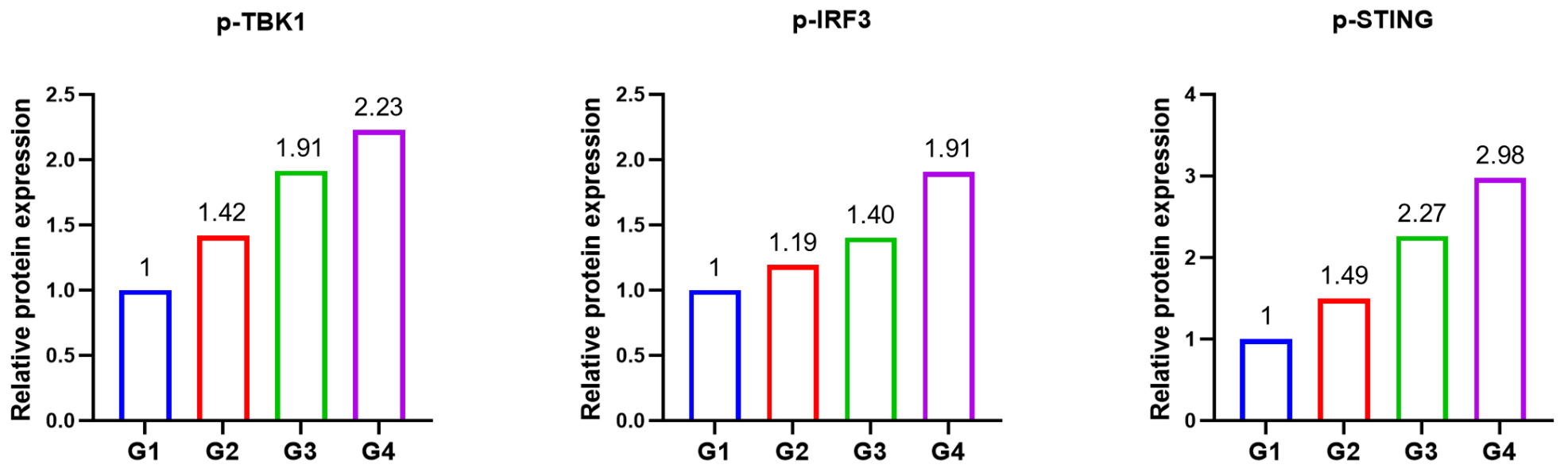
**

**Figure S37.** quantitative analysis of relative protein expression levels in **Figure 4K**. G1: Ctrl., G2: LOx, G3: CoF_2_, G4: LOCoF_2_.

**
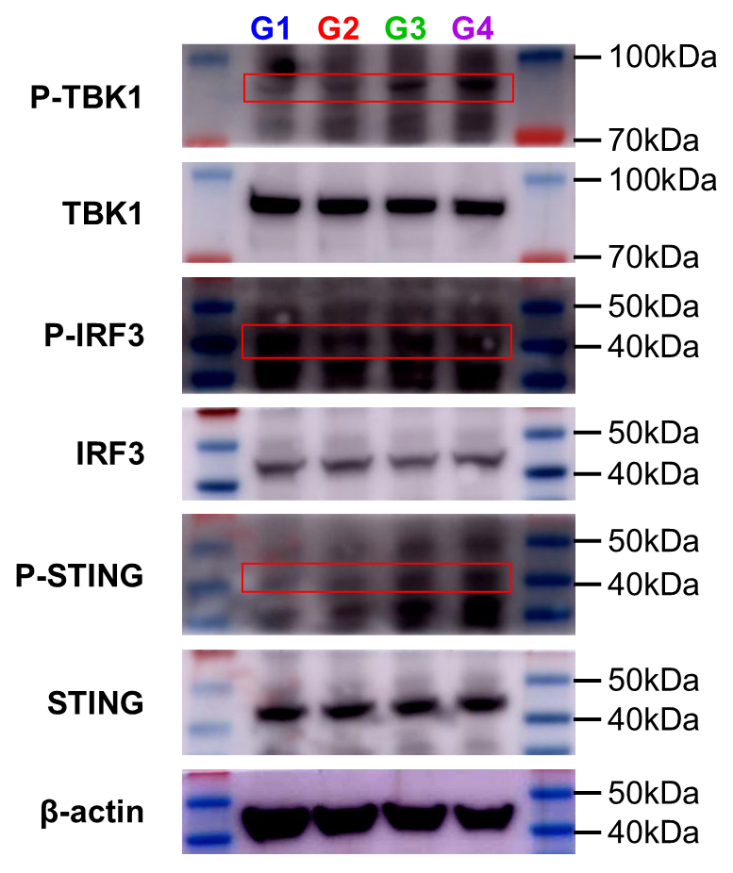
**

**Figure S38.** Complete initial data of Western blotting analysis for **Figure 4K**. G1: Ctrl., G2: LOx, G3: CoF_2_, G4: LOCoF_2_.

**
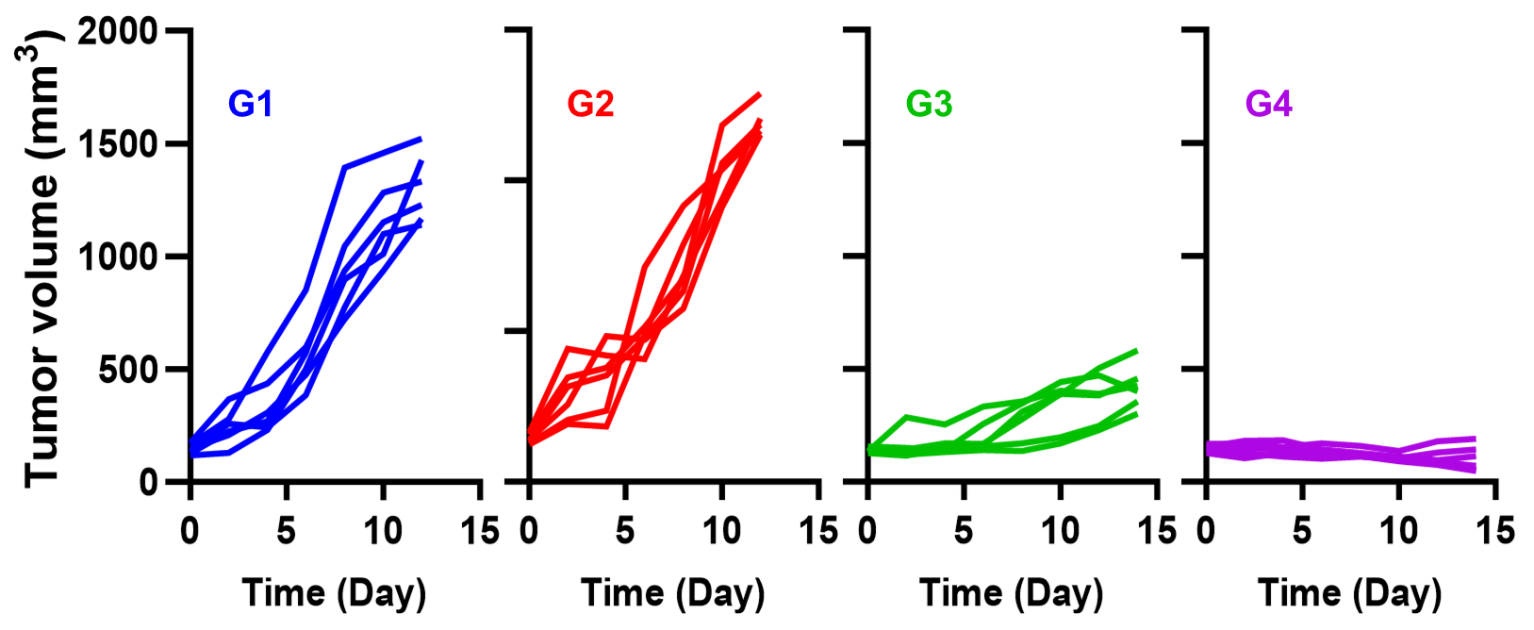
**

**Figure S39.** Individual growth curves of H22 tumors after various treatments. G1: Ctrl., G2: LOx, G3: CoF_2_, G4: LOCoF_2_.

**
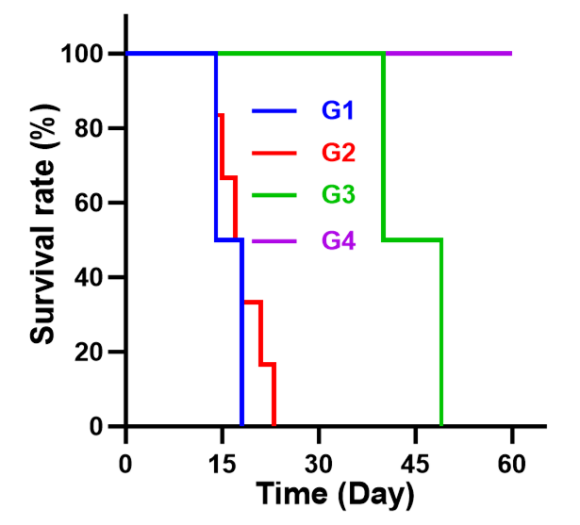
**

**Figure S40.** Survival rate curves of the mice after 60 days. G1: Ctrl., G2: LOx, G3: CoF_2_, G4: LOCoF_2_.

**
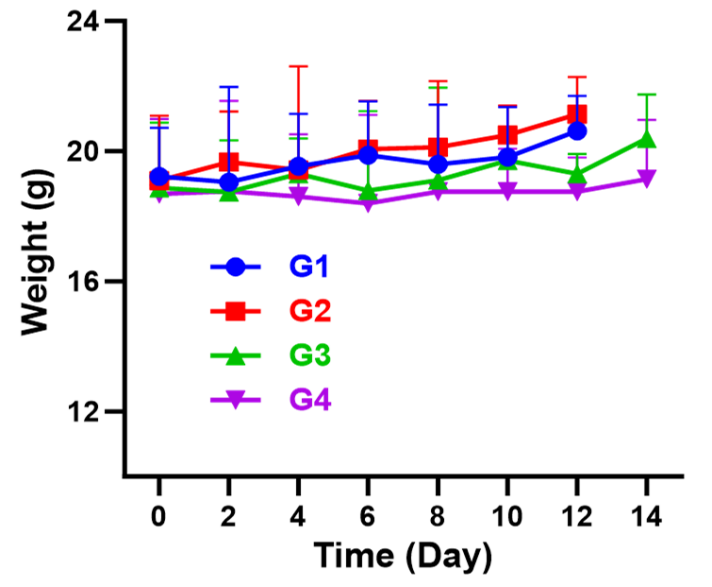
**

**Figure S41.** Changes in the body weight of tumor-bearing mice after different treatments. G1: Ctrl., G2: LOx, G3: CoF_2_, G4: LOCoF_2_.

**
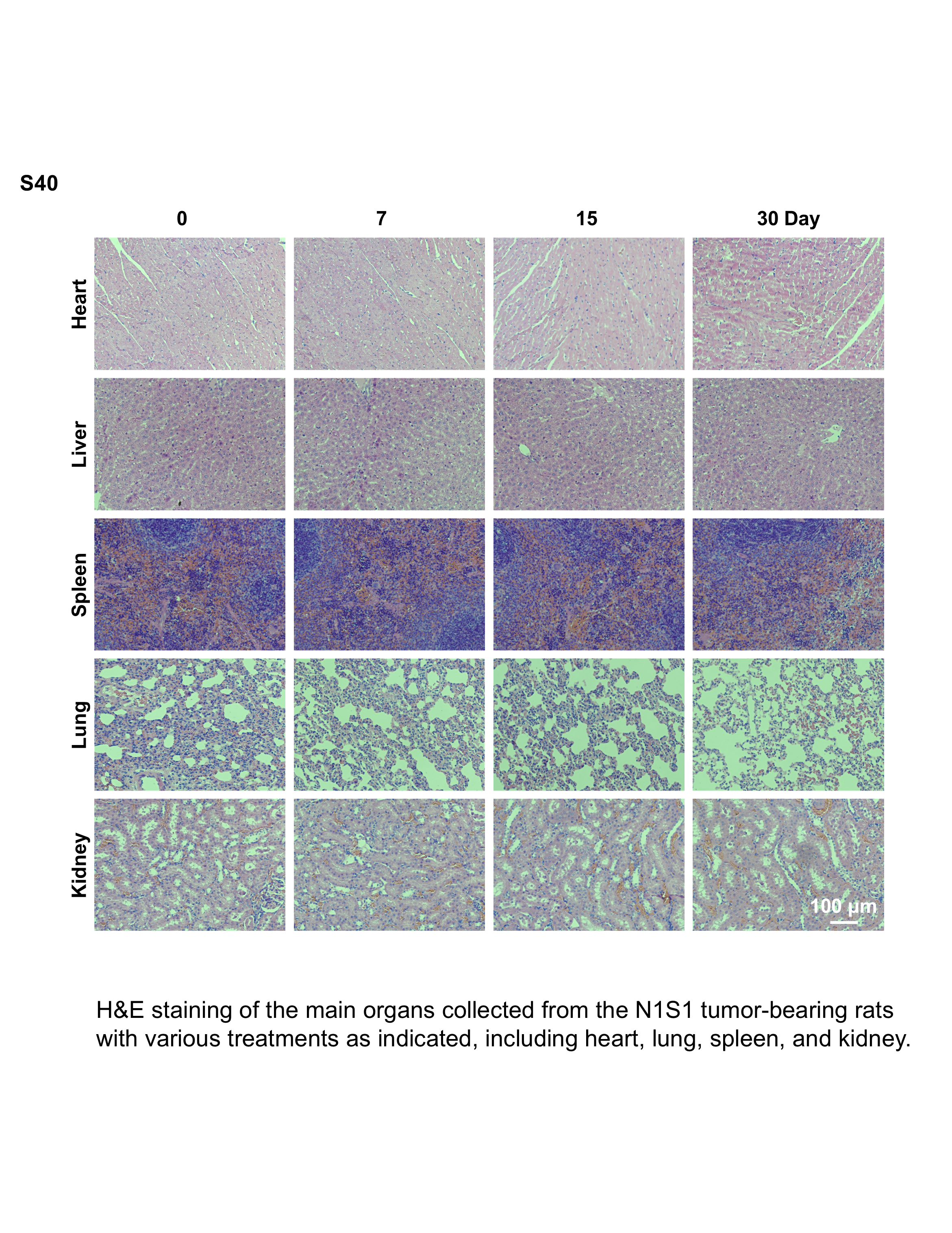
**

**Figure S42.** H&E-staining of the main organs, including heart, liver, spleen, lung, and kidney, from LOCoF_2_ treatment.

**
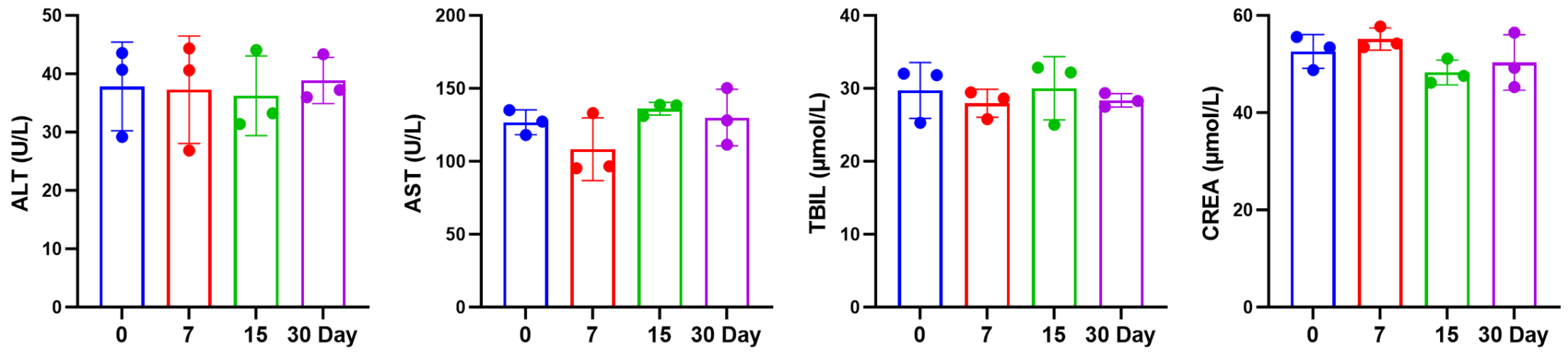
**

**Figure S43.** Blood biochemistry data of the mice treated with LOCoF_2_ at various time points (0, 3, 7, 15, and 30 Days). Alanine aminotransferase (ALT), aspartate aminotransferase (AST), total bilirubin (TBIL), creatinine (CREA).

**
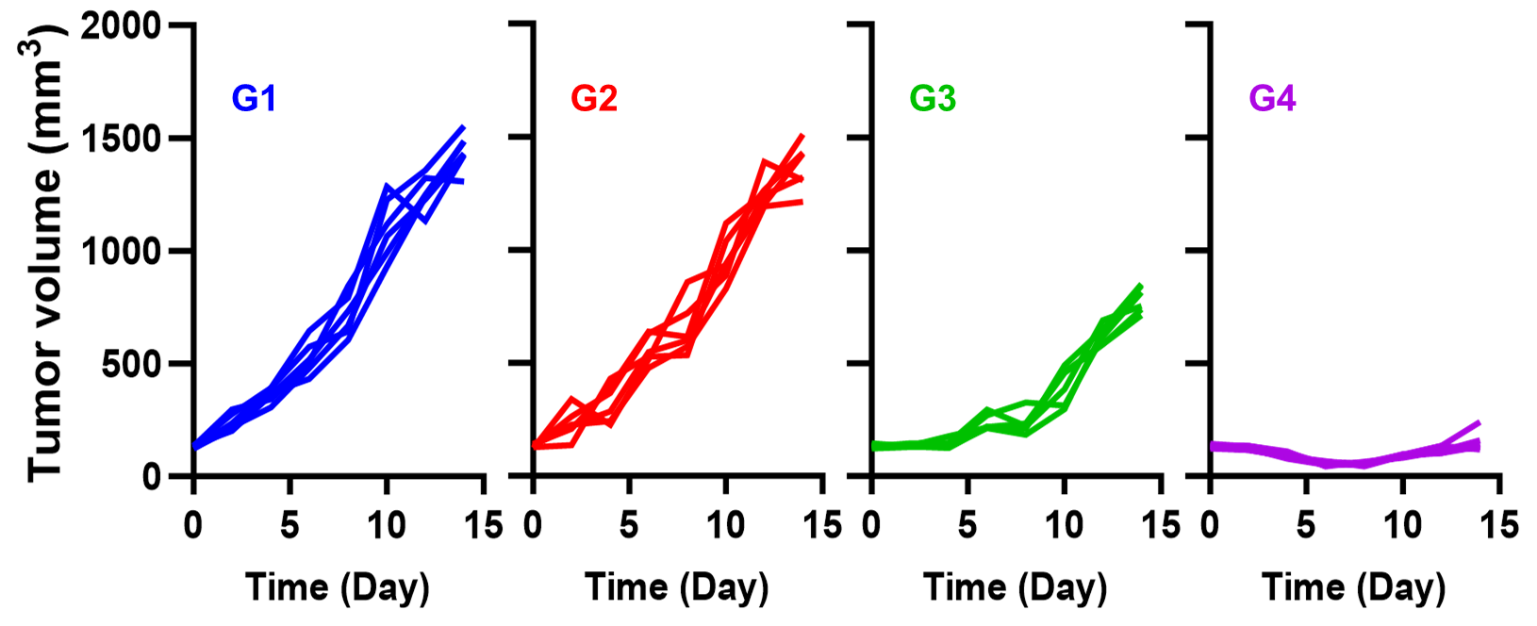
**

**Figure S44.** Individual growth curves of HuH7 tumors after various treatments. G1: Ctrl., G2: LOx, G3: CoF_2_, G4: LOCoF_2_.

**
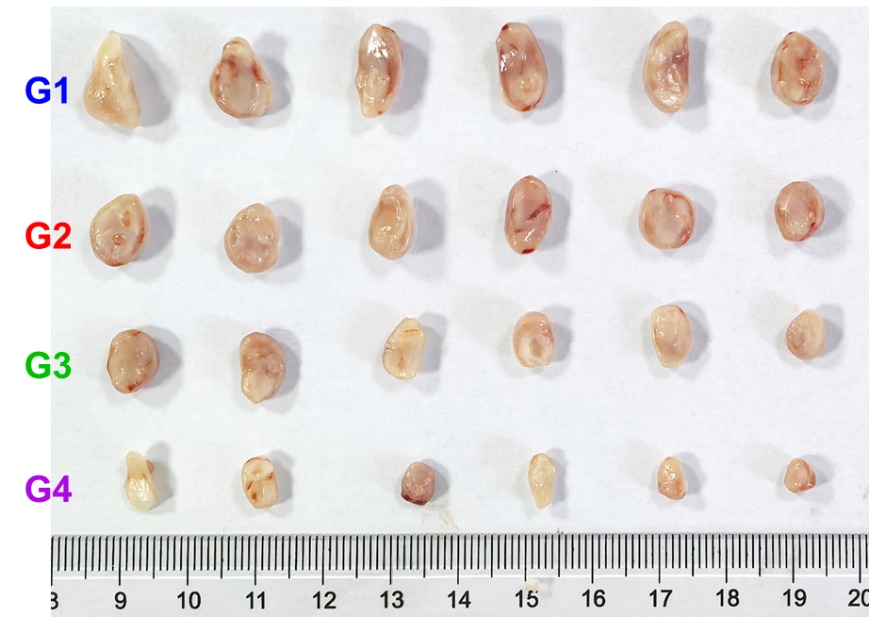
**

**Figure S45.** Images of HuH7 nude mice with subcutaneous tumors 14 days after receiving various treatments. G1: Ctrl., G2: LOx, G3: CoF_2_, G4: LOCoF_2_.


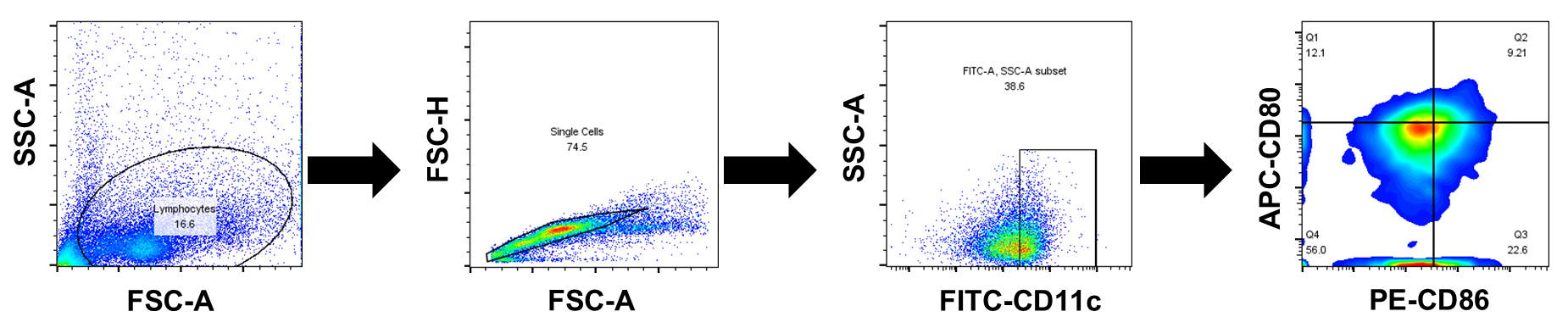


**Figure S46.** The gating strategy used to determine the percentages of DC cells (CD11c^+^CD80^+^CD86^+^) in **Figure 5E**.

**
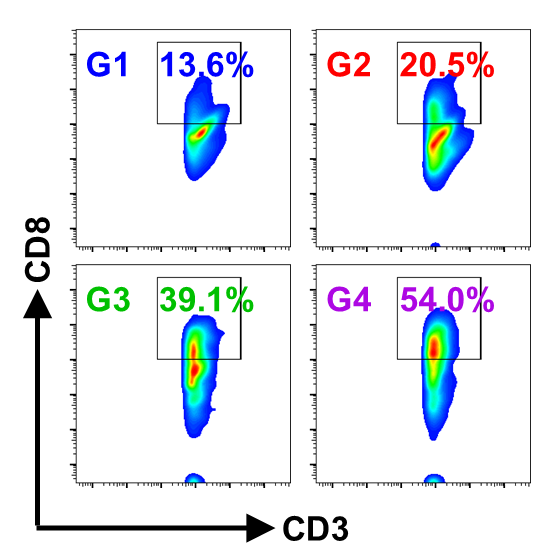
**

**Figure S47.** Flow cytometry analysis of CD45^+^CD3^+^CD8^+^ T cells. G1: Ctrl., G2: LOx, G3: CoF_2_, G4: LOCoF_2_.

**
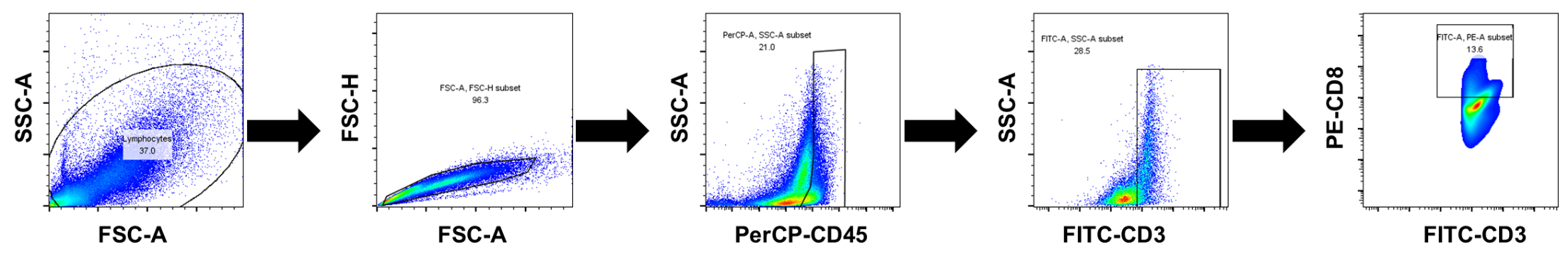
**

**Figure S48.** The gating strategy used to determine the percentages of T cells (CD45^+^CD3^+^CD8^+^) in **Figure S47**.

**
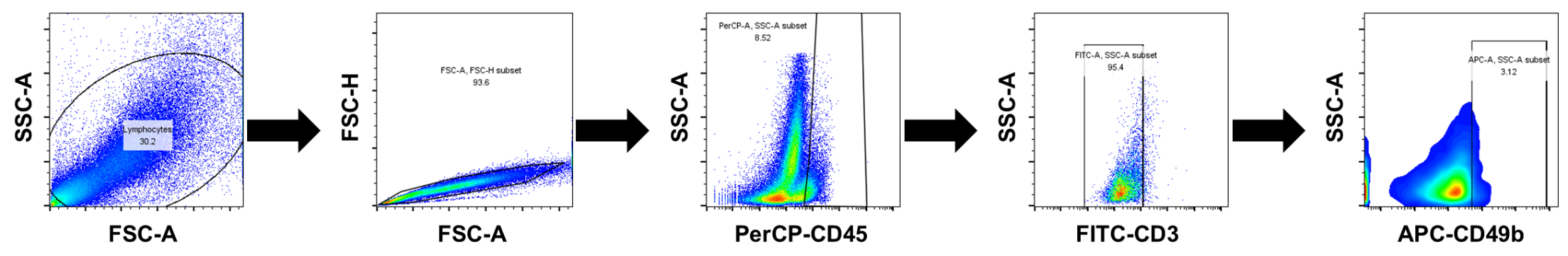
**

**Figure S49.** The gating strategy used to determine the percentages of NK cells (CD45^+^CD3^-^CD49b^+^) in **Figure 5H.**

**
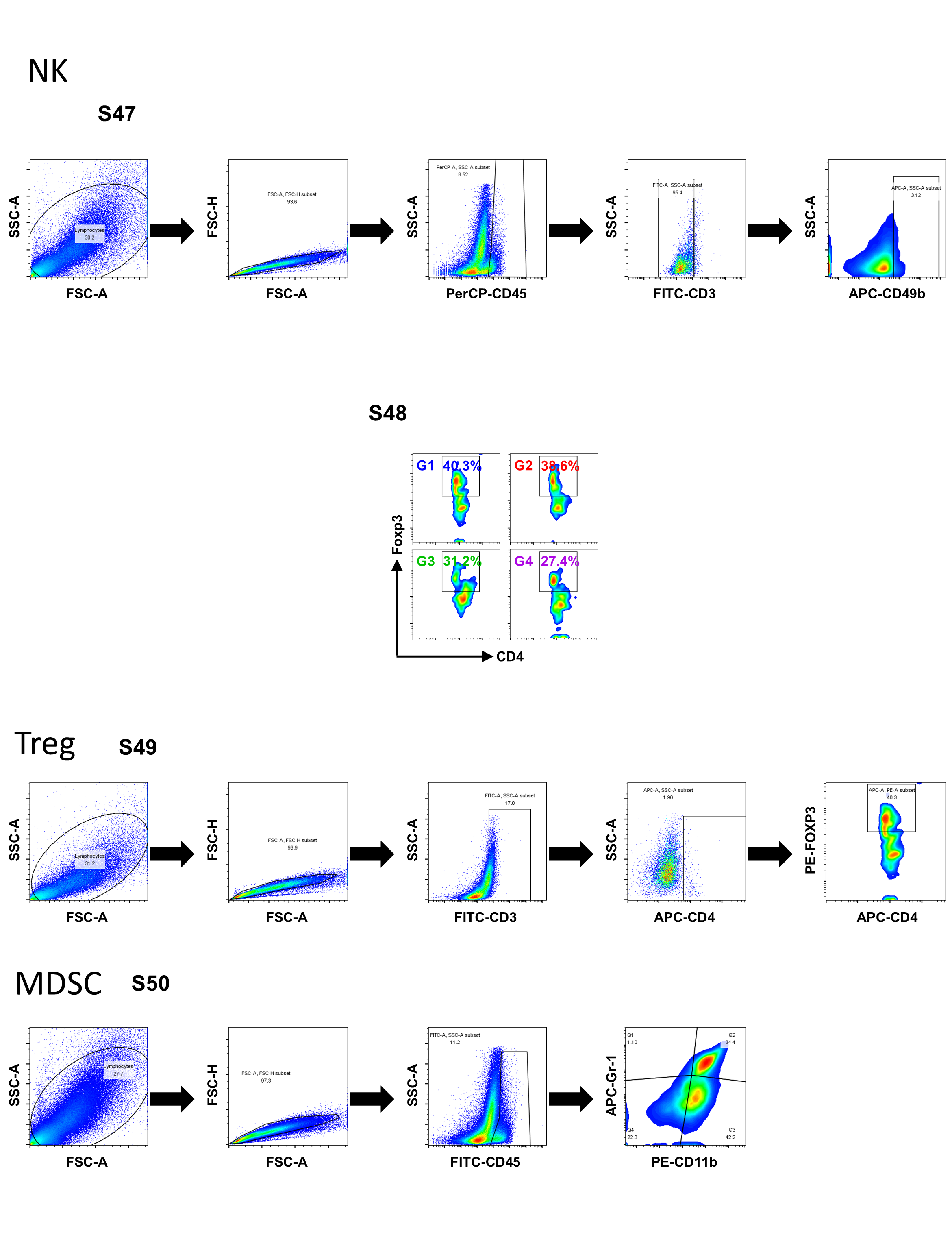
**

**Figure S50.** Flow cytometry analysis of CD3^+^CD4^+^Foxp3^+^ cells. G1: Ctrl., G2: LOx, G3: CoF_2_, G4: LOCoF_2_.

**
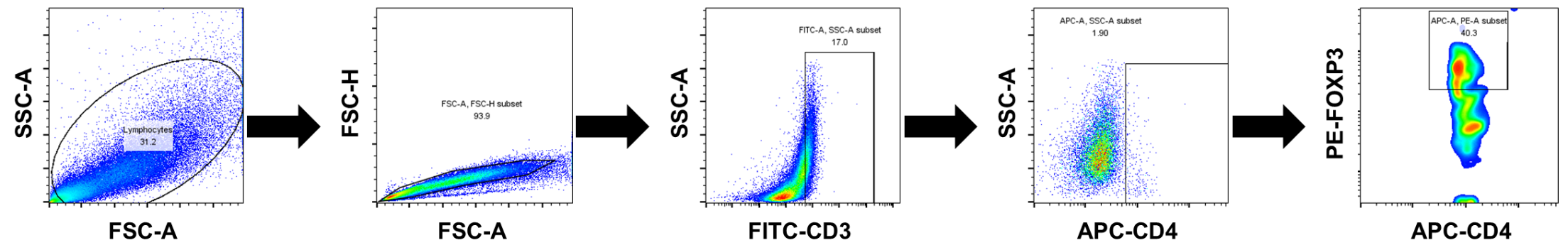
**

**Figure S51.** The gating strategy used to determine the percentages of Tregs (CD3^+^CD4^+^Foxp3^+^) in **Figure S50**.

**
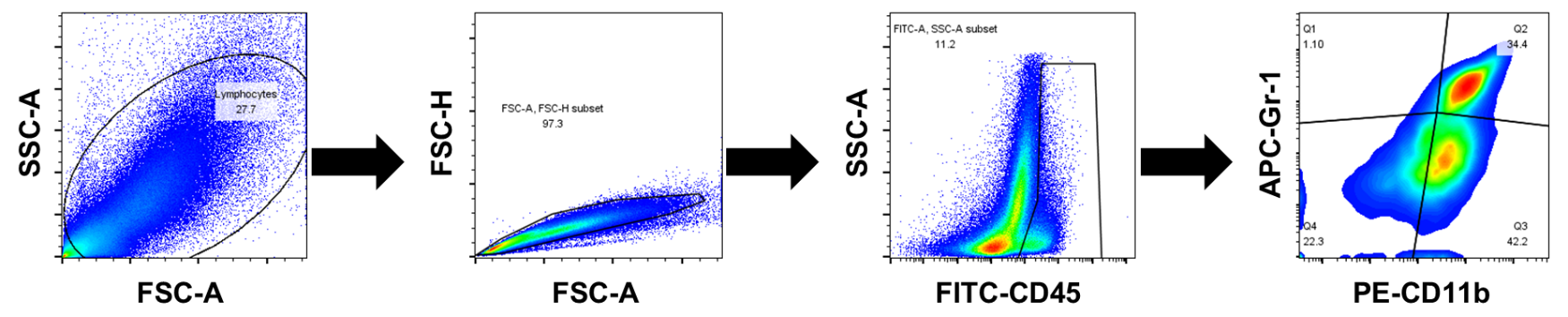
**

**Figure S52.** The gating strategy used to determine the percentages of MDSC cells (CD45^+^Gr-1^+^CD11b^+^) in **Figure 5K**.

**
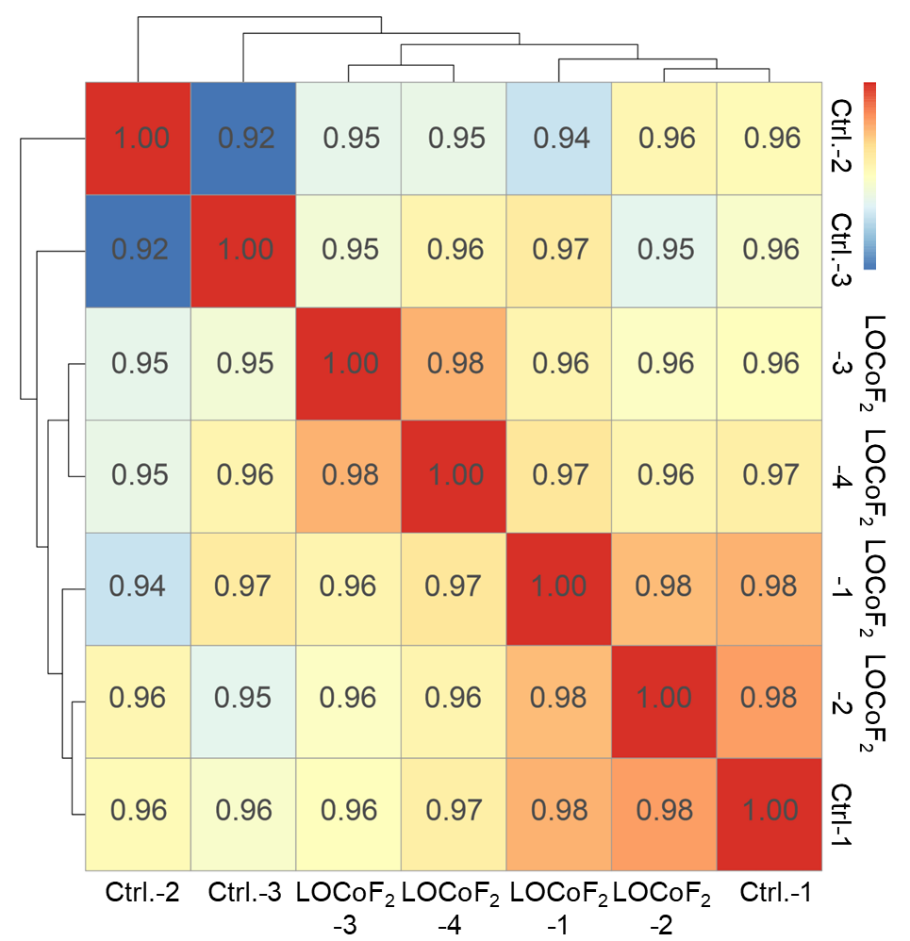
**

**Figure S53.** Unsupervised hierarchical clustering of the RNA-Seq data from the control groups and LOCoF_2_ groups.

**
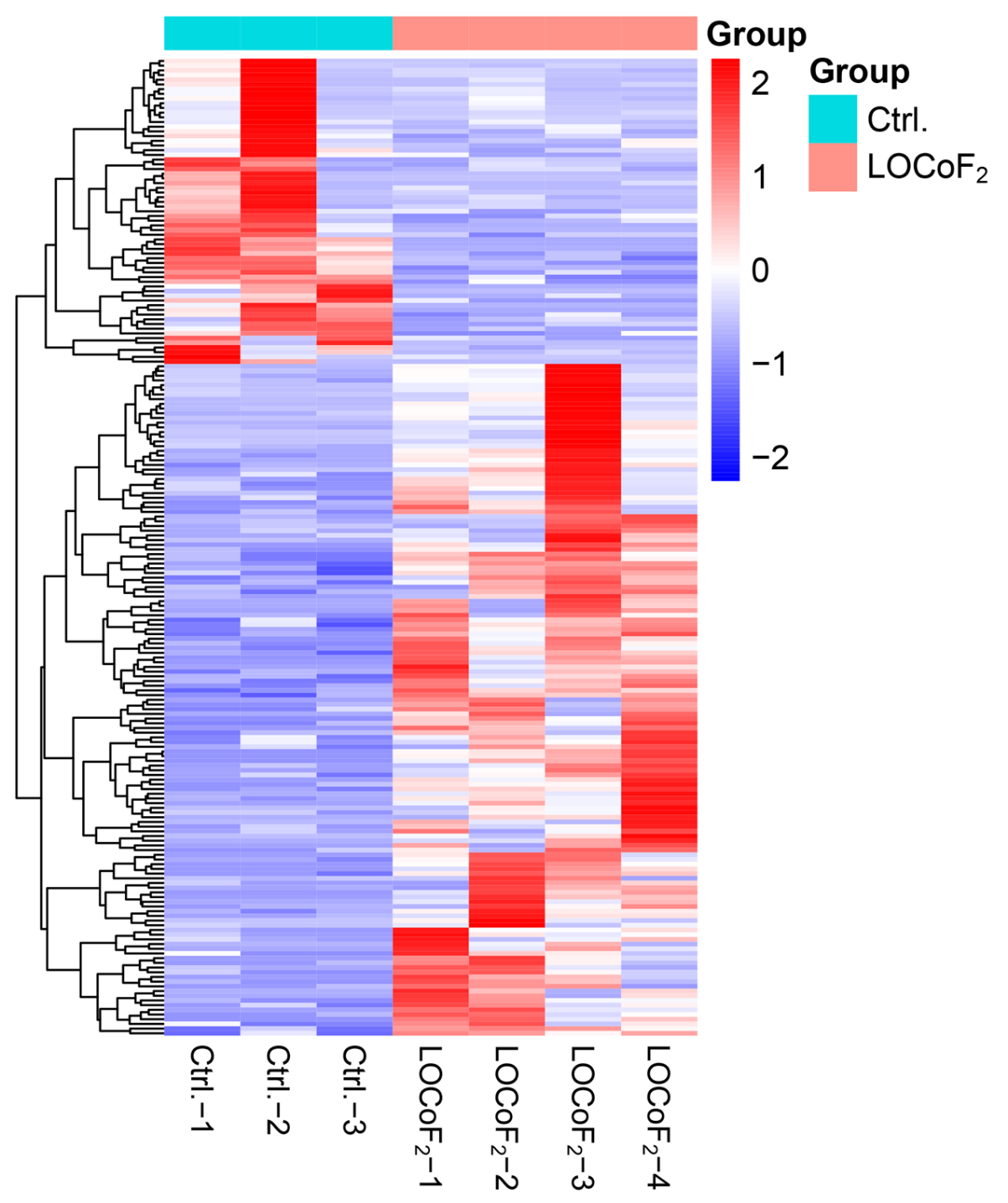
**

**Figure S54.** Cluster diagram of differentially expressed genes (DEGs) between the control group and the LOCoF_2_ group.

**
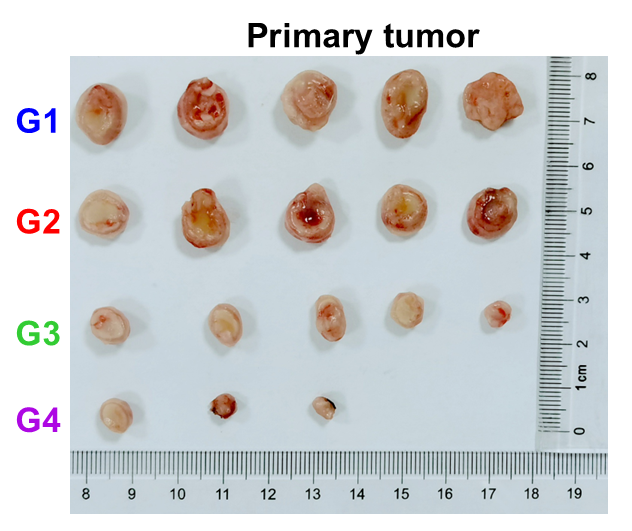
**

**Figure S55.** Images of H22 primary tumor-bearing mice 10 days after various treatments. G1: Ctrl., G2: α-PD-1, G3: LOCoF_2_, G4: LOCoF_2_ + α-PD-1.

**
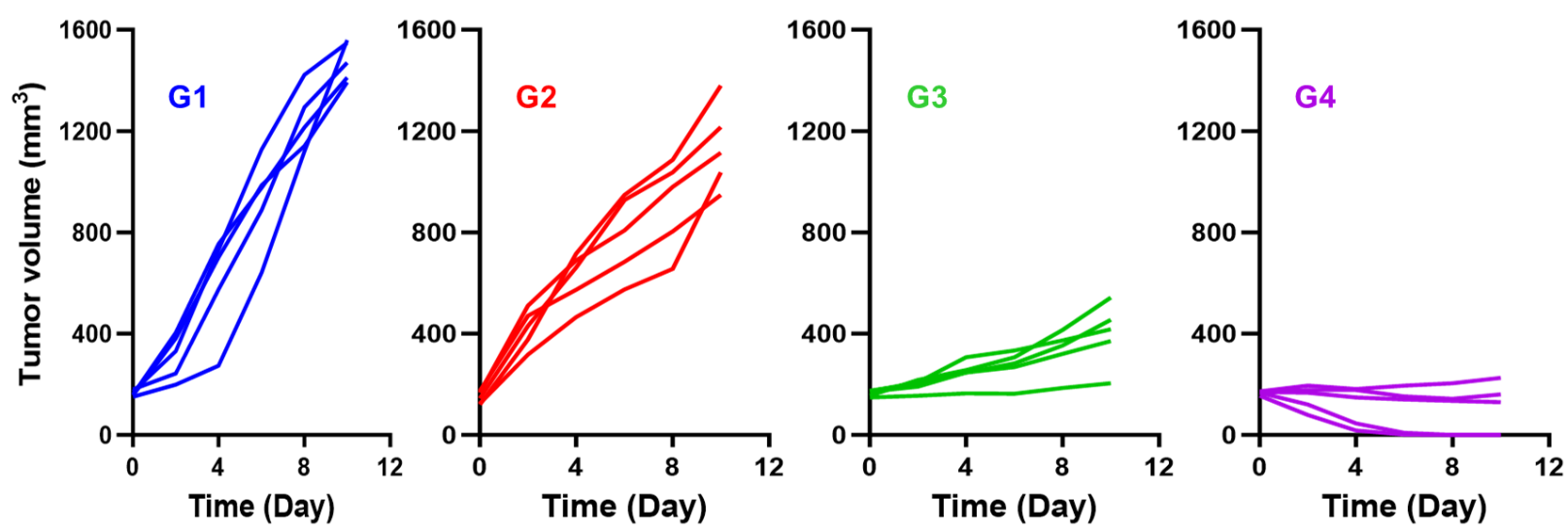
**

**Figure S56.** Individual growth curves of H22 primary tumors after various treatments. G1: Ctrl., G2: α-PD-1, G3: LOCoF_2_, G4: LOCoF_2_ + α-PD-1.

**
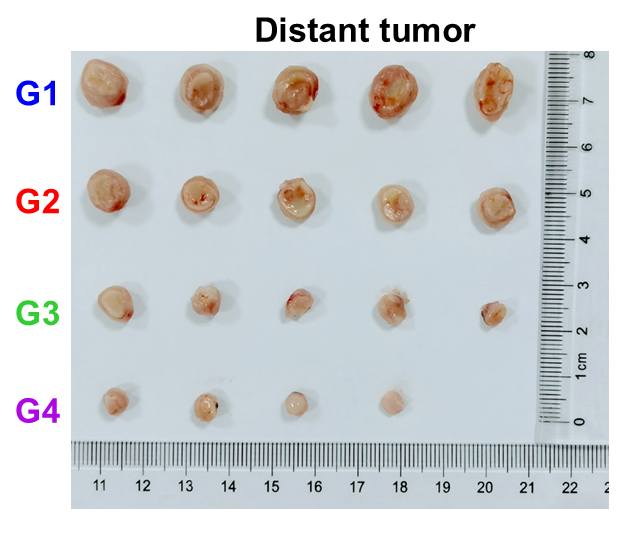
**

**Figure S57.** Images of H22 distant tumor-bearing mice 10 days after various treatments. G1: Ctrl., G2: α-PD-1, G3: LOCoF_2_, G4: LOCoF_2_ + α-PD-1.

**
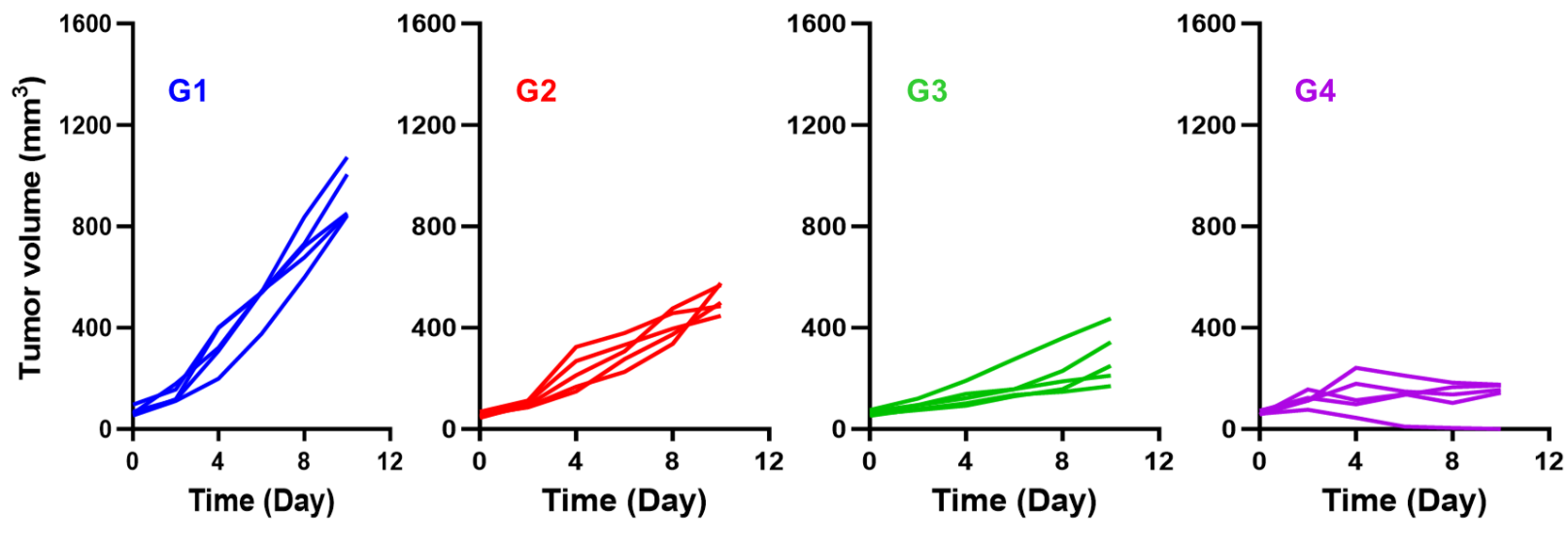
**

**Figure S58.** Images of H22 distant tumor-bearing mice at 10 days after various treatments. G1: Ctrl., G2: α-PD-1, G3: LOCoF_2_, G4: LOCoF_2_ + α-PD-1.

**
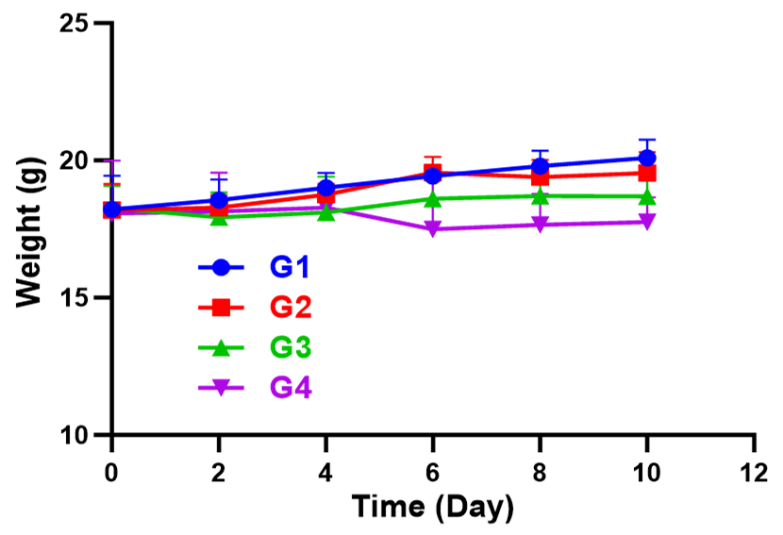
**

**Figure S59.** Changes in the body weights of tumor-bearing mice after different treatments. G1: Ctrl., G2: α-PD-1, G3: LOCoF_2_, G4: LOCoF_2_ + α-PD-1.
